# Targeting TIMM23 to overcome osteosarcoma chemoresistance

**DOI:** 10.1038/s41419-025-08106-w

**Published:** 2025-11-24

**Authors:** Zhiwei Tao, Pingan Zou, Zhengxu Yang, Tao Xiong, Zhi Deng, Qinchan Chen

**Affiliations:** Bone and Soft Tissue Sarcoma Department, Jiangxi Cancer Hospital, Nanchang, PR China

**Keywords:** Bone cancer, Bone cancer

## Abstract

Osteosarcoma (OS) is a malignant tumor whose chemoresistance severely compromises therapeutic efficacy. This study aims to investigate the molecular mechanism by which TIMM23 mediates M2 polarization of macrophages through mitophagy and regulates TIMM23-PARGP1 fusion gene expression and chemoresistance in OS. Single-cell transcriptomic analysis revealed a strong interaction between macrophages and tumor cells. By integrating bulk RNA sequencing data and weighted gene co-expression network analysis (WGCNA) co-expression network analysis, we identified TIMM23 as a key gene associated with macrophage polarization. Using STAR-Fusion, we further detected the TIMM23-PARGP1 fusion gene, which was validated via fluorescence in situ hybridization (FISH). CRISPR/Cas9 was employed to generate TIMM23-knockout macrophages, and a lentiviral system was used to create TIMM23-overexpressing macrophages. Flow cytometry demonstrated that TIMM23 promotes M2 polarization, while confocal and transmission electron microscopy confirmed its role in regulating mitophagy in M2 macrophages. These findings indicate that TIMM23 promotes M2 polarization through mitophagy modulation. Next, a co-culture model of macrophages and OS cells was established. MTT, Cell Counting Kit-8 (CCK-8), EdU, Transwell, and TUNEL assays were performed to evaluate tumor cell behavior and chemosensitivity. The results showed that TIMM23-induced M2 polarization upregulated TIMM23-PARGP1 expression in OS cells, thereby enhancing their proliferation, migration, and invasion while inhibiting apoptosis and reducing the effectiveness of chemotherapeutic agents. In vivo experiments further confirmed the role of TIMM23 in promoting M2 polarization and fusion gene expression, leading to increased chemoresistance and tumor growth. In conclusion, TIMM23 enhances OS chemoresistance and tumor progression by promoting M2 macrophage polarization via mitophagy and upregulating TIMM23-PARGP1 fusion gene expression.

**Figure:**
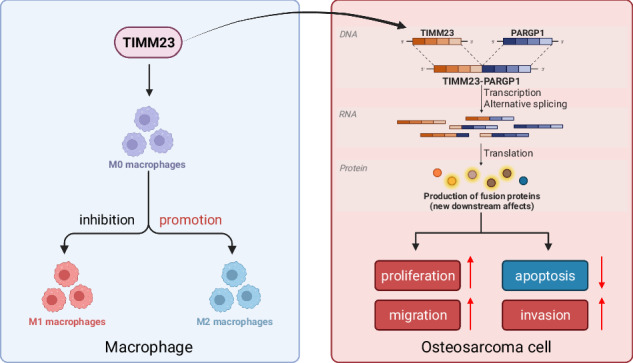
Graphical Abstract. Molecular mechanism diagram illustrating the involvement of TIMM23 in mitophagy-mediated M2 polarization of macrophages and the impact of TIMM23-PARGP1 fusion gene on chemoresistance in OS (Created with BioRender.com).

Graphical Abstract. Molecular mechanism diagram illustrating the involvement of TIMM23 in mitophagy-mediated M2 polarization of macrophages and the impact of TIMM23-PARGP1 fusion gene on chemoresistance in OS (Created with BioRender.com).

## Introduction

Osteosarcoma (OS) is a malignant tumor originating from bone or soft tissue, characterized by its high invasiveness and strong metastatic potential. It has long posed a major challenge in tumor therapy. The disease exhibits a particularly high incidence among children and adolescents, severely endangering their health and survival [1–5]. Currently, the treatment of OS mainly includes surgical resection, radiotherapy, and chemotherapy as comprehensive therapeutic approaches [6–8]. Although these treatment methods have, to some extent, improved the survival rate of OS patients, the issue of chemotherapy resistance remains a major challenge in current treatments [9, 10]. Therefore, there is an urgent need to investigate the mechanisms underlying OS progression and explore novel therapeutic strategies.

Cellular alterations within the tumor microenvironment (TME) play a pivotal role in the malignant progression of OS [11, 12]. Single-cell transcriptomics has emerged as a powerful tool to uncover cellular heterogeneity and expression profiles of tumor subpopulations, thereby facilitating the understanding of how specific cell types contribute to tumor development [13–15]. In recent years, several studies employing this technology have systematically characterized the cellular composition and dynamic states within primary, recurrent, and metastatic OS tissues. For instance, one study identified transdifferentiation events among malignant cell types and revealed a characteristic infiltration of FABP4⁺ pro-inflammatory macrophages in lung metastases [16]. Another study discovered a population of cancer-associated fibroblasts (CAFs) in recurrent OS that modulate the immune microenvironment via high LOX expression [17]. Meanwhile, mitophagy—an essential cellular metabolic regulatory mechanism—plays a critical role in maintaining cellular homeostasis, modulating energy metabolism, and promoting cell survival [18, 19]. Accumulating evidence suggests that mitophagy contributes to tumorigenesis, stress adaptation, and therapeutic resistance [18, 20]. For example, in ovarian cancer, targeting the CRL4 E3 ubiquitin ligase complex has been shown to enhance mitophagy via regulation of mitochondrial fission and activation of the PINK1/Parkin pathway, thereby increasing chemosensitivity [21]. These findings highlight the potential value of targeting mitophagy to overcome drug resistance. Therefore, integrating single-cell transcriptomics with analyses of mitophagy activity in the TME may yield novel insights into OS progression and chemoresistance and provide new therapeutic targets.

Tumor-associated macrophages (TAMs) are key immune components of the TME, typically categorized into pro-inflammatory M1 and immunosuppressive M2 phenotypes. M2 macrophages have been widely associated with tumor immune evasion, invasion, and resistance to chemotherapy, often correlating with poor prognosis in various cancers [22, 23]. Similarly, in OS, M2 macrophage enrichment is closely linked to poor patient outcomes. Previous studies have demonstrated that inhibition of the PI3K/AKT pathway suppresses M2 polarization, thereby limiting tumor proliferation, migration, and metastasis [24]. Additionally, TAMs can release inflammatory cytokines such as TNF-α and IL-6 in response to environmental cues, activating the STAT3 signaling pathway and promoting chemoresistance in OS [25]. Notably, recent studies have implicated mitophagy as a regulatory mechanism in M2 macrophage polarization [26, 27]. However, the key regulators involved in this process in OS remain largely undefined, and the potential link between mitophagy-driven macrophage polarization and chemoresistance requires further elucidation.

To address this, our study combined single-cell transcriptomic analysis, co-expression network construction, and fusion gene screening to systematically identify key regulators associated with M2 macrophage polarization. We identified the mitochondrial protein TIMM23 and its fusion variant TIMM23-PARGP1 as critical mediators. Through in vitro and in vivo models, we validated their functional roles in immune modulation and chemoresistance. By focusing on TIMM23, we aim to elucidate the mechanism by which it regulates macrophage polarization and promotes immune evasion and chemoresistant phenotypes in OS. Our findings not only establish TIMM23 as a potential therapeutic target in OS but also provide mechanistic insights that may inform immunotherapy strategies across diverse cancer types.

## Results

### single-cell RNA sequencing (scRNA-seq) reveals the composition and characteristics of cell subpopulations in tumor tissue

**Fig. 1:**
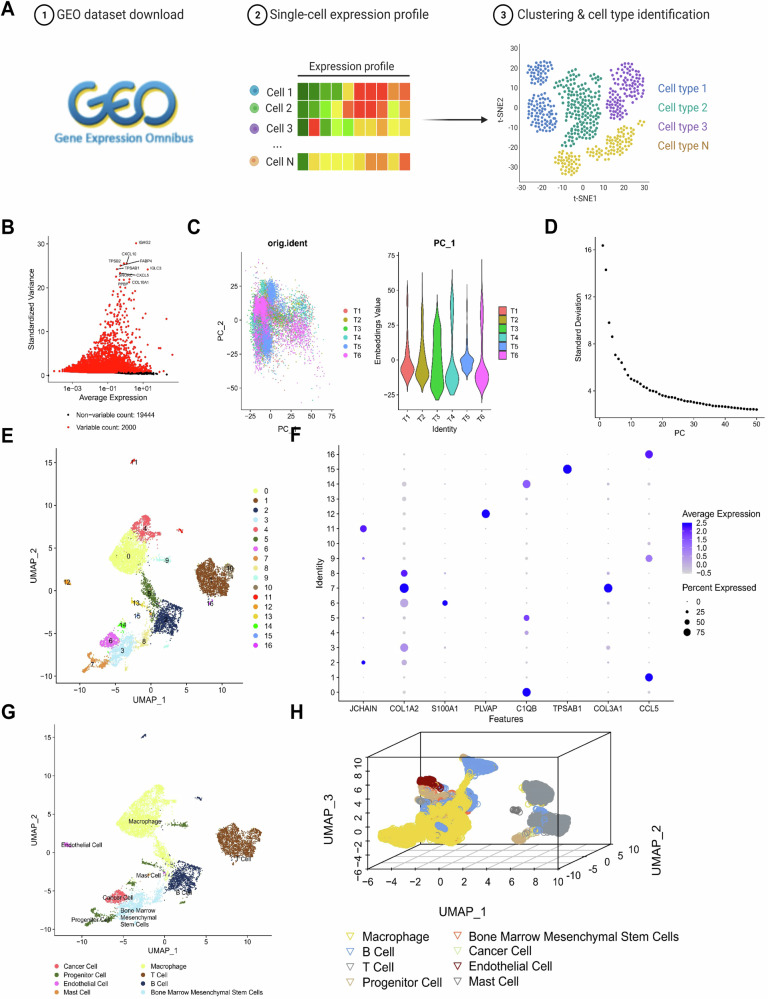
scRNA-seq data cell clustering and annotation. **A** Schematic diagram of single-cell data sample acquisition and analysis workflow; **B** Differential expression analysis for highly variable genes. Red represents the top 2000 highly variable genes, black represents low variable genes, and the top 10 genes in the highly variable gene set are labeled (N = 6); **C** Distribution of cells in PC_1 and PC_2 in PCA analysis. Each point represents a cell (N = 6); **D** Distribution of standard deviation of PCs. Important PCs have a larger standard deviation (N = 6); **E** UMAP visualization of cell clustering and distribution in OS samples (N = 6). Each color represents a cluster; **F** Expression pattern of known cell lineage-specific marker genes in different cell types of OS samples (N = 6). Deeper purple represents a higher average expression level, and larger circles represent more cells expressing the gene; **G** Visualization of cell annotation results in OS samples (N = 6) based on UMAP clustering. Each color represents a cell subgroup; **H** 3D visualization of cell clustering and distribution in OS samples (N = 6) based on UMAP clustering.

To further investigate the roles of different cellular subtypes in the development of OS and the TME, we obtained single-cell transcriptome sequencing data related to OS from the GSE162454 dataset in the GEO database. This dataset included primary OS tissue samples from 6 different patients (Fig. 1A). We integrated the sequencing data using the Seurat package and initially examined the number of genes (nFeature_RNA), mRNA molecules (nCount_RNA), and percentage of mitochondrial genes (percent.mt) in all cells from the scRNA-seq data (Fig. S1A). Subsequently, we performed quality control on the raw data by removing cells with fewer than 200 or more than 5000 detected genes (nFeature_RNA), as well as those with a mitochondrial gene percentage (percent.mt) greater than 25%. After filtering, we obtained an expression matrix comprising 21,444 genes and 10,768 cells. The results of the sequencing depth correlation analysis showed a correlation coefficient of r = 0.16 between nCount_RNA and percent.mt of the filtered cell data, a correlation coefficient of r = 0.86 between nCount_RNA and nFeature_RNA, and a correlation coefficient of r = −0.02 between nCount_RNA and percent.HB (Fig. S1B) indicates the high quality of the filtered cell data.

Further analysis of the filtered cells involved selecting highly variable genes based on gene expression variance, with the top 2000 genes chosen for downstream analysis (Fig. 1B). We calculated the cell cycle of the cell samples using the CellCycleScoring function (Fig. S1C) and performed preliminary normalization of the data. Subsequently, we employed PCA to perform linear dimensionality reduction on the data and generated a PCA plot (Fig. 1C). Here, we presented a heatmap of the main correlated gene expressions for PC_1–PC_6 (Fig. S1D). We used the harmony package for batch correction of the sample data (Fig. S1E, F) and performed a standard deviation ranking of the PCs using ElbowPlot (Fig. 1D). The results showed that PCA revealed a distinct “elbow” around the 30th principal component, indicating that beyond this point, the explained variance dropped substantially. Therefore, we selected PC_1–PC_30 for downstream analyses to retain meaningful variation while minimizing noise.

Next, we performed nonlinear dimensionality reduction on the top 30 principal components (PCs) using the uniform manifold approximation and projection (UMAP) algorithm and conducted clustering analysis with a resolution of 0.4 (Fig. S2). This analysis identified 17 distinct clusters, and we obtained the marker gene expression profiles for each cluster (Fig. 1E). Based on known lineage-specific marker genes retrieved from the literature and annotations provided by the CellMarker online database, we classified the cells into eight distinct cell types: B cells, bone marrow mesenchymal stem cells, cancer cells, endothelial cells, macrophages, mast cells, progenitor cells, and T cells (Fig. 1F, Fig. S1G). Among these, macrophages represented the highest proportion, accounting for nearly 30% of all cells, indicating that macrophages are the most abundant cell type in OS tissues (Fig. 1G, H).

Overall, these results indicate that OS samples, along with their adjacent normal samples, can be classified into 17 clusters, encompassing eight cell subpopulations, with macrophages being the most abundant in OS tissue.

### Macrophage polarization regulation: discovery of the key gene TIMM23 in OS

**Fig. 2:**
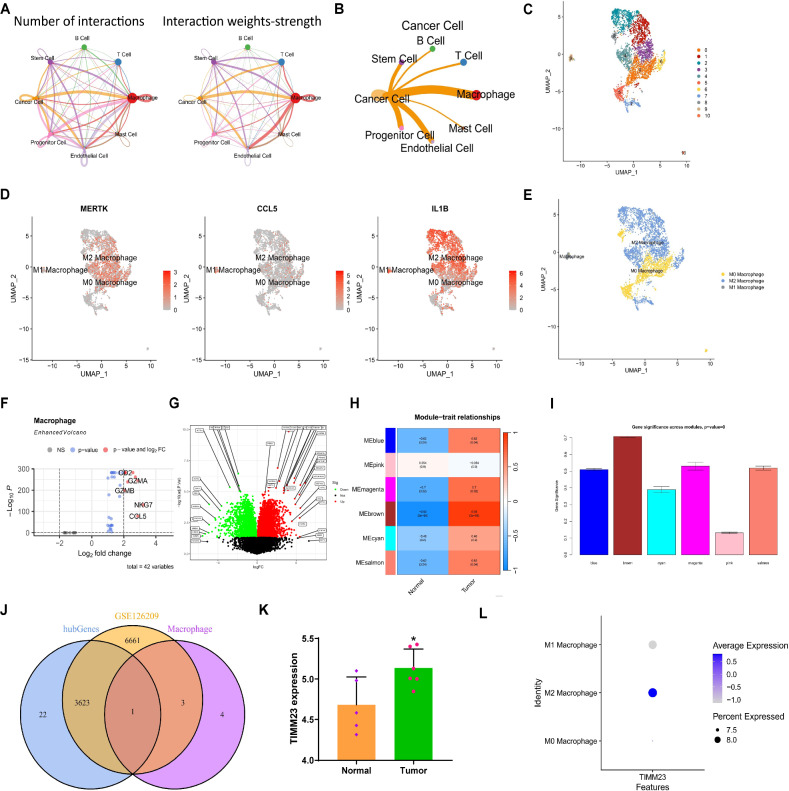
Joint screening of OS-related genes from single-cell transcriptomes. **A** Circular plot of cell communication differences in OS samples (N = 6), where the thickness of the lines on the left represents the number of pathways, and the thickness of the lines on the right represents the interaction intensity; **B** Differential cell communication plot of Cancer Cells in OS samples, where the thickness of the lines represents the interaction intensity (N = 6); **C** UMAP visualization showing the clustering and distribution of Macrophage subtypes in OS samples (N = 6), where each color represents a cluster; **D** Expression pattern of 8 cell type marker genes in different cell subgroups of OS samples, with darker red indicating higher average expression level; **E** Visualization of cell annotation results in OS samples (N = 6) based on UMAP clustering, where each color represents a cell subgroup; **F** Volcano plot of DEGs between M1 Macrophages and M2 Macrophages in OS tissue, with points on the right of the dashed line indicating genes highly expressed in M2 samples (N = 6); **G** Volcano plot of differentially expressed mRNAs between 6 cases of OS tumor tissue and 5 matched adjacent normal tissues from the GSE126209 dataset; **H** Analysis results of WGCNA co-expression analysis and OS disease correlation (N = 11); **I** Analysis results of WGCNA co-expression analysis of gene importance in OS, where the height of the module represents the importance of module genes in OS disease; **J** Venn diagram showing the intersection of significantly DEGs in M1 Macrophages and M2 Macrophages, differential analysis results from the GSE126209 dataset, and WGCNA analysis results; **K** Differential expression of TIMM23 in GSE126209, where Tumor and Normal represent 6 cases of OS tumor tissue and 5 matched adjacent normal tissue samples, respectively; **L** Expression pattern of TIMM23 in M1 Macrophages and M2 Macrophages. Blue points in the volcano plot represent significantly downregulated mRNA in OS tissue, red points represent significantly upregulated mRNA in OS tissue, and gray points represent mRNA with no significant difference.

Subsequently, to investigate the intercellular interaction between different cell types, we utilized the R package “CellChat” to explore pathway activities among various cells. The results revealed that the interaction between Macrophages and Cancer cells was the closest and the strongest in the OS tissue (Fig. 2A, B).

We extracted the single-cell data of Macrophages and performed nonlinear dimensionality reduction using the UMAP algorithm. For cluster analysis, we selected a resolution of 0.4 (Fig. S3). Through cluster analysis, we obtained 11 clusters and identified marker gene expression levels for each cluster (Fig. 2C). Additionally, we annotated the cells (Fig. 2D) and categorized Macrophages into three cell types: M0 Macrophage, M1 Macrophage, and M2 Macrophage (Fig. 2E). M1 Macrophages release pro-inflammatory cytokines and exhibit protective responses, contributing to antimicrobial or anti-tumor activities. On the other hand, M2 Macrophages release anti-inflammatory cytokines that support tumor growth, invasive capabilities, and metastatic potential. Interestingly, the roles of these two subtypes of Macrophages are reversed in OS [28, 29]. Hence, employing |log2FC| > 2 and *P* < 0.05 as screening criteria, we identified 9 genes that exhibited significant differential expression between M1 and M2 Macrophages (Fig. 2F). We performed functional enrichment analysis on the differentially expressed genes (DEGs). Gene Ontology (GO) analysis revealed significant enrichment in terms such as “immune system process” (−log₁₀(p) ≈ 4.5), “regulation of macrophage apoptotic process” (−log₁₀(p) ≈ 3.2), and “protein targeting to mitochondrion” (−log₁₀(p) ≈ 3.1) (Fig. S4A). KEGG pathway analysis showed enrichment in pathways such as “Cytokine-cytokine receptor interaction” (−log₁₀(p) ≈ 2.0), “Primary immunodeficiency” (−log₁₀(p) ≈ 1.5), and “Apoptosis” (−log₁₀(p) ≈ 1.3) (Fig. S4B).

To further identify key genes closely related to OS, we obtained the OS-related RNA-seq data from the GEO database, specifically the GSE126209 dataset, which comprised 6 OS tumor tissue samples and 5 adjacent normal tissue samples. Differential analysis revealed significant differential expression of 10,289 mRNAs in OS tissue compared to normal adjacent tissue (Fig. 2G). Subsequently, we performed weighted gene co-expression network analysis (WGCNA) co-expression analysis on the RNA-seq data and obtained 6 gene modules (Fig. 2H, I). Among them, the brown module (cor = 0.93, *p* = 3e − 05), blue module (cor = 0.62, *p* = 0.04), magenta module (cor = 0.7, *p* = 0.02), and salmon module (cor = 0.62, *p* = 0.04) showed strong positive correlations with tumor tissues and negative correlations with normal tissues, indicating that genes within these modules are likely associated with the occurrence and progression of OS.

By intersecting the genes with significant differential expression in M1 Macrophages and M2 Macrophages, the differential analysis results of the RNA-seq data, and the WGCNA analysis results, we finally obtained 1 gene in the intersection: TIMM23 (Fig. 2J). The expression pattern of TIMM23 in GSE126209 is shown in Fig. 2K, with significant expression in M2 Macrophages and lower expression in M1 Macrophages. Therefore, it is speculated that TIMM23 may play an important role in regulating macrophage polarization (Fig. 2L).

In conclusion, through the combined analysis of single-cell transcriptomics, we identified 1 key gene, TIMM23, in OS. It is hypothesized that TIMM23 may play a crucial role in the regulation of macrophage polarization.

### TIMM23 regulation of macrophage M2 polarization through mediating mitophagy

**Fig. 3:**
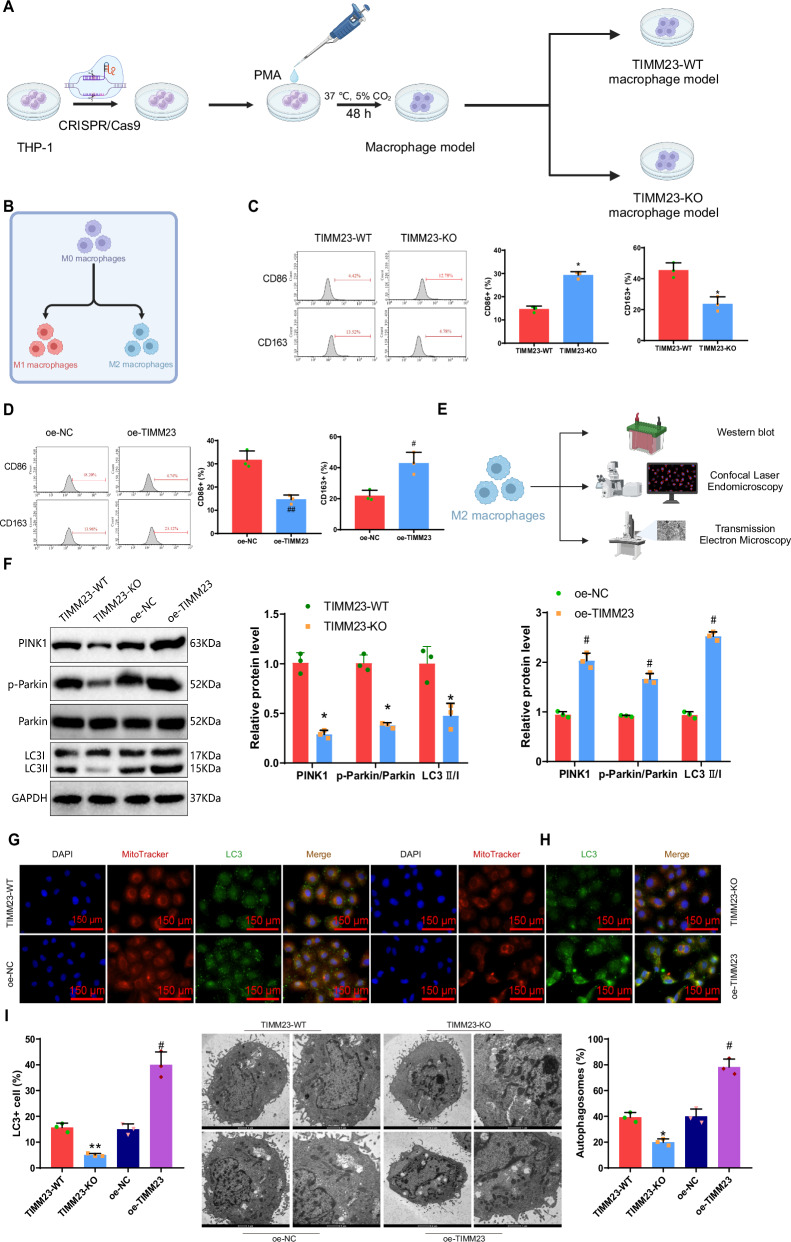
TIMM23-mediated mitophagy regulates macrophage M2 polarization. **A** Schematic representation of macrophages with TIMM23 knockout using CRISPR/Cas9 gene editing technique (Created with BioRender.com); **B**–**D** Flow cytometry analysis of the expression of CD86 (M1 marker) and CD163 (M2 marker) in different groups of macrophages (**B**, (Created with BioRender.com); **E** Schematic representation of the effect of TIMM23 on mitophagy in M2 macrophages using Western blot, confocal laser microscopy, and transmission electron microscopy techniques; **F** Western blot analysis of the expression levels of mitophagy-related proteins PINK1, p-Parkin/Parkin, and LC3I/II in different groups of M2 macrophages; **G**, **H** Co-localization of mitochondria and LC3 in different groups of M2 macrophages observed using confocal laser microscopy (scale bar: 150 μm); **I** Observation of mitochondrial morphology in cells using transmission electron microscopy, left scale bar: 2 μm, right scale bar: 1 μm, red arrows indicate autophagosomes. Flow cytometry analysis data were analyzed using one-way ANOVA followed by Dunnett’s test, and the results are presented as mean ± SEM (n = 3). * indicates significant difference compared to the TIMM23-WT group (*p* < 0.05), ** indicates significant difference compared to the TIMM23-WT group (*p* < 0.01), # indicates significant difference compared to the oe-NC group (*p* < 0.05), ## indicates significant difference compared to the oe-NC group (*p* < 0.01), cell experiments were repeated three times.

To confirm the above hypothesis, we first induced the differentiation of THP-1 cells into macrophages by adding phorbol-12-myristate-13-acetate (PMA) to the culture medium. TIMM23-KO macrophages were generated using CRISPR/Cas9 gene editing technology (with TIMM23-WT as the wild-type control, Fig. 3A). RT-qPCR and Western blot techniques were employed to determine the expression level of TIMM23 in monoclonal cells, and monoclonal cells with zero expression were selected for subsequent experiments (Fig. S5A, B). Additionally, lentivirus was used to generate macrophages overexpressing TIMM23 (oe-TIMM23, with oe-NC as the control). RT-qPCR and Western blot techniques were used to confirm the transfection efficiency of TIMM23, showing a significant increase in TIMM23 expression after transfection (Fig. S5C, D).

Flow cytometry was utilized to determine the levels of M1 and M2 macrophage phenotypes in each group. The results indicated that compared to the TIMM23-WT group, the TIMM23-KO group showed a significant increase in the M1 macrophage population and a significant decrease in the M2 macrophage population. In contrast, the oe-TIMM23 group demonstrated a significant decrease in M1 macrophage population and a significant increase in M2 macrophage population compared to the oe-NC group (Fig. 3B–D). These results confirmed that TIMM23 indeed promoted the M2 polarization of macrophages while inhibiting their M1 polarization.

Existing literature has reported the crucial role of mitophagy in macrophage polarization, as it can promote M2 polarization [30]. Moreover, TIMM23 plays a unique role in activating mitophagy [31]. The results from Fig. S4 also indicated a potential link between TIMM23 and mitochondrial function. Thus, we further investigated whether TIMM23 could affect mitophagy in M2 macrophages using Western blot, confocal laser microscopy, and transmission electron microscopy techniques (Fig. 3E). Firstly, we examined the expression levels of mitophagy-related proteins PINK1, p-Parkin/Parkin, and LC3II/I in M2 macrophages after TIMM23 knockout or overexpression. The results showed a significant decrease in the expression levels of PINK1, p-Parkin/Parkin, and LC3II/I in TIMM23-KO M2 macrophages compared to the TIMM23-WT group, while the trend was reversed in the oe-TIMM23 group compared to the oe-NC group (Fig. 3F). Confocal laser microscopy observation of the colocalization of mitochondria and LC3 used to monitor changes in mitophagy revealed decreased colocalization after TIMM23 knockout and increased colocalization after TIMM23 overexpression in M2 macrophages (Fig. 3G, H). Electron microscopy observations demonstrated a reduction in the formation of mitophagosomes after TIMM23 knockout, whereas there was an inverse trend with TIMM23 overexpression (Fig. 3I).

Overall, these results indicate that TIMM23 can regulate macrophage M2 polarization through mediating mitophagy.

### Regulation of TIMM23 in M2 macrophages enhances OS cell biological functions

**Fig. 4:**
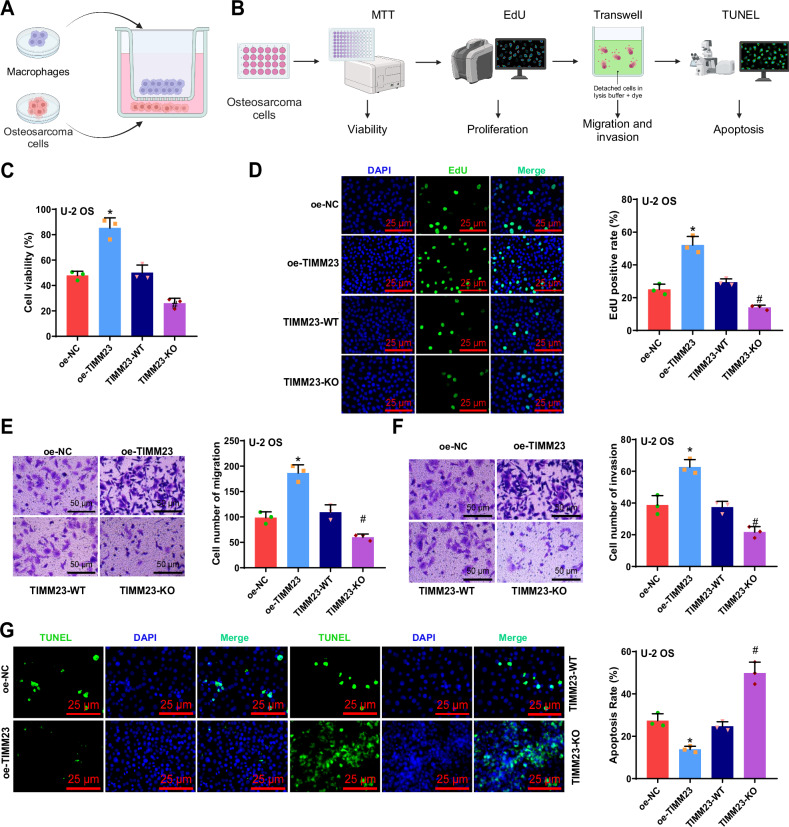
Effect of TIMM23 on the biological functions of U-2 OS cells. **A** Schematic representation of co-culturing of macrophages and OS cells (Created with BioRender.com); **B** Schematic representation of experiments such as MTT assay, EdU assay, Transwell assay, and TUNEL staining to investigate the effect of TIMM23 on the biological functions of OS cells (Created with BioRender.com); **C** MTT assay to assess cell viability in different groups of U-2 OS cells; **D** EdU assay to evaluate cell proliferation ability in different groups of U-2 OS cells (scale bar: 25 μm); **E**, **F** Transwell assay to assess cell migration and invasion ability in different groups of U-2 OS cells (scale bar: 50 μm); **G** TUNEL assay to measure apoptosis rate in different groups of U-2 OS cells (scale bar: 50 μm). * indicates a significant difference compared to the oe-NC group (*p* < 0.05), # indicates a significant difference compared to the TIMM23-WT group (*p* < 0.05), and cell experiments were repeated 3 times.

To further investigate the impact of TIMM23 regulation on macrophage polarization and its effect on OS cell biology, we differentiated THP-1 cells into macrophages and co-cultured them with U-2 OS/MG-63 cells (Fig. 4A). We then assessed the viability of U-2 OS/MG-63 cells in the lower chamber using the MTT assay, measured cell proliferation using the EdU experiment, evaluated cell migration and invasion using the Transwell assay, and determined cell apoptosis using the TUNEL staining (Fig. 4B). The results demonstrated that co-culturing U-2 OS/MG-63 cells with macrophages overexpressing TIMM23 significantly increased cell viability, proliferation, migration, and invasion compared to the oe-NC group while decreasing the level of cell apoptosis. In contrast, co-culturing U-2 OS/MG-63 cells with macrophages lacking TIMM23 significantly reduced cell viability, proliferation, migration, and invasion compared to the TIMM23-WT group while increasing the level of cell apoptosis (Fig. 4C–G, Fig. S6A–E).

These findings indicate that TIMM23 in M2 macrophages can enhance the vitality, proliferation, migration, and invasion of OS cells while simultaneously inhibiting cell apoptosis.

### Giant cell-mediated enhancement of OS cell biology by TIMM23-PARGP1 fusion gene

**Fig. 5:**
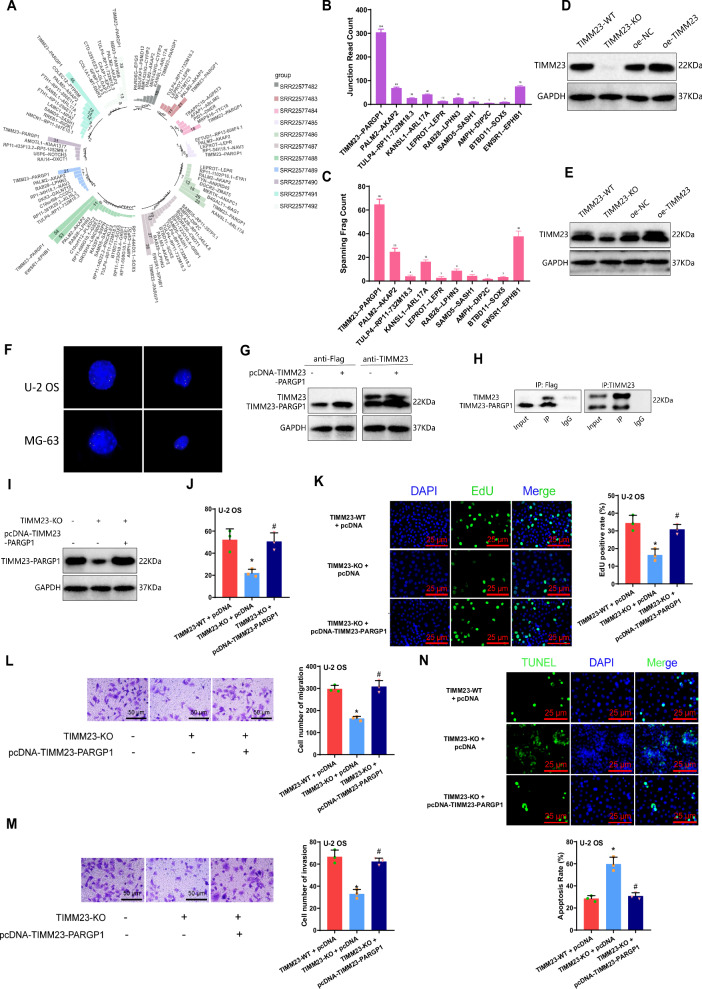
Integration of gene analysis and experimental verification results. **A** Visualization of Junction Read Count for each fusion gene pair in SRR22577482-SRR22577492 data. The Junction Read Count represents the number of specific splice junction reads. Higher splice junction read counts indicate more frequent occurrence of the splice event; **B**, **C** Total statistics of Junction Read Count and Spanning Frag Count for the top 10 ranked fusion gene pairs in SRR22577482-SRR22577492 data. Spanning Frag Count represents the number of fragments spanning a specific splice junction. Higher spanning fragment counts indicate a more common occurrence of the splice event in the sample; **D** Expression of TIMM23 protein in supernatant of co-cultured cells from each group as detected by Western blotting; **E** Expression of TIMM23 protein in U-2 OS cells from each co-culture cell model as detected by Western blotting; **F** Left image shows FISH signals indicating separation of the PARGPA gene (split red and orange signals), right image shows fusion detection using 3-color FISH, with telomere-PARGP1 (5′) fusion shown in red and telomere-TIMM23 (3′) fusion shown in green, confirming the TIMM23-PARGP1 fusion; **G** Expression of TIMM23 and TIMM23-PARGP1 proteins in U-2 OS cells from each co-culture cell model as detected by Western blotting; **H** Co-IP experiment investigating the interaction between TIMM23 and TIMM23-PARGP1; **I** Expression of TIMM23-PARGP1 protein in U-2 OS cells from each co-culture cell model as detected by Western blotting; **J** Viability of U-2 OS cells from each group as measured by MTT assay; **K** Proliferative capacity of U-2 OS cells from each group as evaluated by EdU assay (scale bar: 25 μm); **L**, **M** Migration and invasion abilities of U-2 OS cells from each group as determined by Transwell assay (scale bar: 50 μm); **N** Apoptosis rate of U-2 OS cells from each group as detected by TUNEL assay (Scale bar: 50 μm). * indicates *p* < 0.05 compared to the TIMM23-WT + pcDNA group, # indicates *p* < 0.05 compared to the TIMM23-KO + pcDNA group. Cell experiments were repeated three times.

Gene fusion is a common phenomenon in tumors, which typically promotes cancer occurrence and progression [32]. We performed fusion gene pair analysis on 11 transcriptome sequencing raw data fastq files (SRR22577482-SRR22577492) obtained from the SRA database, using the “STAR” and “STAR-Fusion” packages in Python (Fig. S7). We identified 52 fusion gene pairs, including the TIMM23-PARGP1 fusion gene pair. The fusion and breakpoint details are shown in Fig. S8. Additionally, the Junction Read Count of the TIMM23-PARGP1 fusion gene ranked among the top three in all 11 transcriptome sequencing raw data (Fig. 5A). When we examined the total Junction Read Count and Spanning Frag Count of the top 10 fusion gene pairs in these 11 transcriptome sequencing raw data, TIMM23-PARGP1 ranked first (Fig. 5B, C). These results suggest a close association between TIMM23-PARGP1 and OS. Existing literature indicates a high frequency of the TIMM23-PARGP1 fusion gene in lung cancer, suggesting its functional role in cancer [33].

Through Western blot analysis of the supernatant of co-cultured cells and OS cells, we observed a significant decrease in TIMM23 protein expression in macrophages with TIMM23 knockout. Conversely, there was a significant increase in TIMM23 protein expression in macrophages overexpressing TIMM23. In the detection of TIMM23 protein expression in OS cells, we observed a distinct lower band that showed a trend consistent with TIMM23 expression, which could be TIMM23-PARGP1 (Fig. 5D, E, Fig. S9A, B). FISH results confirmed the presence of TIMM23-PARGP1 gene fusion in U-2 OS/MG-63 cells, demonstrating the existence of TIMM23-PARGP1 (Fig. 5F). Therefore, we hypothesize that TIMM23 may be transferred from M2-polarized macrophages to OS cells after polarization, thereby affecting TIMM23-PARGP1 expression and subsequently impacting the biological functions of OS cells. To test this hypothesis, we constructed an overexpression plasmid for TIMM23-PARGP1 and transfected it into OS cells. We then analyzed protein expression in OS cells before and after overexpression. The experimental results revealed the detection of a smaller protein band, in addition to the endogenous size protein, in OS cells using anti-TIMM23 antibody. This protein band was consistent with the protein detected by the anti-Flag antibody and in OS cells from the co-culture model. Moreover, the expression of this protein significantly increased in the overexpression of the TIMM23-PARGP1 plasmid. These findings indicate the successful transfection of the TIMM23-PARGP1 overexpression plasmid into OS cells (Fig. 5G, Fig. S9C). Furthermore, the Co-IP experiment demonstrated the interaction between TIMM23 and the TIMM23-PARGP1 fusion gene pair (Fig. 5H, Fig. S9D).

Subsequently, we co-cultured OS cells with gene knockdown of TIMM23 and overexpression of TIMM23-PARGP1 fusion gene. Western blot analysis was performed using anti-Flag antibody. The results revealed a significant decrease in the expression of TIMM23-PARGP1 protein in the TIMM23-KO + pcDNA group of OS cells compared to the TIMM23-WT + pcDNA group. Conversely, the TIMM23-KO + pcDNA-TIMM23-PARGP1 group exhibited a significant increase in the expression of TIMM23-PARGP1 protein compared to the TIMM23-KO + pcDNA group (Fig. 5I, Fig. S9E). Further assessment of the cellular functions of OS cells using MTT assay, EdU assay, Transwell assay, and TUNEL staining showed that the viability, proliferation, migration, and invasion abilities were significantly reduced in the TIMM23-KO + pcDNA group compared to the TIMM23-WT + pcDNA group. Additionally, the level of apoptosis in the cells was significantly increased. On the other hand, the TIMM23-KO + pcDNA-TIMM23-PARGP1 group exhibited a significant enhancement in the viability, proliferation, migration, and invasion abilities of OS cells, accompanied by a significant decrease in apoptosis level compared to the TIMM23-KO + pcDNA group (Fig. 5J–N, Fig. S9F–J).

In conclusion, our experimental findings indicate that TIMM23 promotes the production of TIMM23-PARGP1 in OS cells by mediating the M2 polarization of macrophages, thereby affecting the biological functions of OS cells.

### TIMM23 promotes chemoresistance in OS cells through macrophage M2 polarization

Numerous studies have reported a close relationship between fusion genes and chemotherapy resistance in tumors [34–37]. Chemotherapy resistance poses a significant obstacle in cancer treatment, as it significantly reduces treatment efficacy and increases side effects and toxicity [38, 39]. Therefore, investigating the mechanisms underlying chemotherapy resistance has become urgent. Among the existing literature on chemotherapy resistance, inhibiting tumor cell activity and apoptosis is the most commonly observed mechanism [40]. Hence, we selected commonly used chemotherapy drugs, Cis, doxorubicin (Dox), and methotrexate (Mtx), for evaluating the impact of TIMM23-PARGP1 fusion gene expression levels on chemoresistance in OS cells [41, 42].

**Fig. 6:**
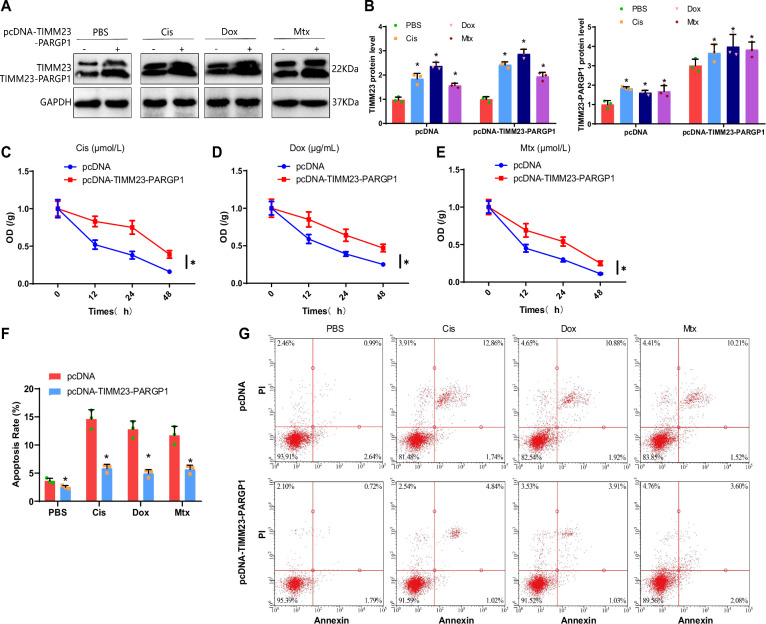
Influence of TIMM23-PARGP1 on chemoresistance of U-2 OS cells. **A**, **B** Expression of TIMM23 and TIMM23-PARGP1 proteins in U-2 OS cells from each group as detected by Western blotting; **C**–**E** Viability of U-2 OS cells from each group under chemotherapy drug treatment as determined by CCK-8 assay; **F**, **G** Apoptosis of U-2 OS cells from each group under chemotherapy drug treatment as measured by flow cytometry. * indicates *p* < 0.05 compared to the pcDNA group or PBS group. Cell experiments were repeated three times.

First, we used anti-TIMM23 antibodies to detect the expression levels of TIMM23-PARGP1 protein through Western blot. The results demonstrated a significant increase in the expression of TIMM23 and TIMM23-PARGP1 in U-2 OS/MG-63 cells after treatment with Cis, Dox, or Mtx alone (Fig. 6A, B, Fig. S10A, B). Subsequently, we assessed the cell viability and apoptosis levels in OS cells overexpressing TIMM23-PARGP1. The results showed a significant reduction in the response of U-2 OS/MG-63 cells to Cis, Dox, and Mtx after TIMM23-PARGP1 overexpression, indicated by weakened inhibition of cell viability and decreased apoptosis (Fig. 6C–G, Fig. S10C–G). These findings suggest that TIMM23-PARGP1 is upregulated during the chemotherapeutic process in OS cells and that high expression of TIMM23-PARGP1 hinders the cytotoxic effects of chemotherapy drugs on these cells.

**Fig. 7:**
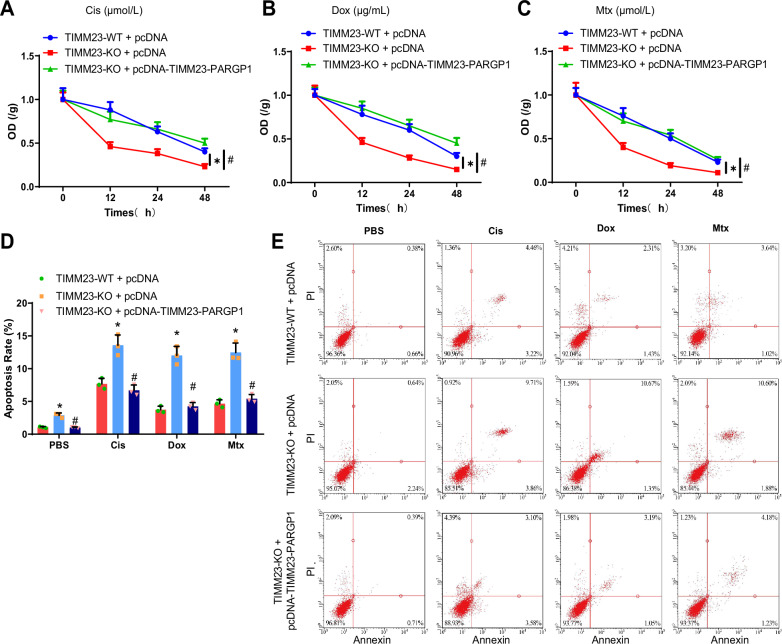
The effect of TIMM23 and TIMM23-PARGP1 on chemoresistance in U-2 OS cells. **A**–**C** Viability of different groups of U-2 OS cells treated with chemotherapy drugs evaluated using the CCK-8 assay; **D**, **E** Apoptosis of different groups of U-2 OS cells treated with chemotherapy drugs evaluated using flow cytometry. * indicates statistically significant differences compared to the TIMM23-WT + pcDNA group (*p* < 0.05), # indicates statistically significant differences compared to the TIMM23-KO + pcDNA group (*p* < 0.05). All cellular experiments were performed three times.

Next, we co-cultured macrophages and U-2 OS/MG-63 cells, treating the latter with Cis, Dox, or Mtx, to investigate the role of TIMM23 in mediating M2 polarization of macrophages and its impact on chemoresistance in OS cells. Cell Counting Kit-8 (CCK-8) and flow cytometry results indicated that compared to the TIMM23-WT + pcDNA group, the inhibition of OS cell viability and the induction of apoptosis were enhanced in the TIMM23-KO + pcDNA group, suggesting significantly improved treatment efficacy. In contrast, the TIMM23-KO + pcDNA-TIMM23-PARGP1 group exhibited weakened inhibition of OS cell viability and decreased apoptosis compared to the TIMM23-KO + pcDNA group, implying a significant increase in chemoresistance and a reduction in treatment efficacy (Fig. 7A–E, Fig. S11A–E).

In summary, TIMM23 promotes the production of TIMM23-PARGP1 in OS cells through mediating macrophage M2 polarization, thus influencing the chemoresistance of these cells.

### Macrophage-mediated M2 polarization promotes chemoresistance and tumor formation in OS cells

In vitro cell experiments confirmed that TIMM23 mediates M2 polarization of macrophages, thereby promoting the expression of TIMM23-PARGP1 in OS cells and influencing chemoresistance. To validate whether this mechanism affects the in vivo tumorigenic capability of OS cells, we constructed OS cells overexpressing TIMM23-PARGP1-FLAG through lentiviral transduction and verified the transduction efficiency by Western blotting, which showed a significant increase in TIMM23-PARGP1-FLAG expression after transduction (Fig. S12A). Subsequently, we co-cultured TIMM23 knockout macrophages with OS cells overexpressing TIMM23-PARGP1 in vitro and, after 48 h, injected the OS cells from the culture medium into the subcutaneous region of mice to establish a mouse model of OS xenograft. From day 7, mice were treated with either PBS solution or 5 mg/kg of Dox through intraperitoneal injections.

**Fig. 8:**
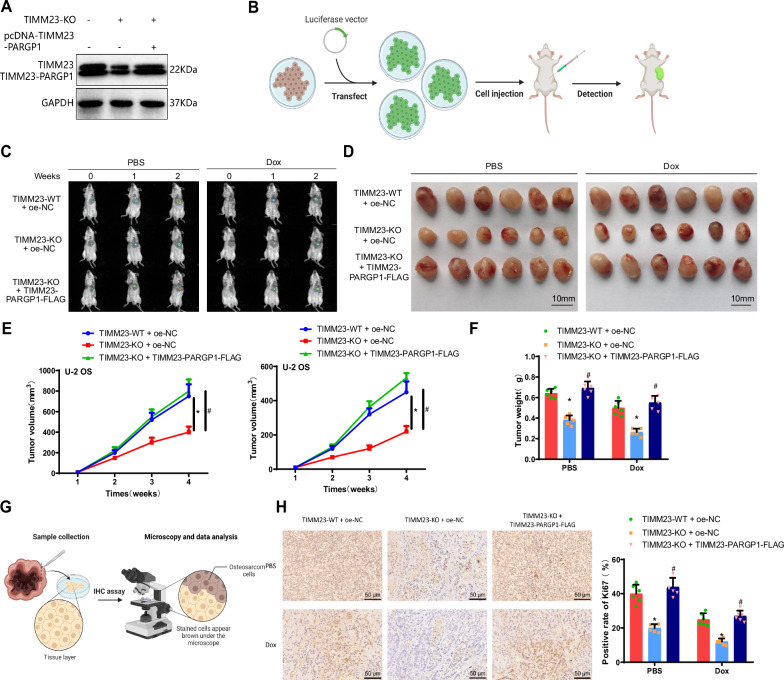
The impact of TIMM23-mediated M2 polarization of macrophages on the generation of TIMM23-PARGP1 in U-2 OS cells and tumorigenesis in vivo. **A** Protein expression levels of TIMM23 and TIMM23-PARGP1 in mouse tumor tissues detected using Western blot; **B**, **C** Tumor growth monitored at different time points using bioluminescence intensity, with one example representative for each group (**B**, Created with BioRender.com); **D** Morphology of tumor tissues in each group of mice; **E** Tumor growth in each group of mice; **F** Tumor tissue weights in each group of mice; **G**, **H** Protein expression levels of Ki67 in mouse tumor tissues detected using immunohistochemical staining (scale bar: 50 μm). * indicates statistically significant differences compared to the TIMM23-WT + oe-NC group (*p* < 0.05), # indicates statistically significant differences compared to the TIMM23-KO + oe-NC group (*p* < 0.05). Each group consisted of six mice (G, Created with BioRender.com).

First, Western blotting analysis revealed that compared to the TIMM23-WT + oe-NC + Dox group, the TIMM23-KO + oe-NC + Dox group exhibited significantly decreased expression of both TIMM23 and TIMM23-PARGP1 in tumor tissues. Compared to the TIMM23-KO + oe-NC + Dox group, the TIMM23-KO + TIMM23-PARGP1-FLAG + Dox group showed no significant change in TIMM23 expression but a significant increase in TIMM23-PARGP1 expression in tumor tissues (Fig. 8A, Fig. S12B).

After 1 and 2 weeks, bioluminescent imaging was performed to observe tumor growth in mice. The results demonstrated that compared to the TIMM23-WT + oe-NC/TIMM23-WT + oe-NC + Dox groups, the TIMM23-KO + oe-NC/TIMM23-KO + oe-NC + Dox groups showed inhibited tumor growth in tissues. On the other hand, compared to the TIMM23-KO + oe-NC/TIMM23-KO + oe-NC + Dox groups, the TIMM23-KO + TIMM23-PARGP1-FLAG/TIMM23-KO + TIMM23-PARGP1-FLAG + Dox groups exhibited accelerated tumor growth in tissues. Furthermore, the tumor tissues in the Dox group were significantly smaller than those in the PBS group (Fig. 8B, C, Fig. S12C). Comparisons of tumor volume and weight among the groups showed that the TIMM23-KO + oe-NC/TIMM23-KO + oe-NC + Dox groups had significantly reduced tumor volume and weight compared to the TIMM23-WT + oe-NC/TIMM23-WT + oe-NC + Dox groups. In contrast, the TIMM23-KO + TIMM23-PARGP1-FLAG/TIMM23-KO + TIMM23-PARGP1-FLAG + Dox groups exhibited significantly increased tumor volume and weight compared to the TIMM23-KO + oe-NC/TIMM23-KO + oe-NC + Dox groups. Moreover, the tumor tissues in the Dox group were noticeably smaller in volume and weight than those in the PBS group (Fig. 8D–F, Fig. S12D-F).

Furthermore, immunohistochemical staining using Ki67 was performed on the mouse tumor tissues. The results showed that compared to the TIMM23-WT + oe-NC/TIMM23-WT + oe-NC + Dox groups, the TIMM23-KO + oe-NC/TIMM23-KO + oe-NC + Dox groups exhibited a significantly lower percentage of Ki67-positive cells in tumor tissues. In comparison, the TIMM23-KO + TIMM23-PARGP1-FLAG/TIMM23-KO + TIMM23-PARGP1-FLAG + Dox groups showed a significantly higher percentage of Ki67-positive cells in tumor tissues. Moreover, the tumor tissues in the Dox group had noticeably lower levels of Ki67-positive cells compared to the PBS group (Fig. 8G, H, Fig. S12G). These results suggest that TIMM23 mediates M2 polarization of macrophages, promotes the production of TIMM23-PARGP1 in OS cells, influences chemoresistance, and facilitates in vivo tumor formation in OS cells.

## Discussion

In recent years, single-cell transcriptomics has been extensively applied in cancer research due to its high resolution in delineating cellular heterogeneity and identifying rare cell populations, thereby offering new perspectives for studying the tumor immune microenvironment [43–45]. Compared with conventional transcriptomic approaches, single-cell-level analysis is more advantageous in uncovering expression profiles of specific genes and fusion events involved in macrophage polarization—particularly the key regulators functioning in M2-related pathways [46–48]. In this study, through integrative single-cell transcriptomic analysis, we identified a strong intercellular interaction between macrophages and OS cells and screened the fusion gene TIMM23-PARGP1, which is closely associated with M2 macrophage polarization. Compared with previous studies, we further elucidated the role of macrophages in the OS immune microenvironment and their contribution to chemoresistance, offering new directions for dissecting the underlying regulatory mechanisms.

The key gene TIMM23-PARGP1, identified via WGCNA co-expression network analysis and the STAR-Fusion tool, was found to be tightly associated with chemoresistance in OS [49–51]. Furthermore, the presence and expression of the TIMM23-PARGP1 fusion gene in OS were validated through fluorescence in situ hybridization (FISH) [52, 53].

Our in vitro experiments demonstrated that TIMM23 promotes macrophage M2 polarization by modulating mitophagy, further highlighting its role in immune cell functional regulation and emphasizing the importance of mitophagy in macrophage polarization [54, 55]. Unlike prior studies, we established a direct link between TIMM23 and macrophage polarization, extending its functional relevance in the immune microenvironment. While TIMM23 is known as a core component of the mitochondrial protein translocation complex and partially implicated in mitochondrial homeostasis, its precise role in activating mitophagy remains undefined. Based on literature, intracellular ROS elevation, mitochondrial dysfunction, and AMPK pathway activation can upregulate PINK1/Parkin-mediated autophagy. We hypothesize that TIMM23 may modulate ROS or energy-sensing signals, facilitating mitochondrial damage recognition and initiation of mitophagy, thereby promoting M2 macrophage polarization [27, 30].

Further analysis revealed that TIMM23-mediated M2 polarization facilitates the expression of the TIMM23-PARGP1 fusion gene in OS cells and enhances their proliferation, migration, and invasion capacities while reducing their sensitivity to chemotherapy. This suggests that TIMM23 may reshape the immune microenvironment to provide a more supportive niche for tumor cells to acquire a resistant phenotype. Previous studies have shown that tumor-associated macrophages can secrete inflammatory cytokines such as TNF-α and IL-6, activating the STAT3 signaling pathway and thereby promoting chemotherapy resistance in OS cells [25]. Based on this, we speculate that under TIMM23-driven M2 polarization, TIMM23-PARGP1 expression in tumor cells may enhance their adaptability to immune signaling and chemotherapeutic stress, exacerbating resistance.

It is worth noting that the resistance mechanisms of commonly used chemotherapeutic agents such as Mtx, Dox, and Cis are known to be associated with upregulation of DHFR, reduced Topo II activity, and enhanced ERCC1-mediated DNA repair, respectively [56–58]. Although the specific function of TIMM23-PARGP1 remains unclear, as a mitochondria-related fusion gene, it may act synergistically in these pathways by maintaining mitochondrial homeostasis, inhibiting chemotherapy-induced apoptosis, or regulating responses to oxidative stress and DNA damage. These findings offer a new direction for further mechanistic exploration of TIMM23-PARGP1 in OS chemoresistance.

Our results were further validated by in vivo animal experiments. TIMM23-mediated macrophage M2 polarization and the production of TIMM23-PARGP1 play an important role in the tumorigenic process of OS in vivo. Compared to previous studies, our research further clarifies the importance of macrophage M2 polarization in OS [28, 59, 60].

Based on the above results, we can preliminarily draw the following conclusions: TIMM23 is involved in mitochondrial autophagy-mediated M2 polarization of macrophages, promoting the formation of OS cells containing the TIMM23-PARGP1 fusion gene, thereby influencing chemotherapy resistance and promoting tumor growth in OS cells. Our study provides a theoretical basis for understanding the development of OS and identifying new targets for OS therapy. However, the mechanism by which the regulatory network involving TIMM23 is transmitted to OS cells remains unclear. Thus, further exploration in this direction is warranted.

This study holds significant scientific and clinical value. Firstly, through methods including single-cell transcriptome analysis, WGCNA co-expression network analysis, and fusion gene screening, we have revealed the molecular mechanisms of TIMM23’s involvement in mitochondrial autophagy-mediated M2 polarization of macrophages. This contributes to a deeper understanding of the functions and regulatory mechanisms of macrophages in the TME, providing a theoretical basis for developing new tumor treatment strategies. Secondly, this study has discovered the influence of TIMM23 on chemotherapy resistance in OS cells. Chemotherapy resistance is a common problem in clinical treatment and limits the effectiveness of tumor therapy. Understanding the mechanism of TIMM23 in chemotherapy resistance will offer new insights for finding strategies to reverse resistance and improve chemotherapy outcomes.

However, this study also has some limitations. Firstly, our research mainly relied on in vitro cell experiments and mouse models, lacking validation from clinical samples. Therefore, further clinical research is needed to verify the reliability and accuracy of our findings in pathological conditions in humans. Secondly, this study focused only on the TIMM23-PARGP1 fusion gene and mitochondrial autophagy in OS without exploring their roles in other types of tumors. Thus, further research is required to expand the scope and potential mechanisms of these findings.

In terms of future prospects, further research can explore the roles of TIMM23 and mitochondrial autophagy in other types of tumors and deepen the understanding of their biological functions and clinical applications. Additionally, the potential roles of TIMM23 in tumor immunotherapy can be further investigated, exploring its potential as a targeted therapy. Furthermore, we also need to explore more resistance mechanisms and reversal strategies to improve the effectiveness of chemotherapy and reduce the failure rate caused by chemotherapy resistance.

## Materials and methods

### Acquisition of OS-related single-cell transcriptomic data

To obtain scRNA-seq data related to OS, we accessed the Gene Expression Omnibus (GEO) database and retrieved the datasets GSE162454 and GSE126209. Dataset GSE162454 consists of primary OS tissue samples from six different patients. The data was analyzed using the “Seurat” package in the R software [61]. Data quality control was performed by filtering for features with nFeature_RNA between 200 and 5000 and the percentage of mitochondrial genes (percent.mt) below 25. We further selected the top 2000 highly variable genes based on variance [62]. Dataset GSE126209 contains samples from six OS tumor tissues and five adjacent normal tissues. Gene expression data from GSE126209 was subjected to normalization and analyzed using the “limma” package in the R language to study gene expression patterns in OS.

For the identification and analysis of fusion genes associated with OS, we retrieved the original transcriptomic sequencing data in fastq format (SRR22577482-SRR22577492) from the Sequence Read Archive (SRA). We conducted fusion gene detection and analysis on the SRR22577482-SRR22577492 data using the “STAR” and “STAR-Fusion” packages in Python. The results were visualized using the “FusionInspector” package in Python, the “chimeraviz” package in R, and the IGV software (2.16.2) [63–65].

### UMAP clustering analysis and cell annotation

To reduce the dimensionality of scRNA-Seq datasets and facilitate downstream analysis, we employed Principal Component Analysis (PCA) based on the top 2000 highly variable genes. We selected the first 30 principal components for further analysis using the Elbowplot function in the Seurat software package. Subsequently, the FindClusters function in Seurat was used with a resolution parameter set at 0.4 to identify major cell subgroups. Non-linear dimensionality reduction of scRNA-seq sequencing data was then performed using the UMAP algorithm. Cell annotation was carried out by leveraging known cell lineage-specific marker genes and combining them with the online resource CellMarker. Detailed lists of annotated marker genes for each cell type can be found in Table S1 and Table S2 [66].

### Identification of DEGs and WGCNA

Using the “limma” package in R language, we employed the criteria of |log2FC| > 2 and *p* < 0.05 to identify DEGs and to investigate the variation between different subtypes of macrophages within OS samples. Additionally, DEGs within the OS samples were also screened using the criterion of *p* < 0.05. To visualize the expression patterns of DEGs, we utilized the “pheatmap” package in R software to generate heatmaps and volcano plots [67].

Furthermore, the “WGCNA” package in R language was utilized to perform expression-based clustering analysis and phenotype association analysis on the GSE126209 dataset. Through this analysis, we identified crucial gene modules associated with OS and presented the visualization of their module importance [68].

### Construction and cultivation of in vitro cell models

The human OS cell lines U-2 OS (HTB-96) and MG-63 (CRL-1427), as well as the human monocytic cell line THP-1 (TIB-202), were obtained from the American Type Culture Collection (ATCC). U-2 OS and MG-63 cells were cultured in McCoy’s 5 A medium (16600082, Gibco, USA) supplemented with 10% fetal bovine serum (FBS), 10 μg/mL streptomycin, and 100 U/mL penicillin. THP-1 cells were cultured in RPMI-1640 medium (A1049101, Gibco, USA) supplemented with 10% FBS and 1% penicillin/streptomycin. Once the density of THP-1 monocytes reached 1.0 × 10^5^ cells/cm^2^, 50 nM phorbol myristate acetate (PMA; HY-18739, MedChemExpress, USA) was added, and the cells were further incubated for 48 h in a cell culture incubator (0.4 μm pore size; CLS3412, Corning, USA) to induce their differentiation into macrophages [40, 69].

The 293 T cell line was obtained from the American Type Culture Collection (ATCC, CRL-3216). The 293 T cells were cultured in Dulbecco’s Modified Eagle Medium (DMEM, 11965092, Gibco, USA) supplemented with 10% FBS, 10 μg/mL streptomycin, and 100 U/mL penicillin. All the cell types mentioned above were cultured in a humidified incubator (Heracell™ Vios 160i CR CO_2_ Incubator, 51033770, Thermo Scientific™, Germany) at 37 °C with 5% CO_2_. When the cells reached 80–90% confluence, passage culturing was performed [70].

### Construct and transfect lentivirus

Lentiviral vectors overexpressing TIMM23 and TIMM23-PARGP1-FLAG were constructed and packaged by Genechem (Shanghai, China). The lentiviral overexpression vector used was LV-PDGFRA. Additionally, for subsequent bioluminescence imaging experiments, the overexpression vector contained the GFP/mCherry genes. The packaged virus and the target vector were co-transfected into 293T cells (at a confluence of 80–90%) using Lipofectamine 2000 (11668500, Invitrogen, USA). After 48 h of cell culture, the supernatant was collected, and the supernatant containing virus particles was filtered by centrifugation. The virus from exponentially growing cells was collected for virus titer detection. When macrophages/OS cells reached the logarithmic growth phase, they were dissociated using trypsin and trituration, and a cell suspension of 5 × 10^4^ cells/mL was seeded in a 6-well plate, with 2 mL per well. Prior to constructing the in vitro cell model, lentiviral vectors overexpressing TIMM23 or TIMM23-PARGP1-FLAG (MOI = 10, virus titer: 1 × 10^8^ TU/mL) were separately added to the culture medium of macrophages or OS cells and incubated for 48 h. Stable cell clones were selected using 2 µg/mL puromycin (UC0E03, Sigma-Aldrich, USA) for a duration of 2 weeks [71–74].

### Construction of TIMM23-KO cells using CRISPR/Cas9 technology

In this study, we utilized the CRISPR/Cas9 technique to generate THP-1 cells with the TIMM23 gene knockout. Two specific guide RNAs (sgRNAs) were employed: TIMM23-sgRNA1: 5′-GCCGGCTTTTTCGGAGCCGG-3′ (PAM: CGG) and TIMM23-sgRNA2: 5′-TGCATGACAGGGGCTGCGTT-3′ (PAM: TGG). These sgRNAs were inserted into the Lenti-CRISPR v2 vector containing the Streptococcus pyogenes Cas9 nuclease gene (HanHeng Biotechnology (Shanghai) Co., Ltd., Shanghai). The cells were then transduced with the lentiviral Lenti-CRISPR v2 vector and subjected to the CRISPR/Cas9 editing system to generate TIMM23 gene knockout cells. To select the cells with sgRNA plasmid transfection and donor sequence, a screening process was performed using 4 μg/mL puromycin (A1113803, Gibco, USA). Surviving cells were obtained through limited dilution cloning, and TIMM23 gene knockout cells were further confirmed by RT-qPCR and Western blotting. Finally, DNA sequencing was conducted to validate the presence of TIMM23 gene knockout [75, 76].

### Construction of a fusion gene

Specific primers were designed to amplify the corresponding regions of the TIMM23 and PARGP1 genes. The PCR products were purified, and the TIMM23 and PARGP1 fragments were digested with restriction enzymes. The digested TIMM23 and PARGP1 fragments were ligated together to form the TIMM23-PARGP1 fusion gene. The fusion gene was then inserted into the mammalian expression vector pcDNA3.1-Flag-C to create the recombinant expression vector. Finally, Escherichia coli transformation and selection of resistant clones were performed, followed by sequencing verification to confirm the correct expression vector of the TIMM23-PARGP1 fusion gene [77].

### Cell transfection and grouping

U-2 OS/MG-63 cells were seeded at a density of 3 × 10^5^ cells per well in a 6-well plate and cultured until the cell confluency reached 70–80%. Then, the cells were transfected using Lipofectamine 3000 reagent (L3000015, Invitrogen, USA). The cells were divided into two groups: (1) pcDNA group, transfected with the empty vector pcDNA3.1-Flag-C; (2) pcDNA-TIMM23-PARGP1 group, transfected with the pcDNA3.1-TIMM23-PARGP1-Flag-C plasmid. At 36 and 48 h, RNA and protein levels were examined to verify the overexpression efficiency. The above-mentioned plasmids were designed and synthesized by Guangzhou Rebo Biotechnology Co., Ltd.

### Cell co-culture

In the cell co-culture experiment, an in-vitro macrophage model was seeded onto the upper compartment of a Transwell co-culture plate (CLS3412, Corning, USA) and placed on an empty 6-well plate. After pre-treatment according to the respective groups, the original culture medium was discarded, and the cells were gently washed twice with PBS. Subsequently, the cells were transferred to a 6-well plate seeded with OS cells U-2 OS/MG-63, with a ratio of 2:1 for macrophages to U-2 OS/MG-63 cells. The co-culture was continued for 36 h, after which the supernatant and the OS cells in the lower compartment were collected for further experiments [78–81]. For the chemotherapy resistance experiment, the culture medium was supplemented with 20 μmol/L Cis (HY-17394, MedChemExpress, USA), 0.2 μg/mL Dox (HY-15142A, MedChemExpress, USA), and 50 μmol/L methotrexate (Mtx; HY-14519, MedChemExpress, USA). CCK-8 assay was performed at 0, 12, 24, and 48 h, and flow cytometry was done at 48 h for subsequent analysis. The control group was injected with an equal amount of PBS solution [40].

The cell co-culture groups were as follows: (1) TIMM23-WT group: co-culture of wild-type macrophages with U-2 OS/MG-63 cells; (2) TIMM23-KO group: co-culture of TIMM23 knockout macrophages with U-2 OS/MG-63 cells; (3) oe-NC group: co-culture of macrophages transfected with empty vector and U-2 OS/MG-63 cells; (4) oe-TIMM23 group: co-culture of macrophages transfected with TIMM23 overexpression vector and U-2 OS/MG-63 cells; (5) TIMM23-WT + pcDNA group: co-culture of TIMM23 knockout macrophages with U-2 OS/MG-63 cells transfected with pcDNA3.1-Flag-C plasmid; (6) TIMM23-KO + pcDNA group: co-culture of macrophages transfected with empty vector and U-2 OS/MG-63 cells transfected with pcDNA3.1-Flag-C plasmid; (7) TIMM23-KO + pcDNA-TIMM23-PARGP1 group: co-culture of macrophages transfected with TIMM23 overexpression vector and U-2 OS/MG-63 cells transfected with pcDNA3.1-TIMM23-PARGP1-Flag-C plasmid.

### Co-immunoprecipitation (Co-IP)

U-2 OS or MG-63 cells were seeded into 6 cm plates and treated with 10 μM proteasome inhibitor MG132 (HY-13259, Med Chem Express, USA) for 6 h. Cells were then collected and lysed using NP-40 lysis buffer (P0013F, Beyotime Biotechnology Co., Ltd, Shanghai, China). A total of 40 μg of protein was prepared as the input group, and the remaining protein was prepared at a concentration of 1 mg/mL and a volume of 1 mL, which was added to three tubes. Anti-TIMM23 antibody (10 μg; sc-514463, Santa Cruz Biotechnology, USA) or anti-Flag antibody (ab205606, Abcam, UK) or Goat anti-Rabbit IgG antibody (10 μg; 31460, Invitrogen, USA) or Goat anti-Mouse IgG antibody (10 μg; 31460, Invitrogen, USA) was added to the total protein, followed by overnight gentle agitation at 4 °C. Protein A/G agarose (sc-2003, Santa Cruz Biotechnology, USA) was added and incubated for 4 h at 4 °C, followed by three washes with pre-chilled TBS solution. The immunoprecipitated proteins were detected by Western blot analysis [82, 83].

### FISH

FISH experiments were conducted on interphase cell nuclei on 4-μm sections embedded in paraffin. Custom bacterial artificial chromosome (BAC) probes were used, covering and adjacent to genes, including TIMM23 and PARGP1. Following the manufacturer’s instructions, DNA was isolated from a single BAC, labeled with different fluorescent dyes through core labeling reactions, denatured, and hybridized onto pre-treated slides. The slides were then incubated, washed, and fixed using DAPI (D1306, Invitrogen, USA) and antifade solution (S7113, Sigma-Aldrich, Germany). Each BAC set’s genomic position was validated by hybridizing them to normal metaphase chromosomes. Using a Zeiss fluorescence microscope (Zeiss Axioplan, Oberkochen, Germany) and Isis 5 software (Metasystems, Newton, USA), 200 consecutive cell nuclei were examined. A positivity fraction was interpreted when at least 20% of cell nuclei showed separate signals. Cell nuclei with incomplete signals were excluded from the fraction. In cases where both TIMM23 and PARGP1 probes yielded positive results, additional fusion experiments were performed to confirm the co-localization signal. All probes used were synthesized and provided by Zhongding Biologicals (China) [84].

### Flow cytometry

Flow cytometry was utilized to determine the macrophage content. Initially, cells were centrifuged at 1200 × *g* for 5 min at 4 °C, followed by resuspension in staining buffer and collection of macrophages differentiated from THP-1 cells, which were grown in a co-culture model. Single-cell suspensions of macrophages or tumor tissue were subjected to dark incubation at 4 °C for 30 min, and cell dissociation was performed using StemPro™ Accutase™ (A1110501, Gibco, USA). Subsequently, centrifugation at 1000 × *g* for 5 min was conducted, followed by two washes of the cell pellet with PBS. The cells were then suspended in 100 μL of PBS and stained with APC-conjugated anti-CD86 (17-0869-42, Thermo Fisher, USA) and FITC-conjugated anti-CD163 (MA5-17719, Thermo Fisher, USA). After antibody incubation, the cells were washed three times with PBS and immediately subjected to analysis using a flow cytometer (Beckman, USA) [85].

### Co-localization immunofluorescence microscopy

THP-1 cells were differentiated into macrophages and then stained with 400 nM MitoTracker Deep Red (M22426, Invitrogen, USA) for 30 min at 37 °C following pre-treatment. After staining, the cells were collected in a 1.5 mL centrifuge tube and fixed with 4% paraformaldehyde (60536ES60, Yeasen Biotechnology (Shanghai) Co., Ltd., China). Permeabilization was performed with 0.2% Triton X-100 (P0096, Beyotime Biotechnology Co., Ltd., Shanghai, China), followed by blocking with PBS containing 1% BSA. Furthermore, the cells were incubated overnight with a diluted antibody against LC3 (PA1-46286, Invitrogen, USA) at a 1:100 ratio in a blocking buffer. After washing, the cells were incubated with Alexa Fluor™ 488-conjugated goat anti-rabbit antibody (1:100, A-11008, Invitrogen, USA) at a 1:100 ratio in the blocking buffer. Subsequently, the cells were washed again, and nuclear staining was performed with 300 nM 4’,6-diamidino-2-phenylindole (DAPI) (D1306, Invitrogen, USA) in PBS. Finally, the cells were centrifuged at 800 × *g* for 5 min, mounted using ProLong™ Gold Anti-fade Mountant (P36930, Invitrogen, USA), and covered with a coverslip. Macrophages in a 12-well plate were stained following the same procedure after MitoTracker staining, and imaging was conducted using an LSM 700 microscope with a 63×/1.4 NA objective lens (Carl Zeiss Microscopy, Germany) [30].

### TEM

The samples of cells and tumor tissue were prepared for observation under Transmission Electron Microscopy (TEM). Firstly, the samples were fixed overnight in a 2.5% glutaraldehyde solution at 4 °C. Then, the samples were fixed for 1–2 h in a 1% osmium tetroxide solution. Dehydration of the samples was carried out at room temperature using graded ethanols of 50%, 70%, 80%, 90%, and 95%, followed by treatment with pure acetone. Subsequently, the samples were infiltrated overnight with pure embedding resin and then heated at 70 °C overnight to complete the embedding process. The samples were cut into 70–90 nm thick sections using a Reichert ultramicrotome. These sections were stained with lead citrate and uranyl acetate in 50% ethanol-saturated solution for 15 min each. Finally, the sections were observed using a TEM for further analysis [86].

### RT-qPCR

Total RNA was extracted from the tissues or cells using Trizol reagent (15596026, Invitrogen, USA). The concentration and purity of the total RNA were measured at 260/280 nm using NanoDrop LITE (ND-LITE-PR, Thermo Scientific™, Germany). The cDNA of the extracted total RNA was synthesized using the PrimeScript RT reagent Kit with gDNA Eraser (RR047Q, TaKaRa, Japan). Subsequently, RT-qPCR analysis was performed on the ABI PRISM 7500 Sequence Detection System (Applied Biosystems) using SYBR Green PCR Master Mix reagents (4364344, Applied Biosystems, USA) for each gene. The primers for each gene were synthesized by TaKaRa (Table S3), with GAPDH used as the reference gene. The relative expression levels of each gene were analyzed using the 2^-ΔΔCt^ method, where ΔΔCt = (mean Ct value of the target gene in the experimental group − mean Ct value of the reference gene in the experimental group) − (mean Ct value of the target gene in the control group − mean Ct value of the reference gene in the control group) [87–89]. All RT-qPCR experiments were performed in triplicate.

### Western blot

First, enhanced RIPA lysis buffer containing protease inhibitors (P0013B, Beyotime Biotechnology Co., Ltd, Shanghai, China) was used to collect and lyse tissues or cells. Subsequently, the protein concentration was determined using the BCA protein quantification kit (P0012, Beyotime Biotechnology Co., Ltd, Shanghai, China). After protein separation, the proteins were transferred onto a PVDF membrane using 10% SDS-PAGE, and non-specific binding was blocked by incubating the membrane with 5% BSA at room temperature for 2 h. Next, the corresponding diluted primary antibodies (except anti-p-Parkin, which was a monoclonal mouse antibody against humans; others were polyclonal rabbit antibodies against humans, as detailed in Table S4) were added to the samples and incubated at room temperature for 1 h. After washing the membrane, HRP-conjugated goat anti-rabbit secondary antibody (ab6721, 1:2000, Abcam, UK) or goat anti-mouse secondary antibody (ab6785, 1:1000, Abcam, UK) was added and incubated at room temperature for 1 h. Then, equal amounts of A and B solutions from Pierce™ ECL Western Blotting Substrate (32209, Thermo Scientific™, Germany) were mixed in the dark, dropped onto the membrane, and exposed in a gel imaging system. Lastly, photographs were taken using the Bio-Rad image analysis system (BIO-RAD, USA), and Image J software was used for grayscale quantification of the bands in the Western blot images, with GAPDH [88] serving as a reference. Each experiment was performed in triplicate. Full and uncropped western blots are provided in the Supplemental Material.

### MTT and CCK-8 assays

MTT assay: The study employed a density of 3–5 × 10^4^ cells/mL to inoculate the cells in a 96-well cell culture plate, which was then incubated for 48 h. Following this, a 10 mg/mL MTT solution (ST316, Beyotime Biotechnology Co., Ltd, Shanghai, China) was added to the cell suspension and incubated for 4 h. The suspension was then mixed with dimethyl sulfoxide (DMSO) and shaken for 10 min. The absorbance (OD 490 nm) was measured using a spectrophotometer (Laspec, China). Cell viability was calculated using the formula: Cell viability = (ΔA_sample–ΔA_blank)/ΔA_control. Here, ΔA_sample represents the absorbance difference of the sample, ΔA_blank represents the absorbance difference of the blank, and ΔA_control represents the absorbance difference of the control group [90].

CCK-8 assay: After digestion, cells from each group were resuspended and adjusted to a concentration of 1 × 10^5^ cells/mL and seeded in a 96-well plate at a volume of 100 μL per well for standard cultivation. Once the cells adhered to the wall, the treatment drugs were added and incubated overnight. At 0, 12, 24, and 48 h after cultivation, the cells were processed according to the instructions of the CCK-8 kit (C0041, Beyotime, Shanghai) to measure cell viability. For each measurement, 10 μL of CCK-8 detection solution was added and incubated in a culture incubator for 4 h. Subsequently, the absorbance was measured at 450 nm using an enzyme-linking instrument, and cell viability was calculated. The formula for cell viability was: Cell viability = (ΔA_sample–ΔA_blank)/ΔA_control. As before, ΔA_sample represents the absorbance difference of the sample, ΔA_blank represents the absorbance difference of the blank, and ΔA_control represents the absorbance difference of the control group [40, 91].

### EdU experiment

The test cells were seeded in a 24-well plate. EdU (C10337, Invitrogen, USA) was added to the culture medium to achieve a concentration of 10 µmol/L. Following a 2-h incubation in the cell culture incubator, the culture medium was removed. Then, the cells were fixed in a PBS solution containing 4% paraformaldehyde at room temperature for 15 min. The cells were washed twice with PBS containing 3% BSA. Subsequently, 0.5% Triton-100 in PBS was added to the cells and incubated at room temperature for 20 min. The cells were washed again twice with PBS containing 3% BSA. A 100 µL staining solution was added to each well and incubated at room temperature in the dark for 30 min. After adding DAPI for nuclear staining, the samples were coverslipped. Randomly, 6-10 fields of view were observed under a fluorescence microscope (BX63, Olympus, Japan), and the number of positive cells in each field was recorded. The EdU labeling rate (%) was calculated as the number of positive cells divided by the total number of cells (positive cells + negative cells) multiplied by 100% [92]. Each experiment was repeated three times.

### Transwell experiment

ECM gel (E1270, Sigma-Aldrich, Germany) was added to the upper chamber of a 24-well Transwell plate (8 μm) and then incubated in a 37 °C incubator for 30 min for gel solidification. After 48 h of transfection, cells were collected and resuspended in a serum-free culture medium at a concentration of 10^5^ cells. The cell suspension was added to the upper chamber, with 200 μL of cell suspension (2 × 10^4^ cells/well) added to the lower chamber of the Transwell plate, which was filled with 800 μL of conditioned culture medium containing 20% FBS. After culturing in a 37 °C incubator for 24 h, the Transwell plate was removed, washed twice with PBS, fixed with water containing 10% formaldehyde for 10 min, and then rinsed three times with water. The cells were stained with 0.1% crystal violet and incubated at room temperature for 30 min, followed by two washes with PBS and removal of cells from the upper surface using a cotton ball. The invasion cells were photographed under an inverted light microscope (CKX53, Olympus, Japan), and ImageJ software was used for cell counting and analysis of invasive ability. For migration experiments, ECM gel coating on the Transwell plate was not required, and the remaining steps were the same as the invasion experiment [93, 94].

### TUNEL staining

The tested samples were fixed at room temperature using 4% paraformaldehyde for 15 min, followed by permeabilization with 0.25% Triton X-100 for 20 min. The samples were then blocked with 5% bovine serum albumin (BSA, 36101ES25, Shanghai Huahong Corporation) and stained with TUNEL reagent (C1086, Shanghai Beiyou Corporation). DAPI staining solution (C1002, Shanghai Beiyou Corporation) was used for additional staining of the slices in the dark. Apoptotic cell images were captured using a confocal microscope (LSM 880, Carl Zeiss AG, Germany). TUNEL-positive cells were indicated by green fluorescence, while DAPI was used for nuclear localization, with cells emitting blue fluorescence representing the total number of cells. Five different fields of view were selected for each group to calculate the apoptotic cell rate, which was determined by dividing the number of apoptotic cells by the total number of cells and multiplying by 100% [95].

### Flow cytometry analysis

The steps for detecting cell apoptosis using the V-FITC and PI dual staining kit (V13242, Invitrogen, USA) were as follows: The processed tumor cells were collected and washed three times with PBS. Then, these cells were treated with 5 μl of V-FITC and PI staining each for 15 min. Finally, flow cytometry analysis was performed using a flow cytometer (BD Bioscience, BD LSRFortessa, USA). The data were analyzed using CellQuest software (BD Bioscience, USA), where the lower-left quadrant represented normal cells, the upper-left quadrant represented necrotic cells, the upper-right quadrant represented late-stage apoptosis, and the lower-right quadrant represented early-stage apoptotic cells [96, 97].

### In vivo animal experiments

Male NOD/SCID mice, aged 6–8 weeks and weighing between 20 and 30 g, were purchased from Hunan Slakejingda Experimental Animal Co., Ltd. in Hunan, China. The mice were housed individually in cages in a specific pathogen-free (SPF) animal laboratory. The lab maintained a humidity level of 60–65% and a temperature of 22–25 °C. To allow the mice to acclimate to the environment, they were provided with ad libitum access to food and water and were subjected to a light-dark cycle of 12 h. Prior to the experiments, the mice were evaluated for their health status. All animal studies were conducted in accordance with the guidelines for the care and use of laboratory animals [98, 99].

U-2 OS/MG-63 cells were pre-treated, and a controlled quantity of 5 × 10^6^ cells was co-cultured. The cells were then injected subcutaneously into the left axillary of the mice. The tumor growth was observed promptly and documented by photography. Tumor volume was measured once daily. When the subcutaneous tumors reached ~3 mm in diameter, Dox was administered to the mice via intraperitoneal injection at a dose of 5 mg/kg twice a week. Two weeks after drug administration, the bioluminescence signal of the pre-treated U-2 OS/MG-63 OS cell line transplanted into the mice was analyzed using the CRi Maestro in vivo imaging system (Cambridge Research & Instrumentation, Massachusetts, USA). The mice were anesthetized with 2% isoflurane, and D-luciferin (150 mg/kg; 122799, PerkinElmer, USA) was injected intraperitoneally. Two photographs were taken at a 15-min interval, with an exposure time of 10 min [100]. Additionally, the transplant tumor volume was recorded every 7 days following drug injection, and a growth curve was plotted using the formula: (a*b^2^)/2, where “a” represents the longest diameter of the tumor and “b” represents the shortest diameter. After 28 days, the mice were euthanized with CO_2_, and the transplanted tumors were excised and weighed. The tumor tissues were used for Western blot and immunohistochemical analysis [98].

The mice were randomly divided into 12 groups, with 6 mice in each group: (1) TIMM23-WT + oe-NC + PBS/Dox group - U-2 OS/MG-63 OS cells with TIMM23 intact were co-cultured with and transfected with oe-NC lentivirus, and the bottom layer of cells was collected after 48 h for injection. (2) TIMM23-KO + oe-NC + PBS/Dox group - U-2 OS/MG-63 OS cells with TIMM23 knocked out were co-cultured with and transfected with oe-NC lentivirus, and the bottom layer of cells was collected after 48 h for injection. (3) TIMM23-KO + TIMM23-PARGP1-FLAG + PBS/Dox group - U-2 OS/MG-63 OS cells with TIMM23 knocked out were co-cultured with and transfected with oe-TIMM23-PARGP1-FLAG lentivirus, and the bottom layer of cells was collected after 48 h for injection. In all groups, the mice were treated with PBS/Dox, and the OS cell line used was U-2 OS/MG-63 [98].

### Histological staining

Hematoxylin and Eosin (H&E) staining: The tissue samples were obtained and fixed, followed by sectioning and removal of the paraffin blocks in xylene. Subsequently, the sections were rehydrated in 100% ethanol, 95% ethanol, and 70% ethanol or washed with water. The prepared sections were then stained with H&E (H8070, Solarbio, Beijing, China) for 5–10 min at room temperature. After rinsing with distilled water and dehydrating in 95% ethanol, the sections were placed in an Eosin staining solution (G1100, Solarbio, Beijing) for 5–10 min. Finally, routine dehydration, clearing, and mounting steps were performed [101].

### Immunohistochemical staining

The subcutaneous tumor tissues from mice were fixed overnight in 4% paraformaldehyde and then embedded in paraffin with a thickness of 4 μm. After deparaffinization in xylene, the sections were rehydrated with a gradient of ethanol, including absolute ethanol, 95% ethanol, and 75% ethanol, each for 3 min. Subsequently, the sections were placed in a 0.01 M citrate buffer solution and boiled for 15–20 min for antigen retrieval. Then, the sections were incubated with 3% H_2_O_2_ at room temperature for 30 min to inactivate endogenous peroxidases. Following that, the sections were blocked with goat serum for 20 min at room temperature and excess liquid was removed. The sections were then incubated with Ki67 antibody (ab16667, 1:200, Abcam) as the primary antibody, followed by incubation at room temperature for 1 h and subsequent washing with PBS. Subsequently, the sections were incubated with IgG (ab150077, 1:1000, Abcam) as the secondary antibody, followed by incubation at 37 °C for 20 min and washing with PBS. Then, the sections were incubated with SP (Streptavidin Peroxidase) at 37 °C for 30 min, washed with PBS, and stained with DAB (P0202, Beyotime Biotechnology Co., Ltd) for 5–10 min for color development. The reaction was stopped by rinsing with water for 10 min. After counterstaining with Hematoxylin (C0107, Beyotime Biotechnology Co., Ltd) for 2 min, differentiation with hydrochloric acid alcohol, and washing with water for 10 min, the sections were dehydrated in a gradient of ethanol (with xylene as the clearing agent). Finally, 2–3 drops of neutral resin were added for mounting. The sections were observed and analyzed under a light microscope: five high-power fields were randomly selected from each section, with 100 cells observed in each field, and the positive rate of Ki67 cells was calculated [102].

### Statistical analysis

Our study employed version 4.2.1 of the R language, with compilation performed through the integrated development environment RStudio, which was version 2022.12.0-353. For file processing, we utilized version 5.30.0 of the Perl language. Additionally, we utilized GraphPad Prism software, specifically version 8.0.

To represent quantitative data, we employed the mean plus/minus standard deviation. For comparing two sets of data, we utilized an independent samples t-test [103]. For comparing data among different groups, we employed one-way analysis of variance (ANOVA), while for comparing data among different time points within groups, we utilized two-way ANOVA. Post hoc testing was conducted using Bonferroni correction. The significance threshold was set at *P* < 0.05 [104].

## Supplementary information

Full and uncropped western blots

Supplementary materials

## Data Availability

Data will be made available on request.
