# Supplementary material for: Targeting TIMM23 to overcome osteosarcoma chemoresistance
Source: Cell Death Dis. 2025 Nov 24;16(1):856. doi: 10.1038/s41419-025-08106-w (PMC12644526; doi:10.1038/s41419-025-08106-w)
Supplement: Supplementary file 1 — Full and uncropped western blots [file 41419_2025_8106_MOESM1_ESM.docx]

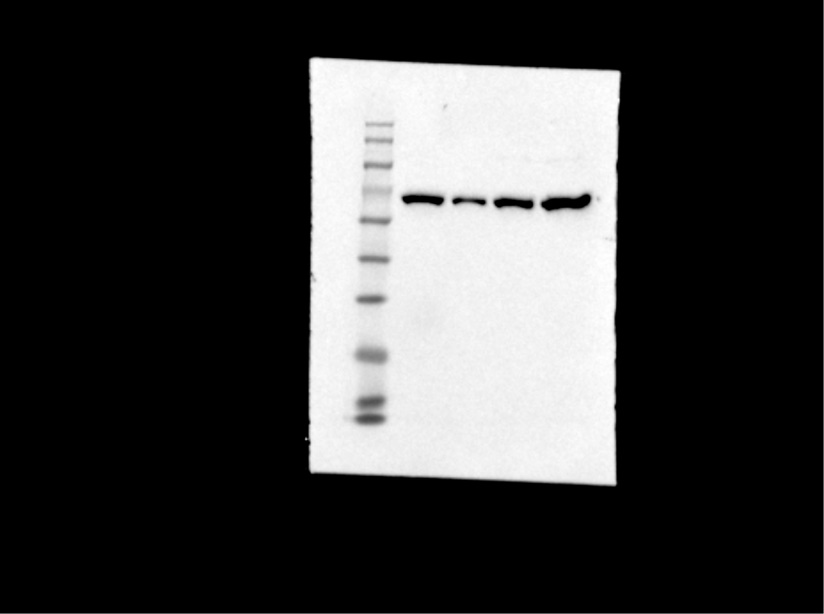


Figure 3F-1


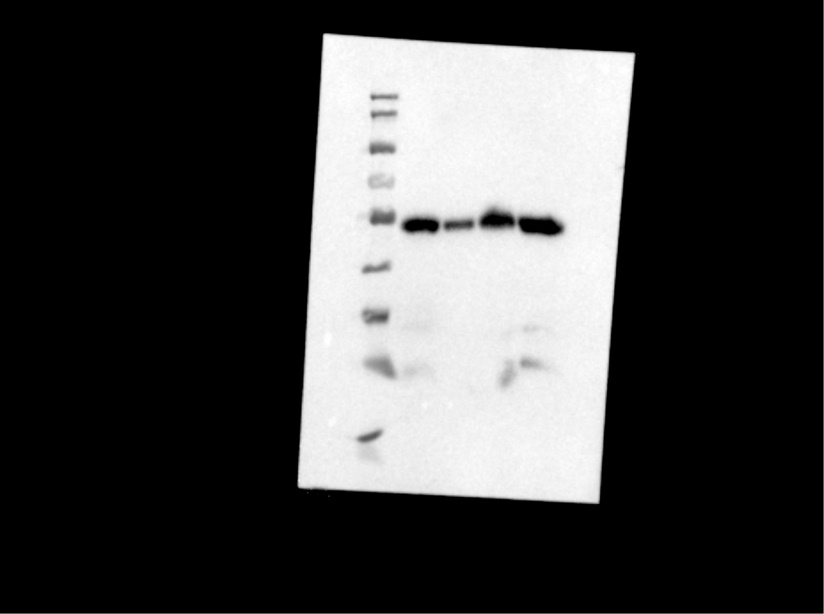


Figure 3F-2


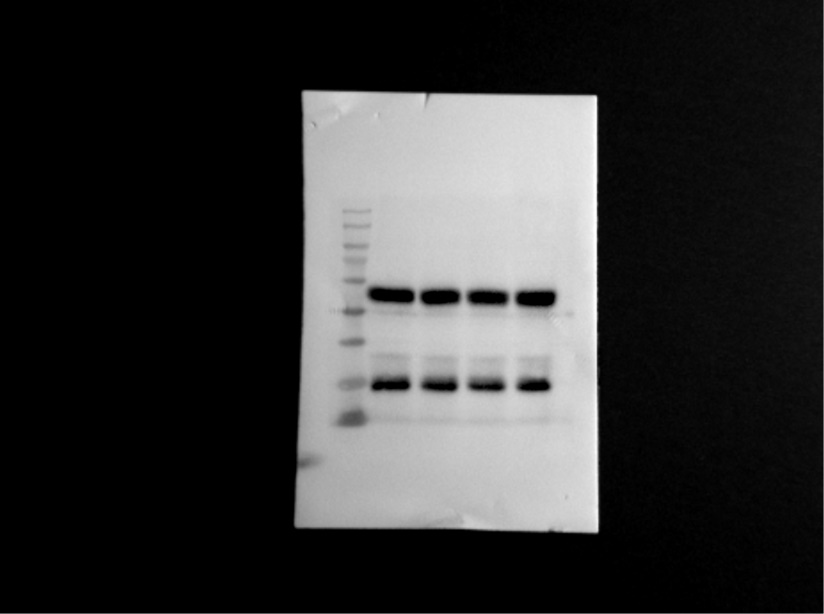


Figure 3F-3


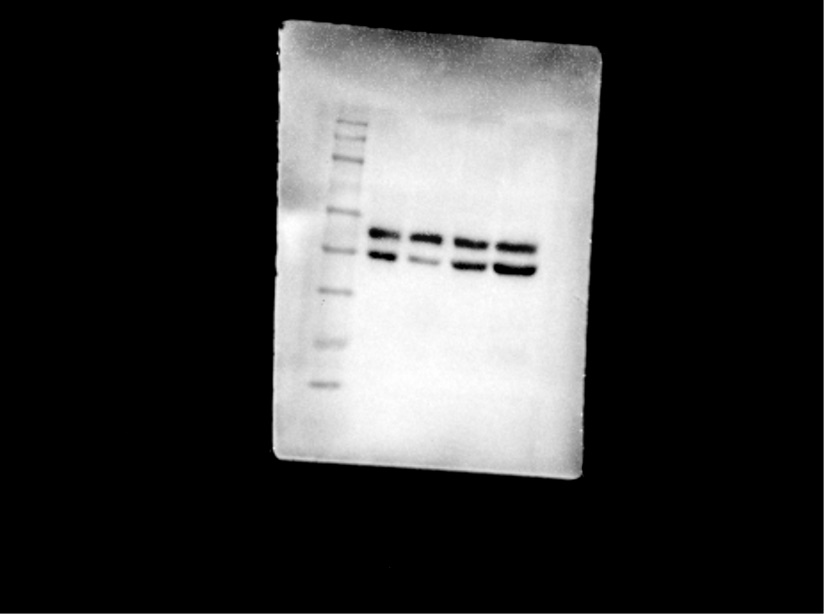


Figure 3F-4


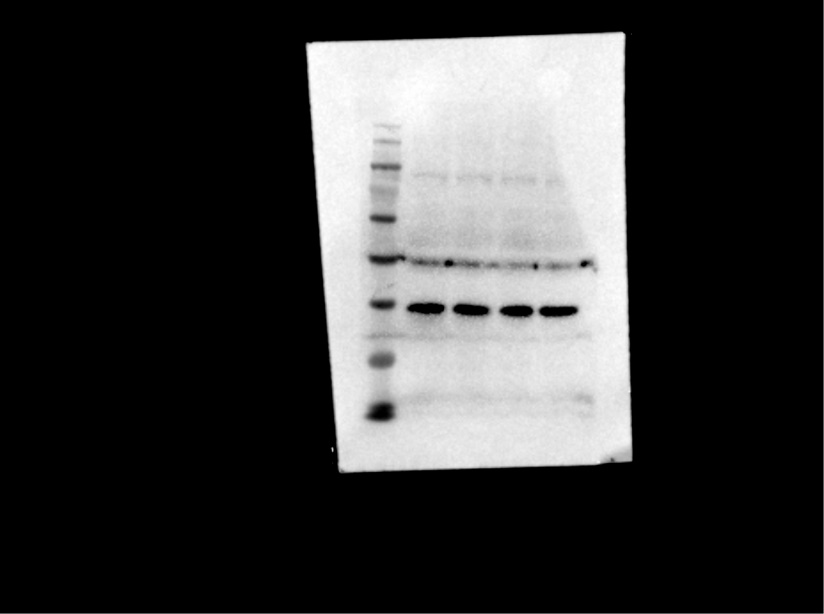


Figure 3F-5


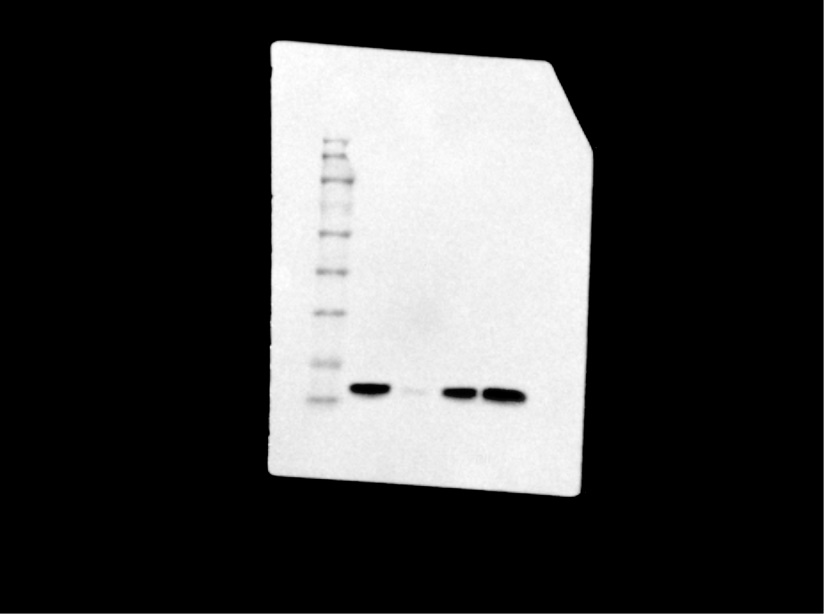


Figure 5D-1


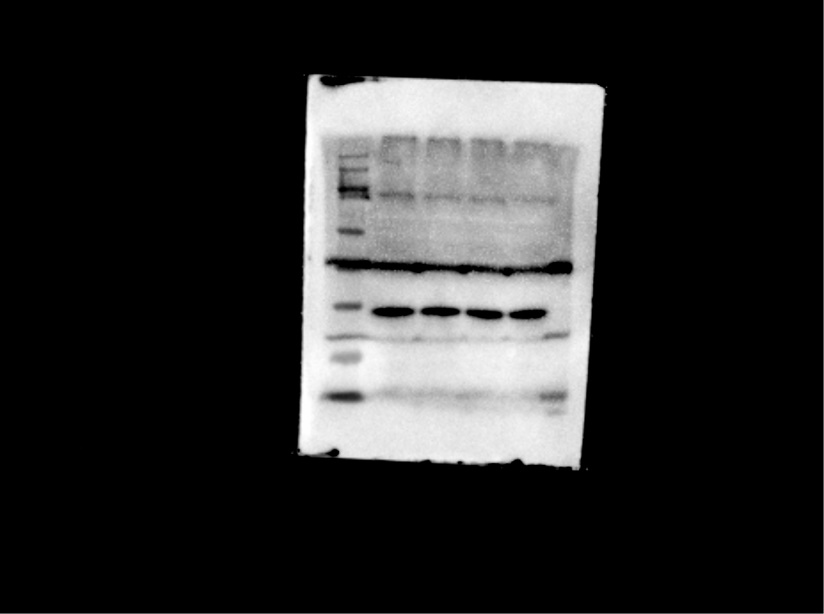


Figure 5D-2


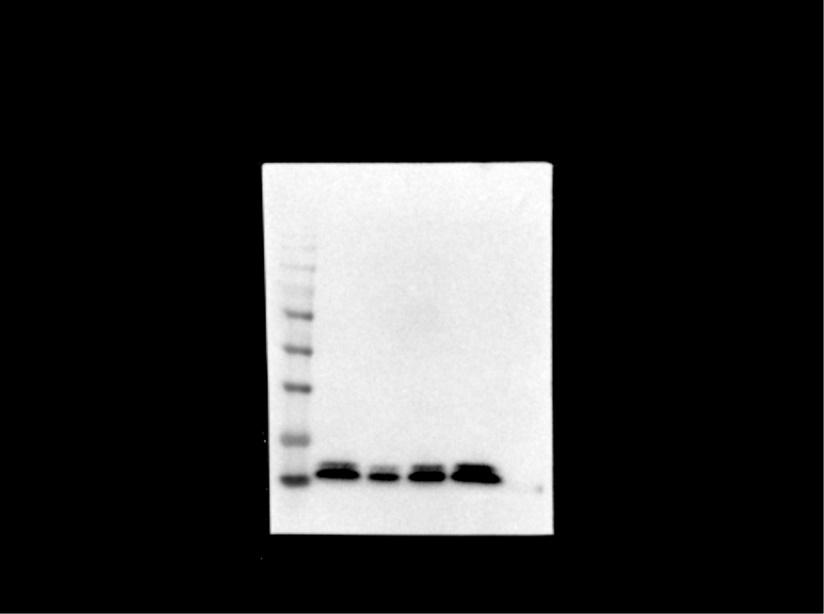


Figure 5E-1


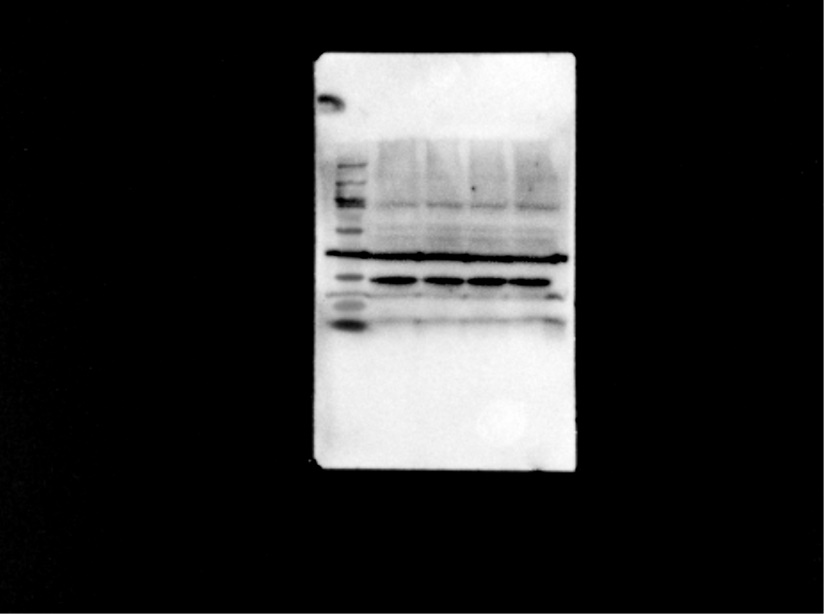


Figure 5E-2


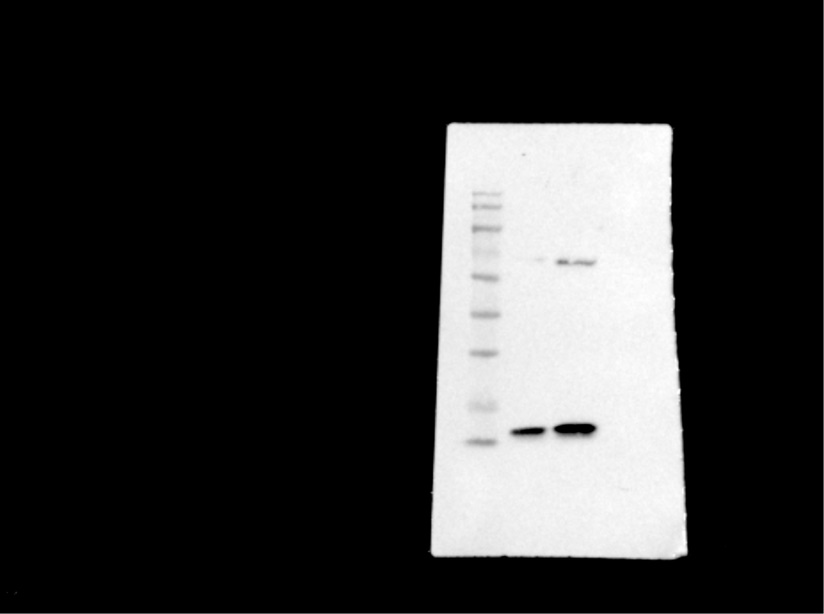


Figure 5G-1


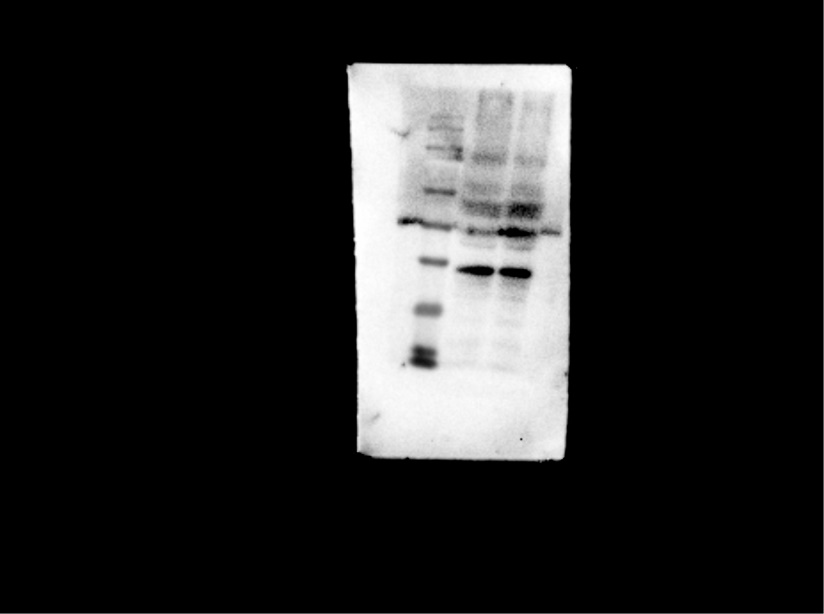


Figure 5G-2


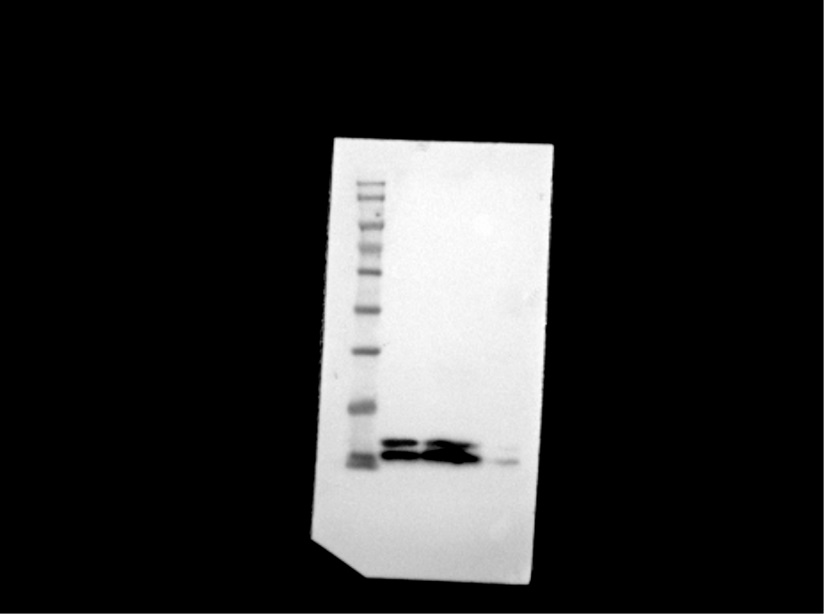


Figure 5G-3


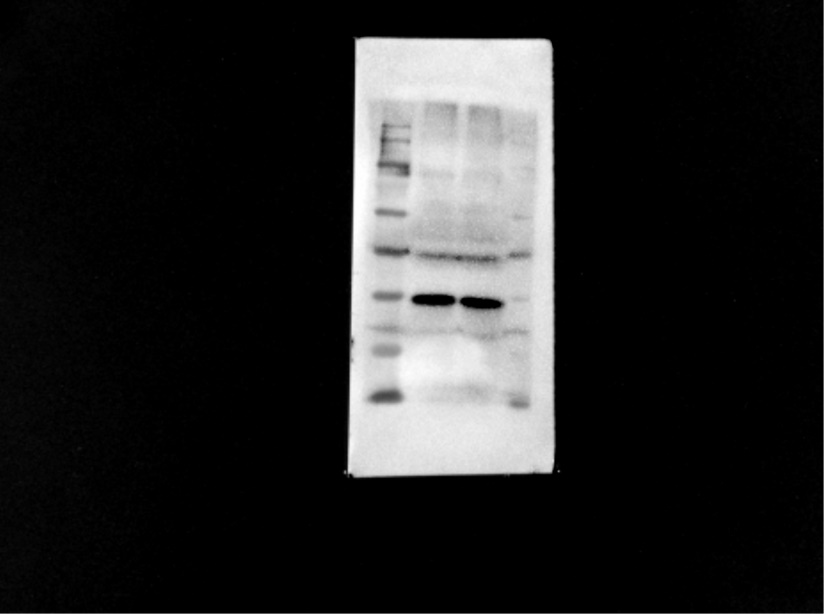


Figure 5G-4


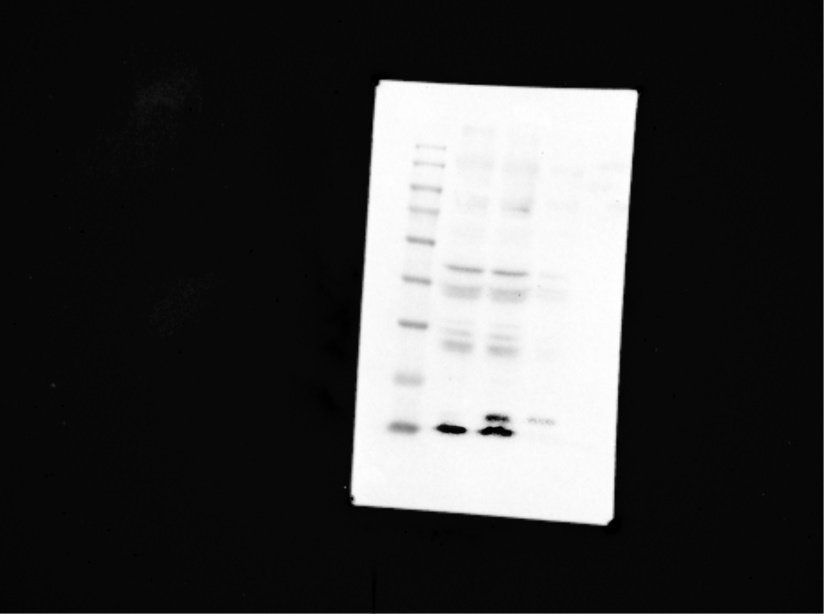


Figure 5H-1


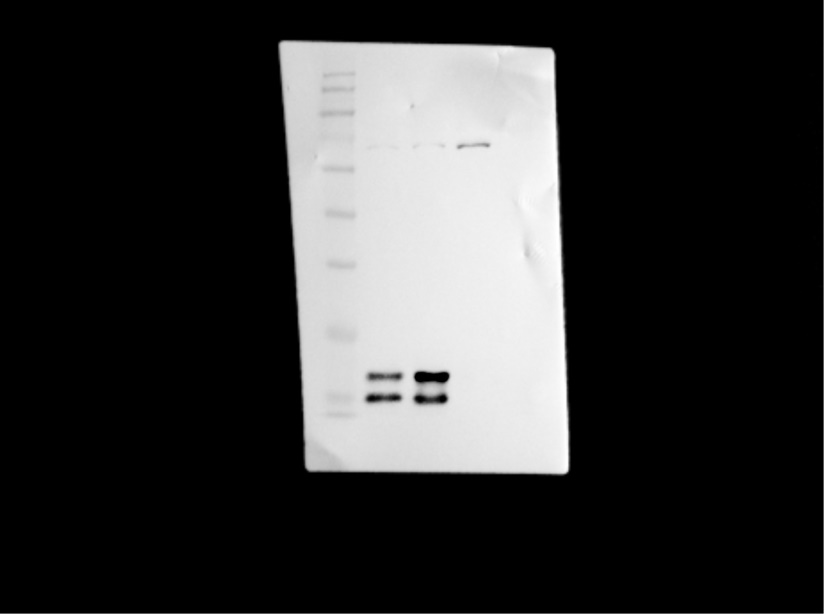


Figure 5H-2


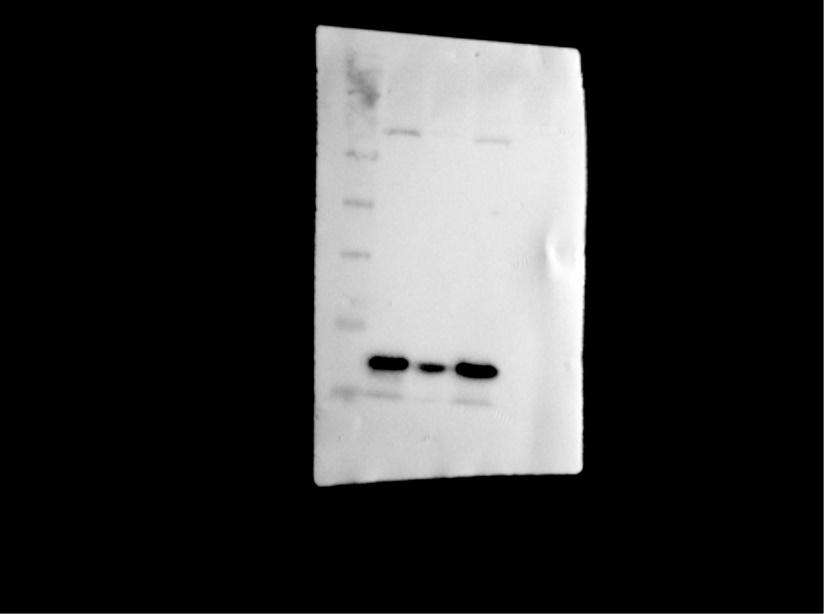


Figure 5I-1


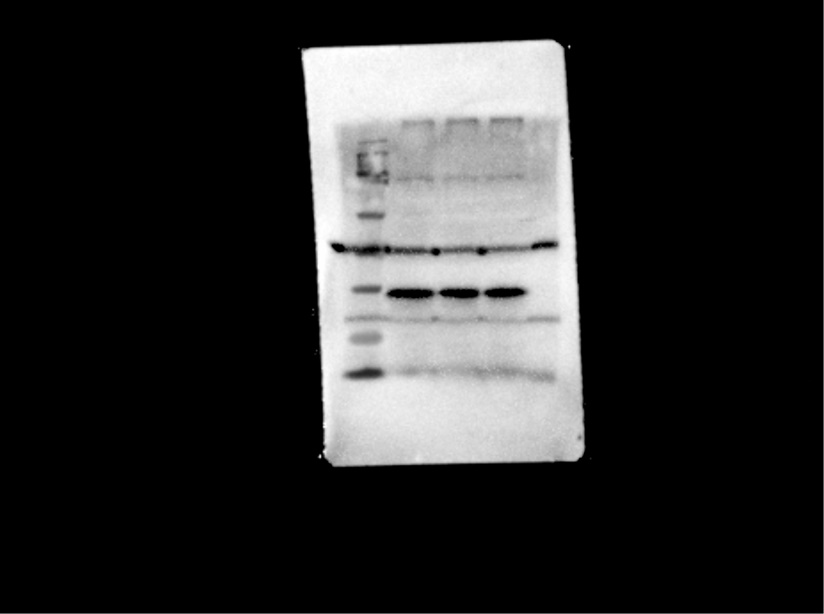


Figure 5I-2


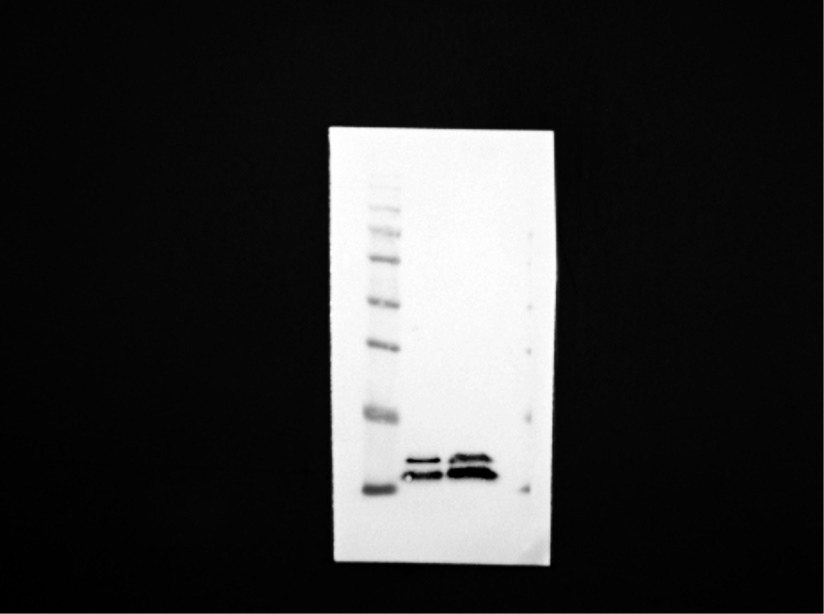


Figure 6A-1-1


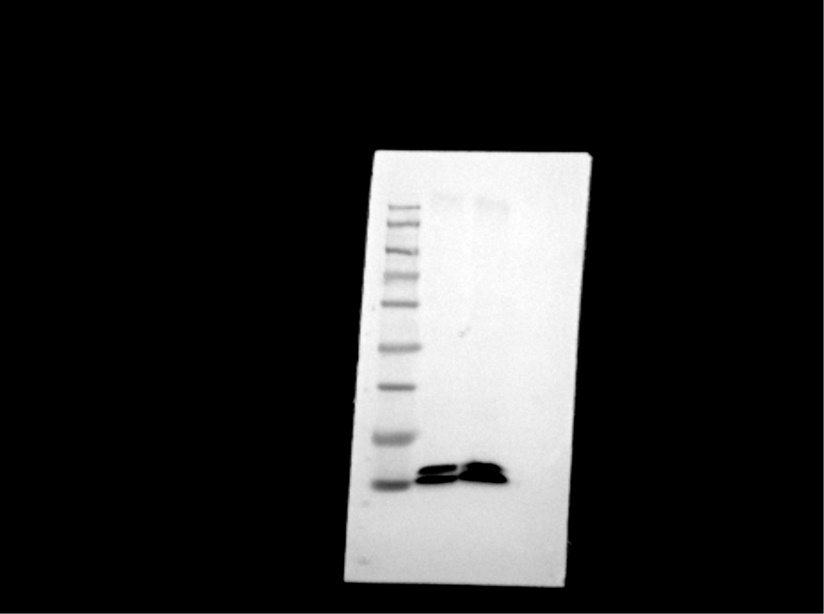


Figure 6A-1-2


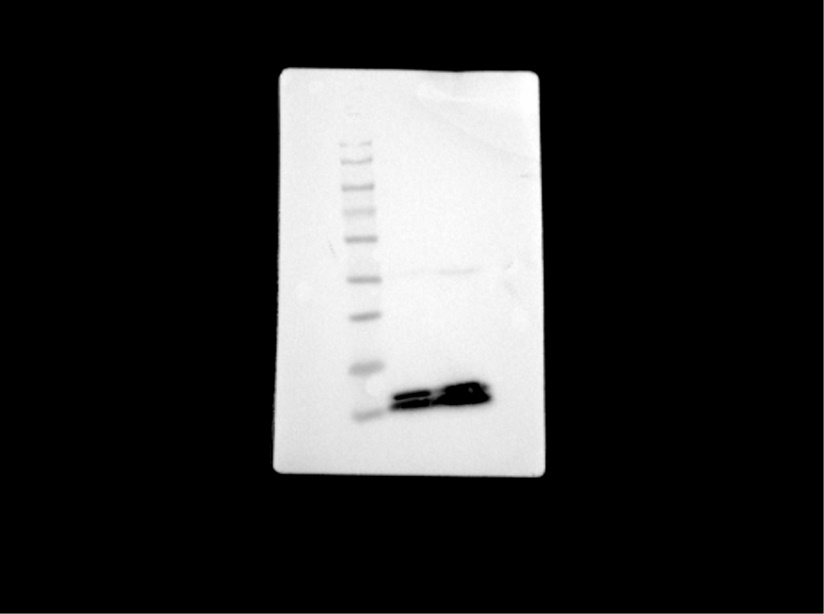


Figure 6A-1-3


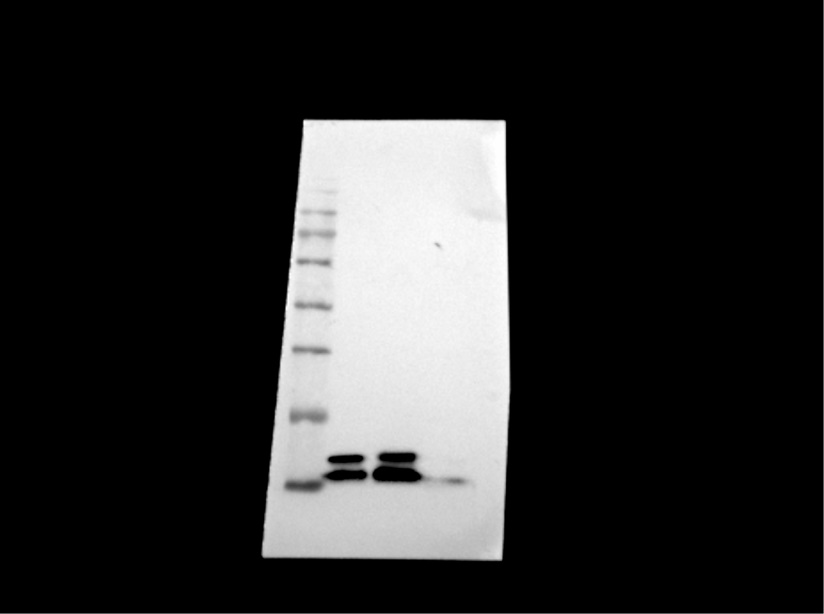


Figure 6A-1-4


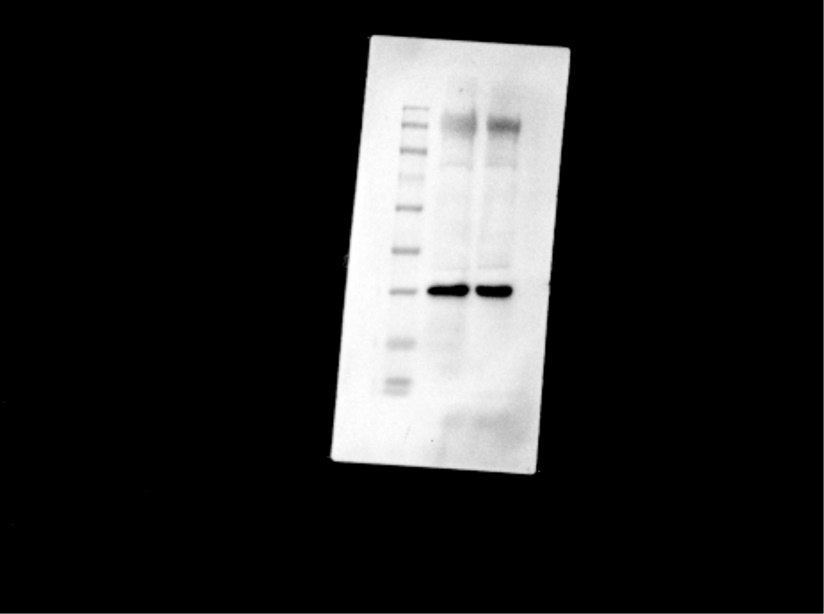


Figure 6A-2 (1)


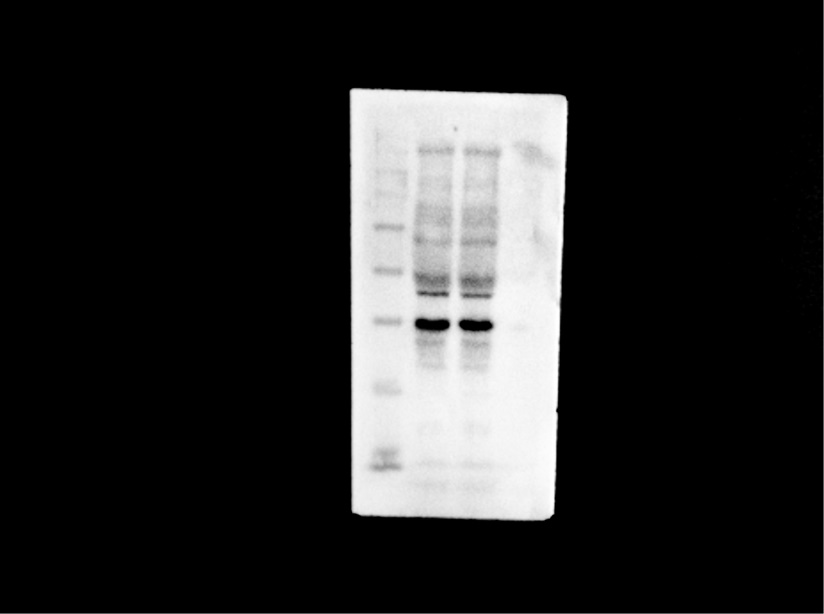


Figure 6A-2 (2)


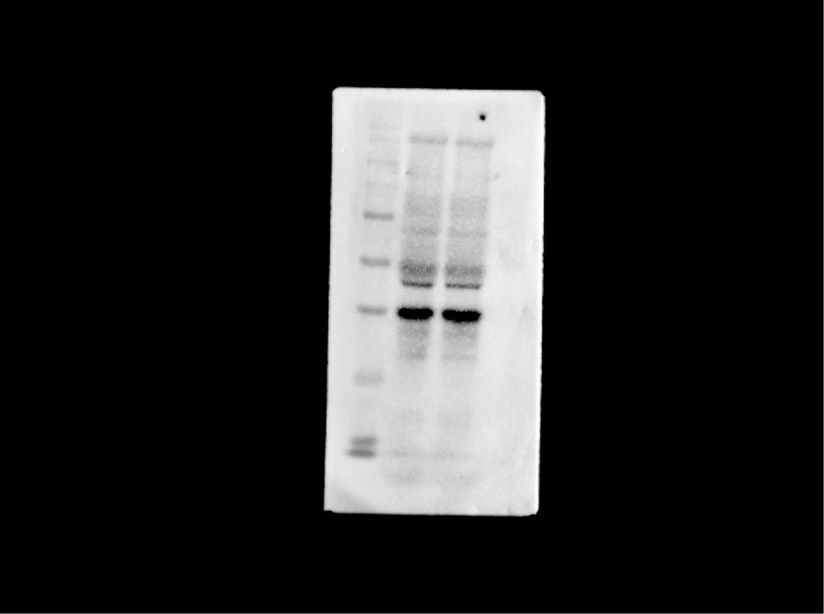


Figure 6A-2 (3)


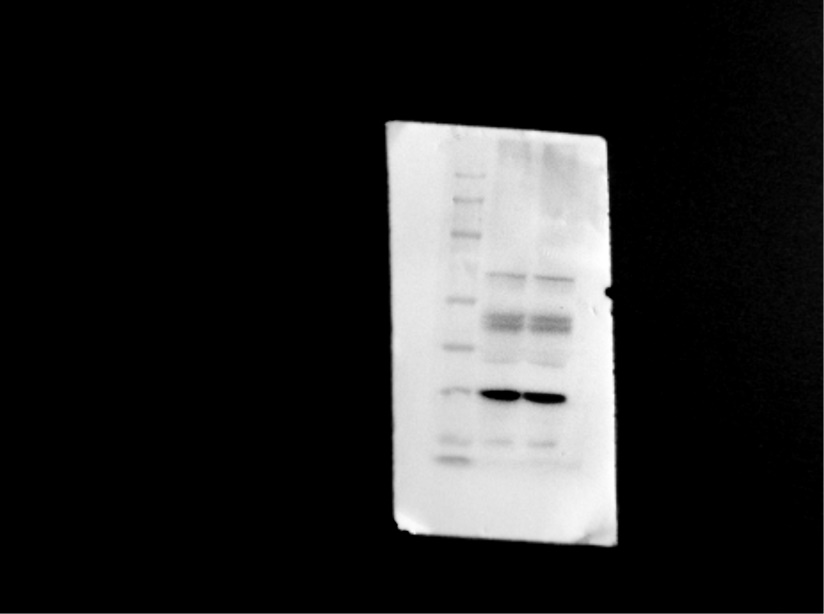


Figure 6A-2 (4)


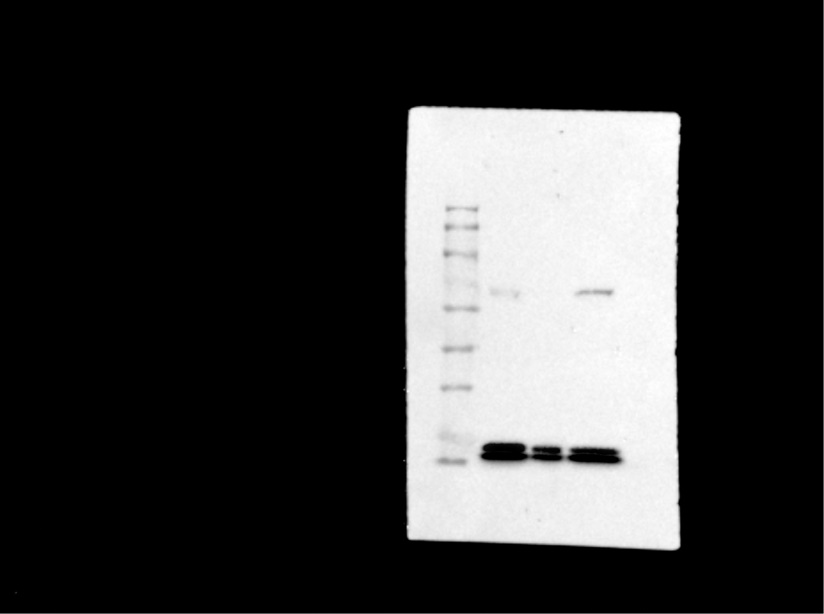


Figure 8A-1


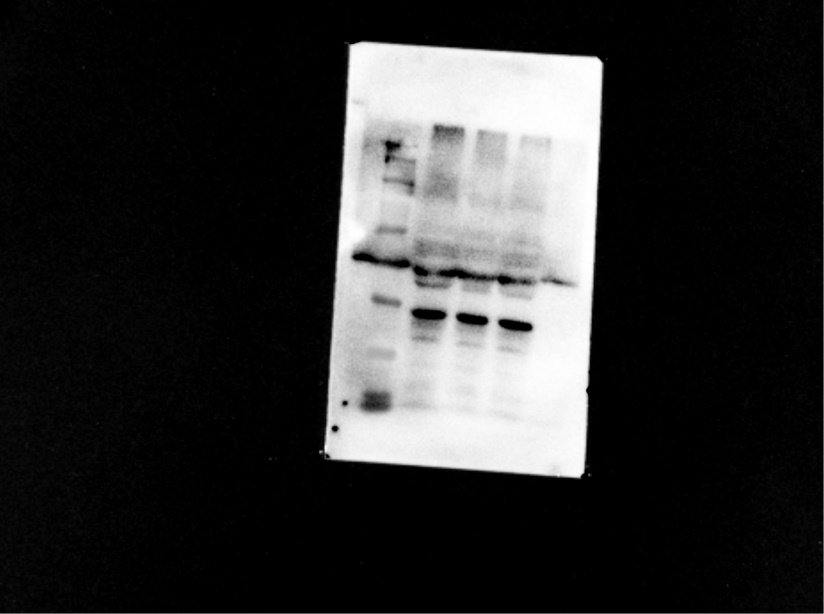


Figure 8A-2


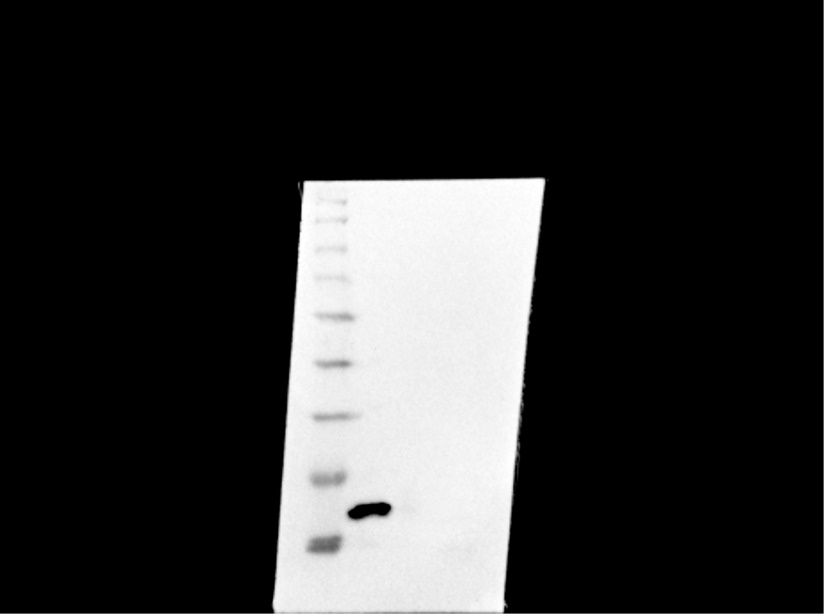


Figure S5B-1


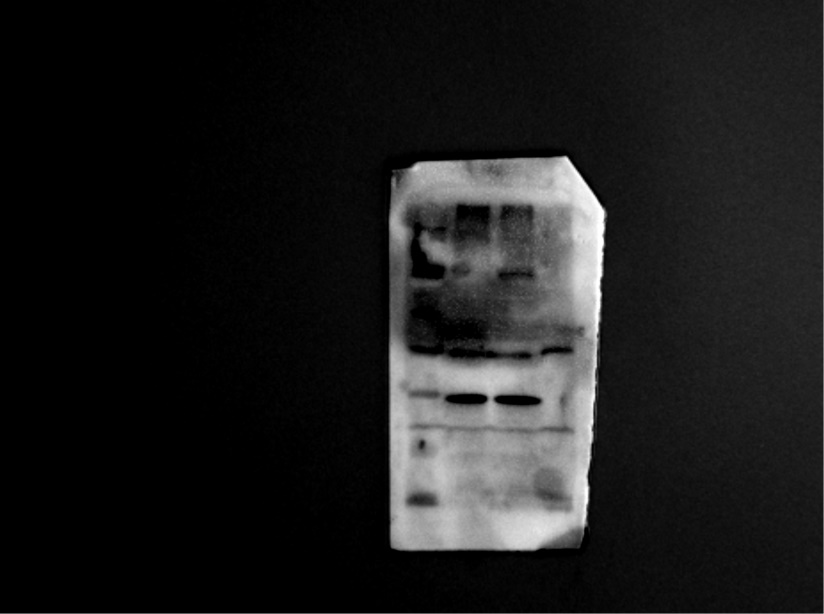


Figure S5B-2


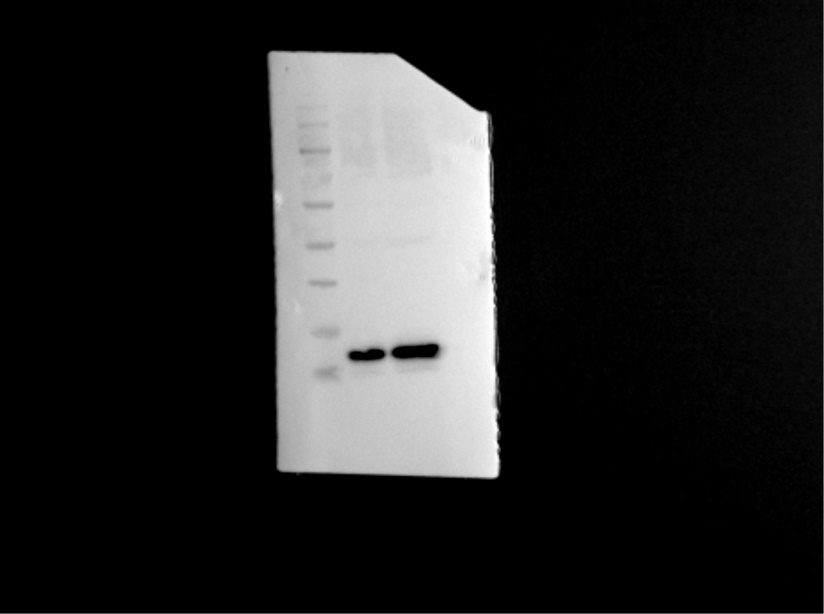


Figure S5D-1


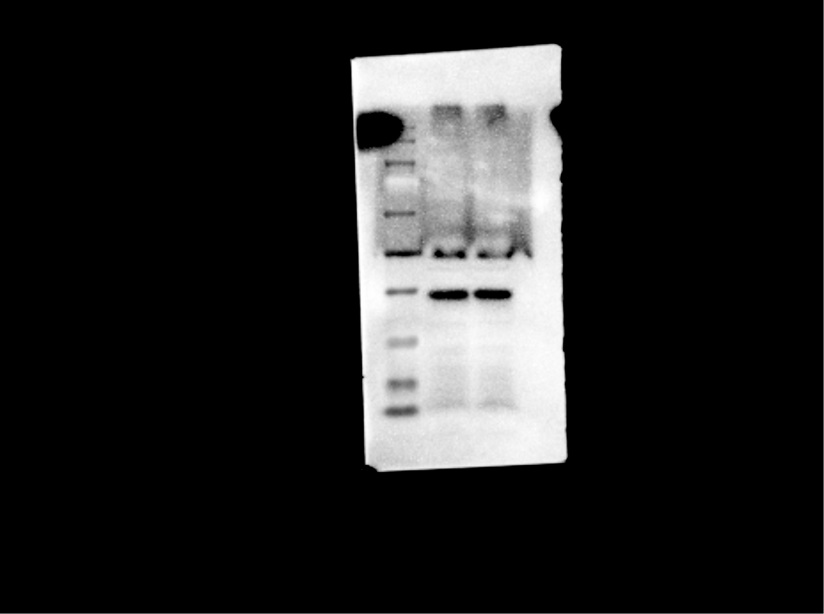


Figure S5D-2


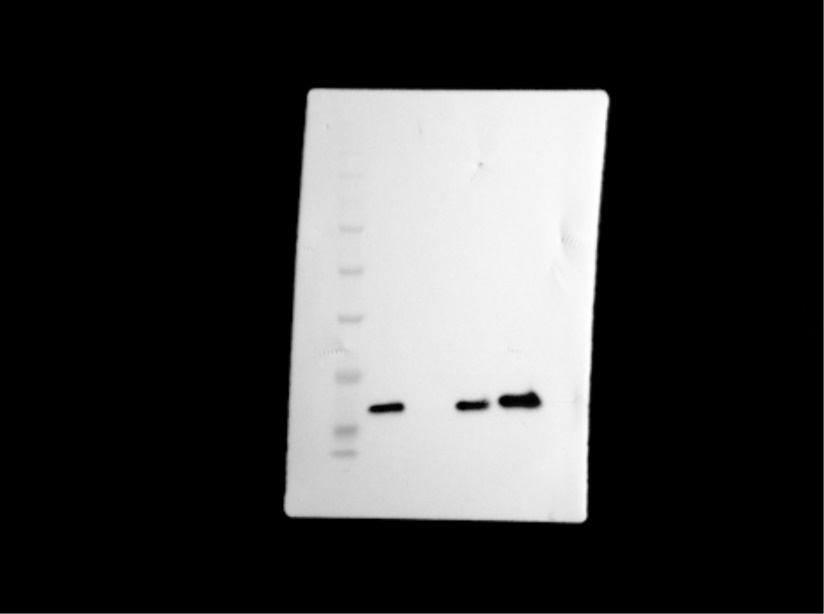


Figure S9A-1


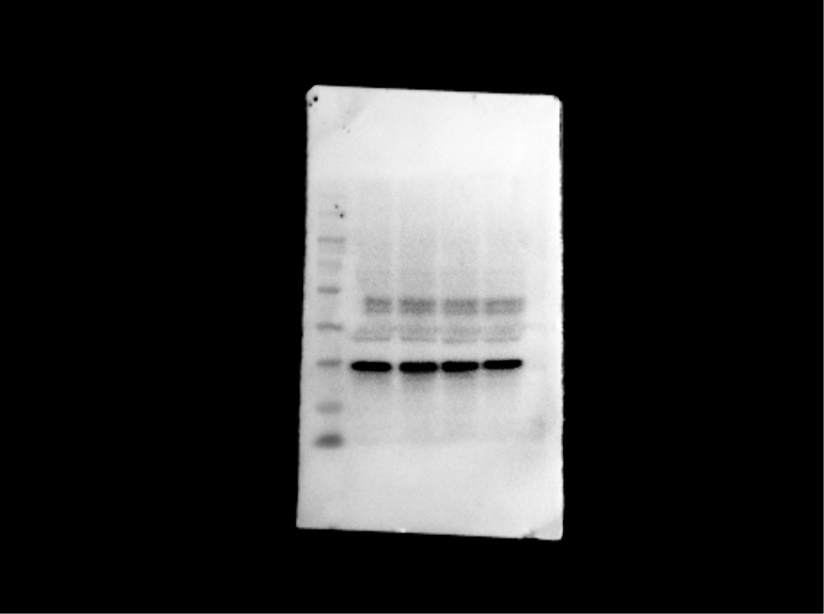


Figure S9A-2


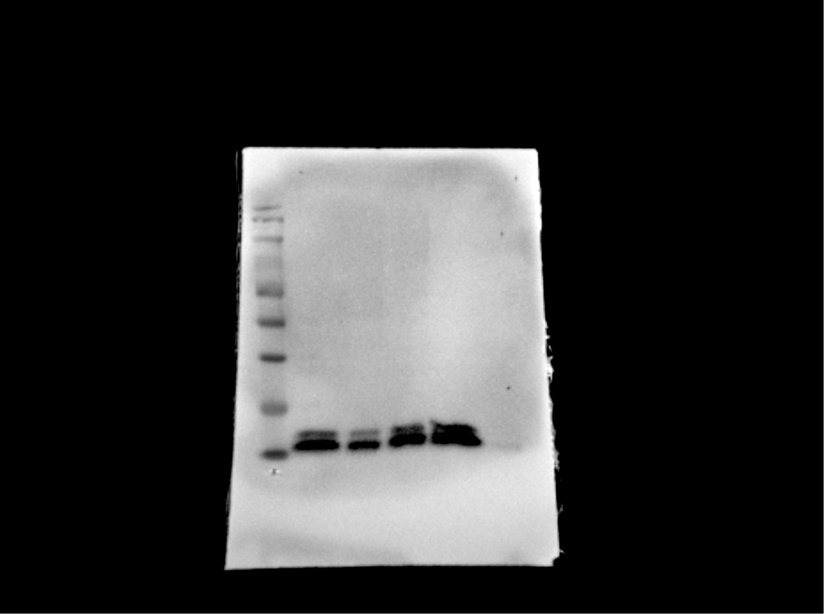


Figure S9B-1


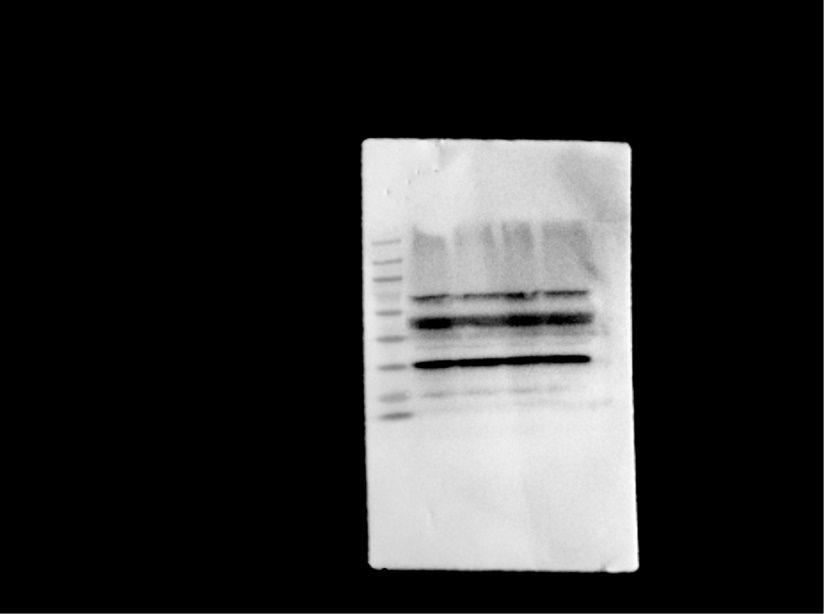


Figure S9B-2


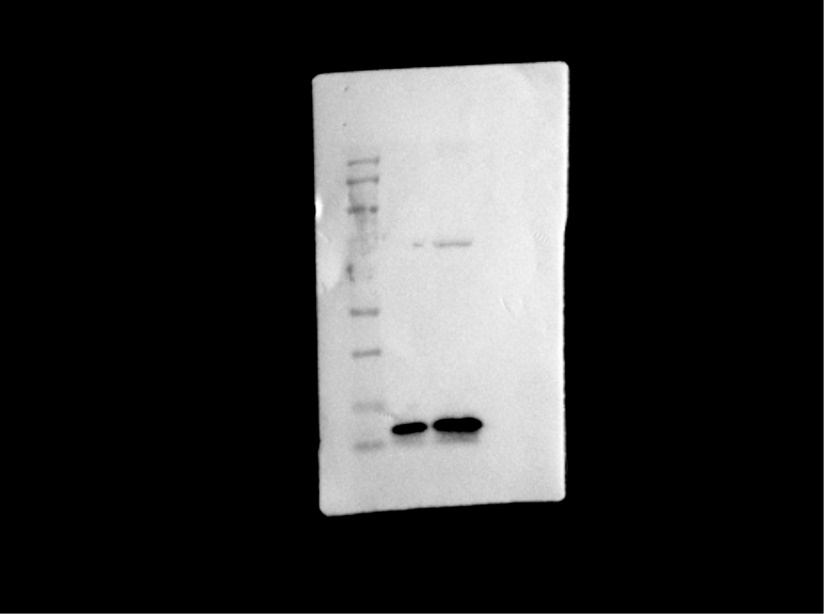


Figure S9C-1-1


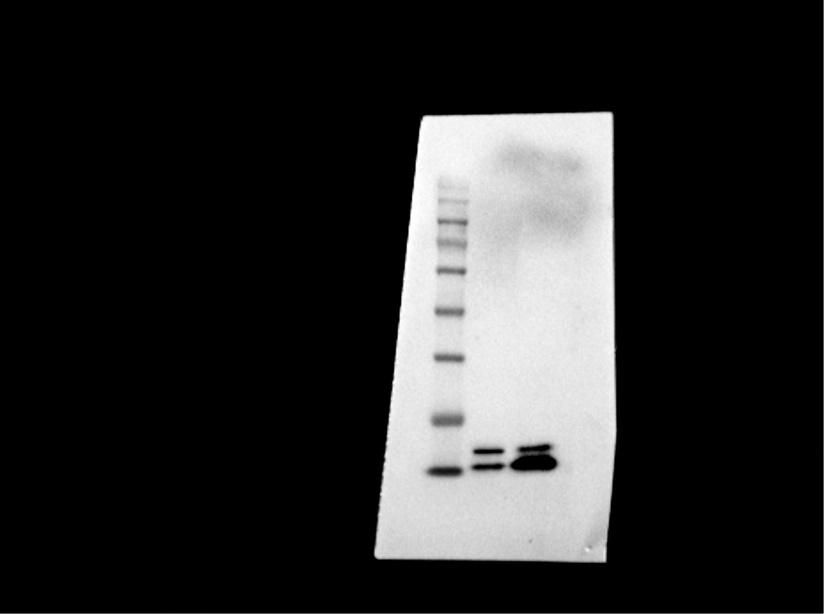


Figure S9C-1-2


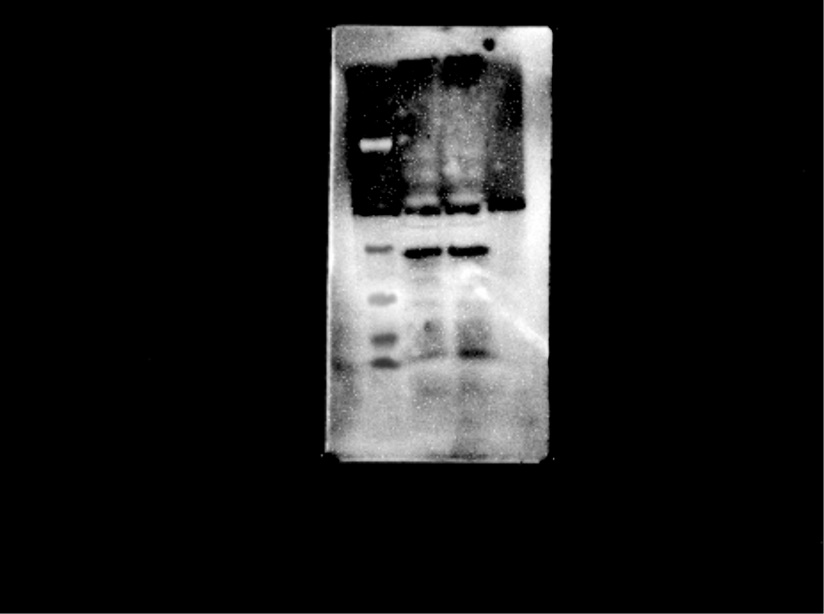


Figure S9C-2-1


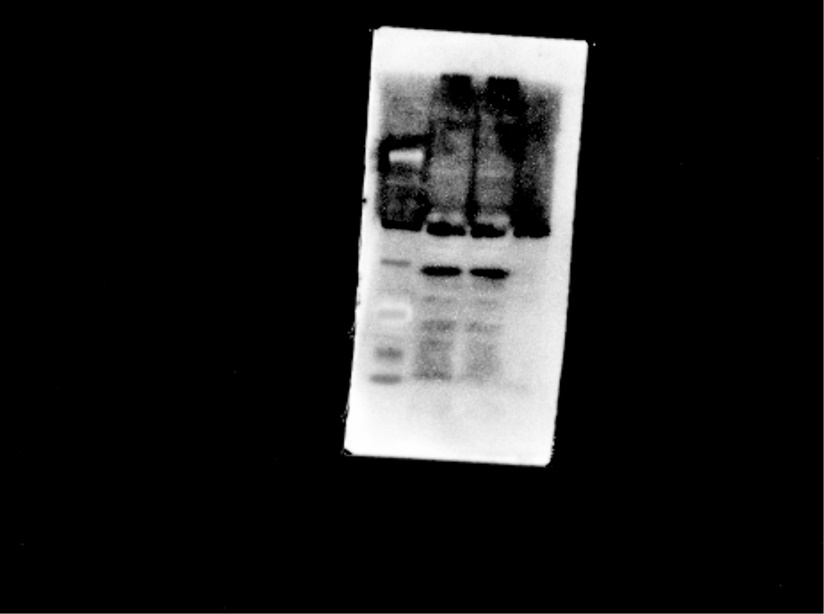


Figure S9C-2-2


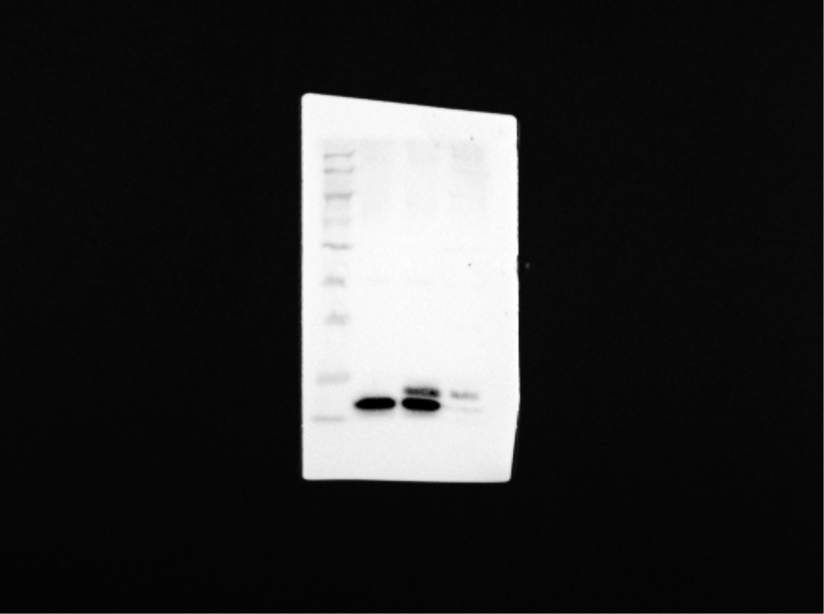


Figure S9D-1


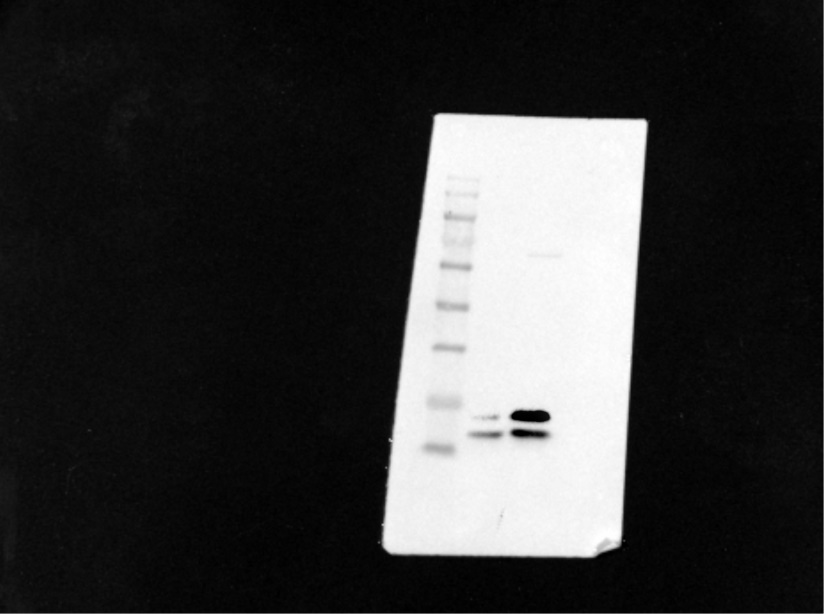


Figure S9D-2


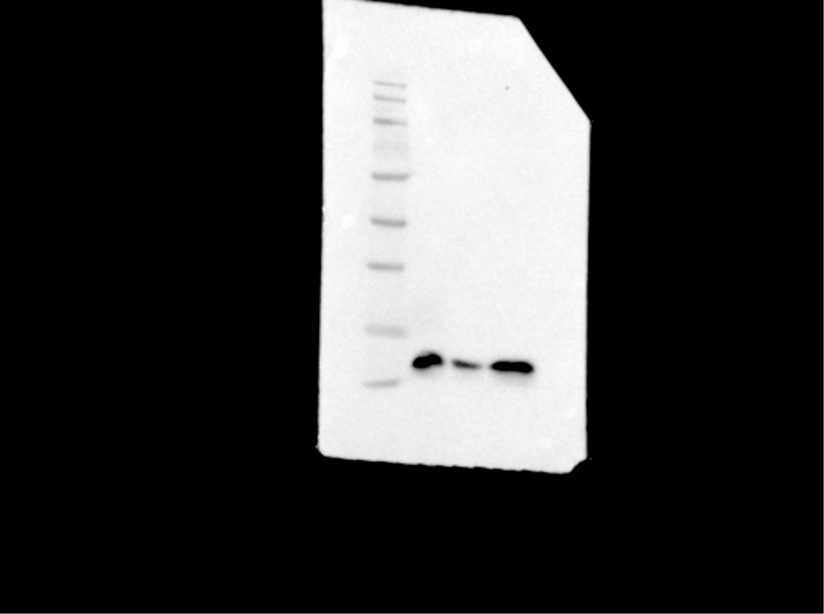


Figure S9E-1


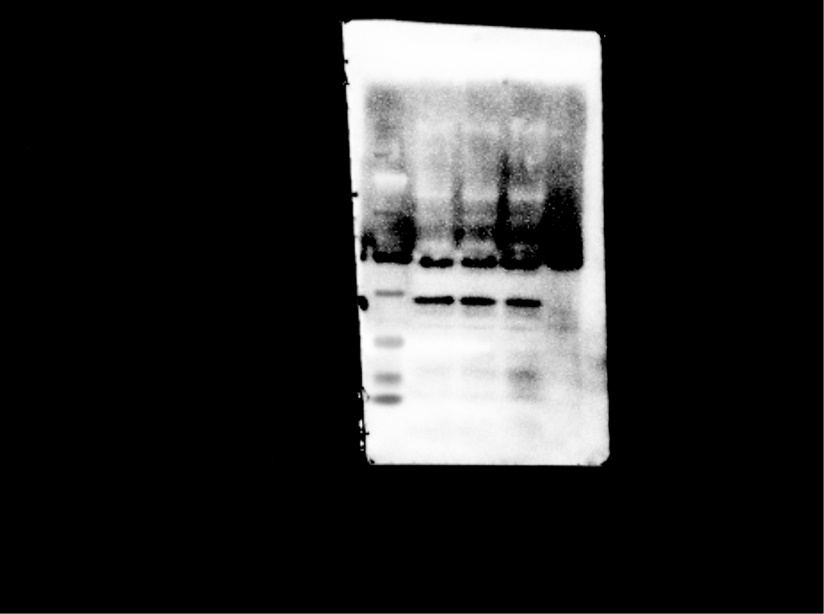


Figure S9E-2


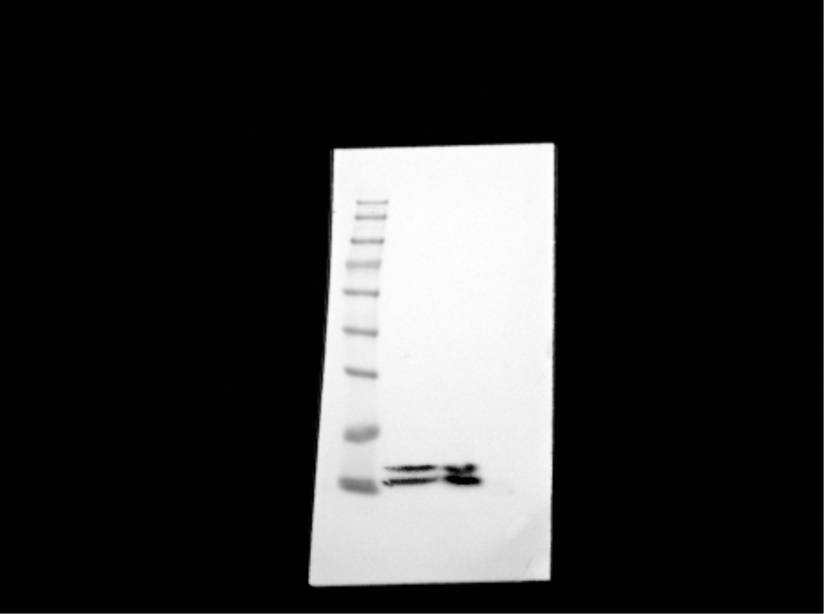


Figure S10A-1-1


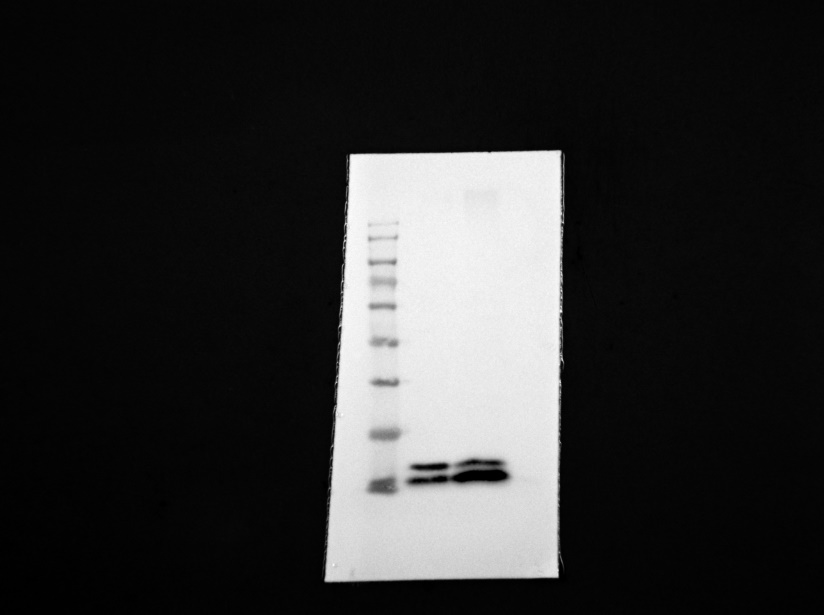


Figure S10A-1-2


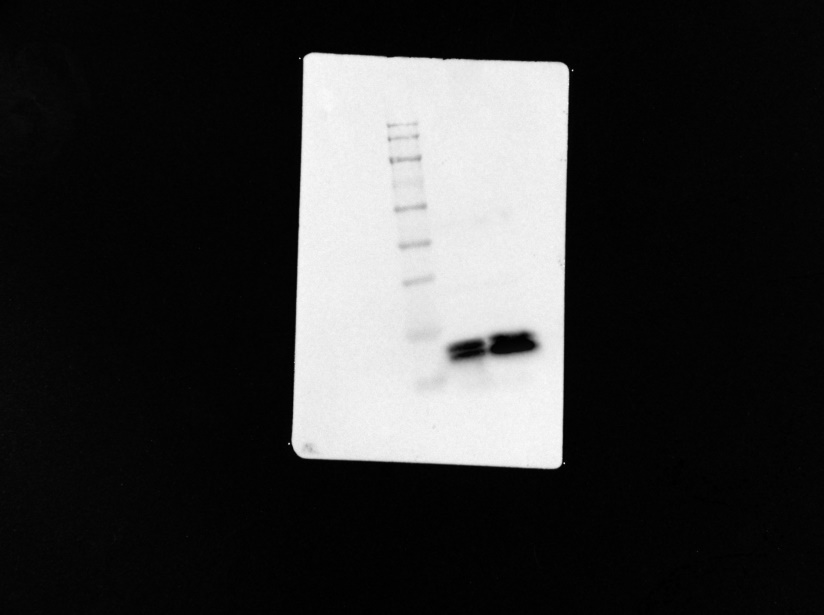


Figure S10A-1-3


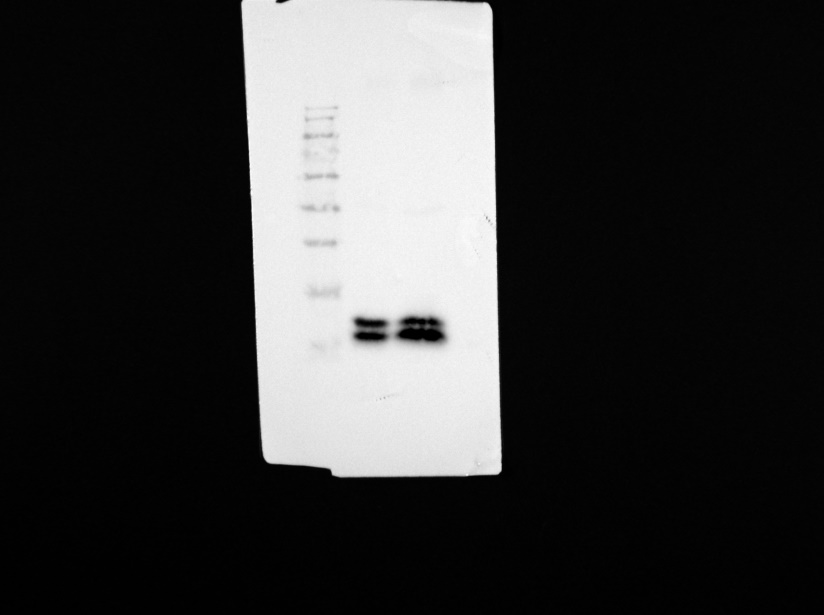


Figure S10A-1-4


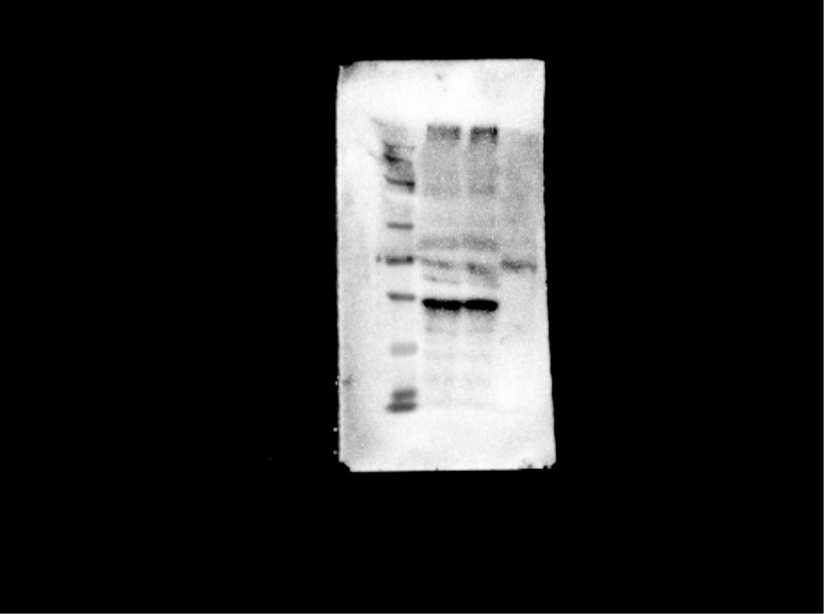


Figure S10A-2 (1)


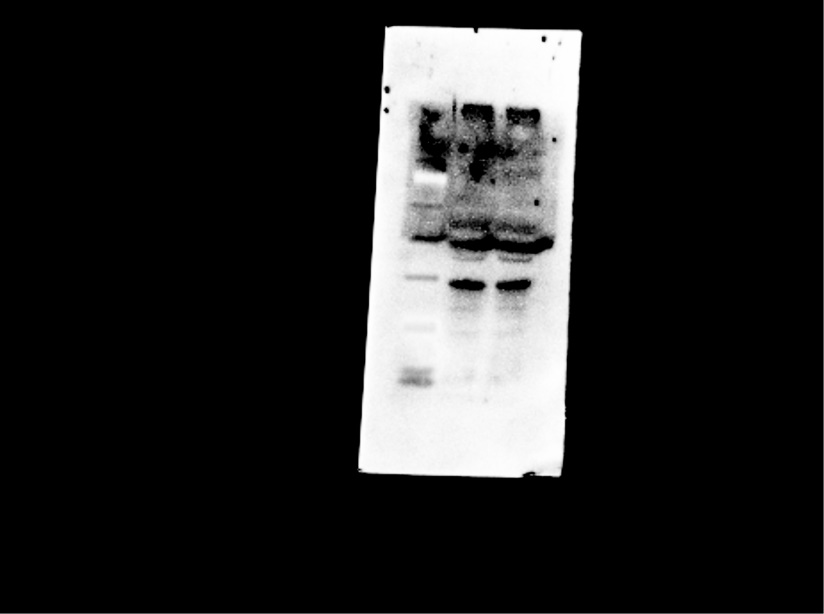


Figure S10A-2 (2)


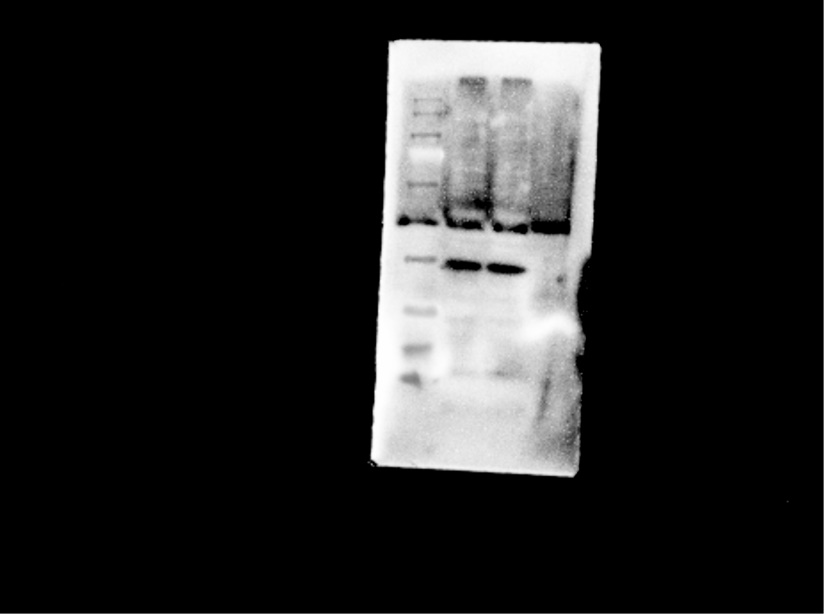


Figure S10A-2 (3)


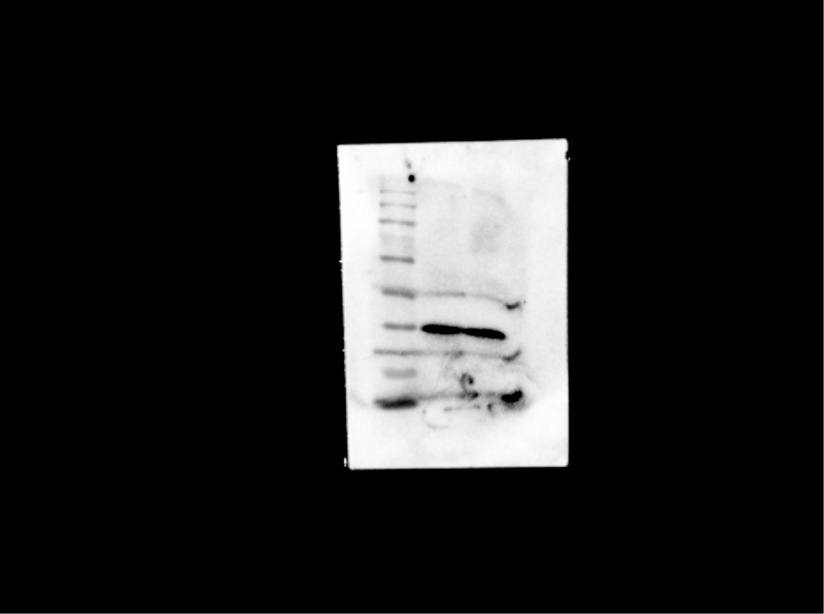


Figure S10A-2 (4)


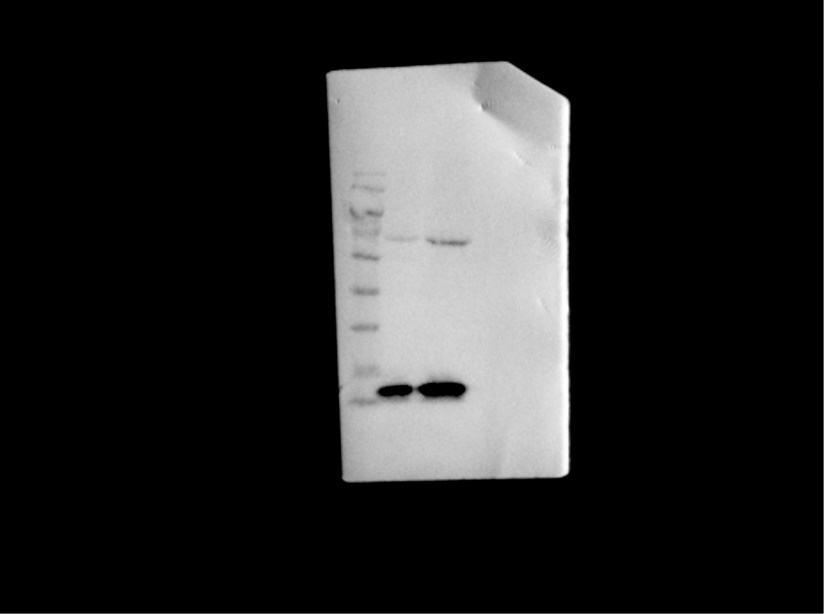


Figure S12A-1


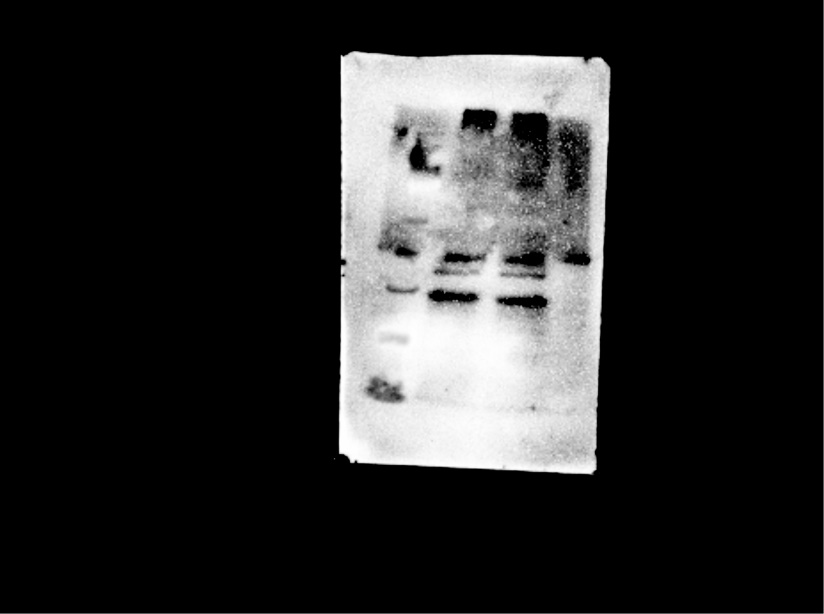


Figure S12A-2


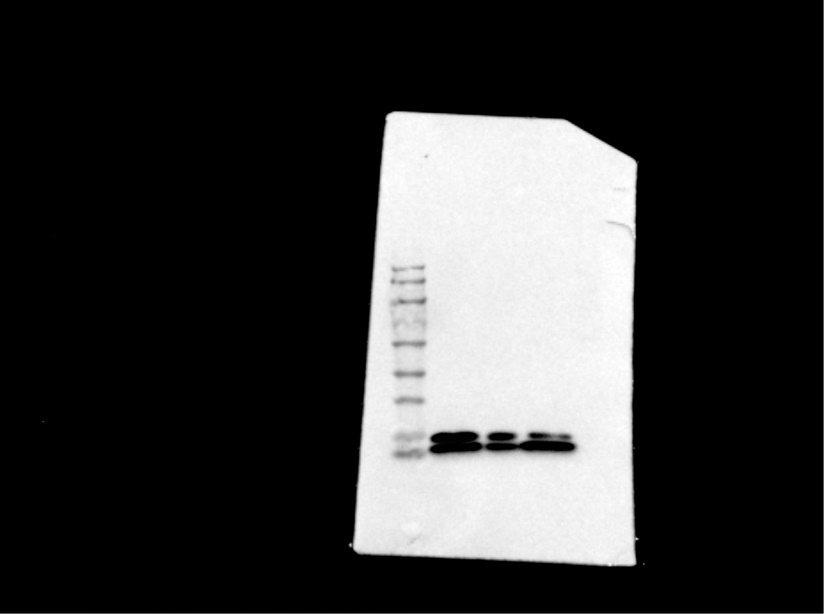


Figure S12B-1


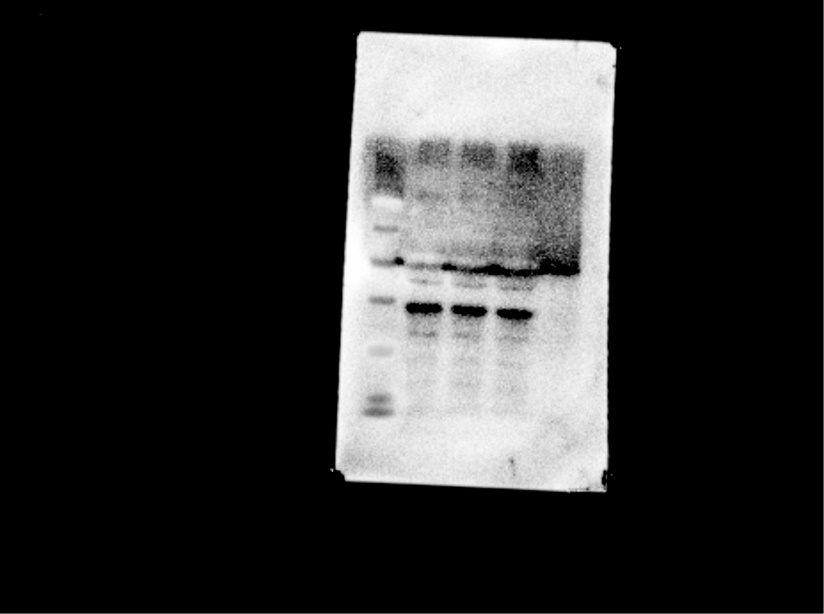


Figure S12B-2
