# Supplementary material for: Targeting TIMM23 to overcome osteosarcoma chemoresistance
Source: Cell Death Dis. 2025 Nov 24;16(1):856. doi: 10.1038/s41419-025-08106-w (PMC12644526; doi:10.1038/s41419-025-08106-w)
Supplement: Supplementary file 2 — Supplementary materials [file 41419_2025_8106_MOESM2_ESM.docx]

**
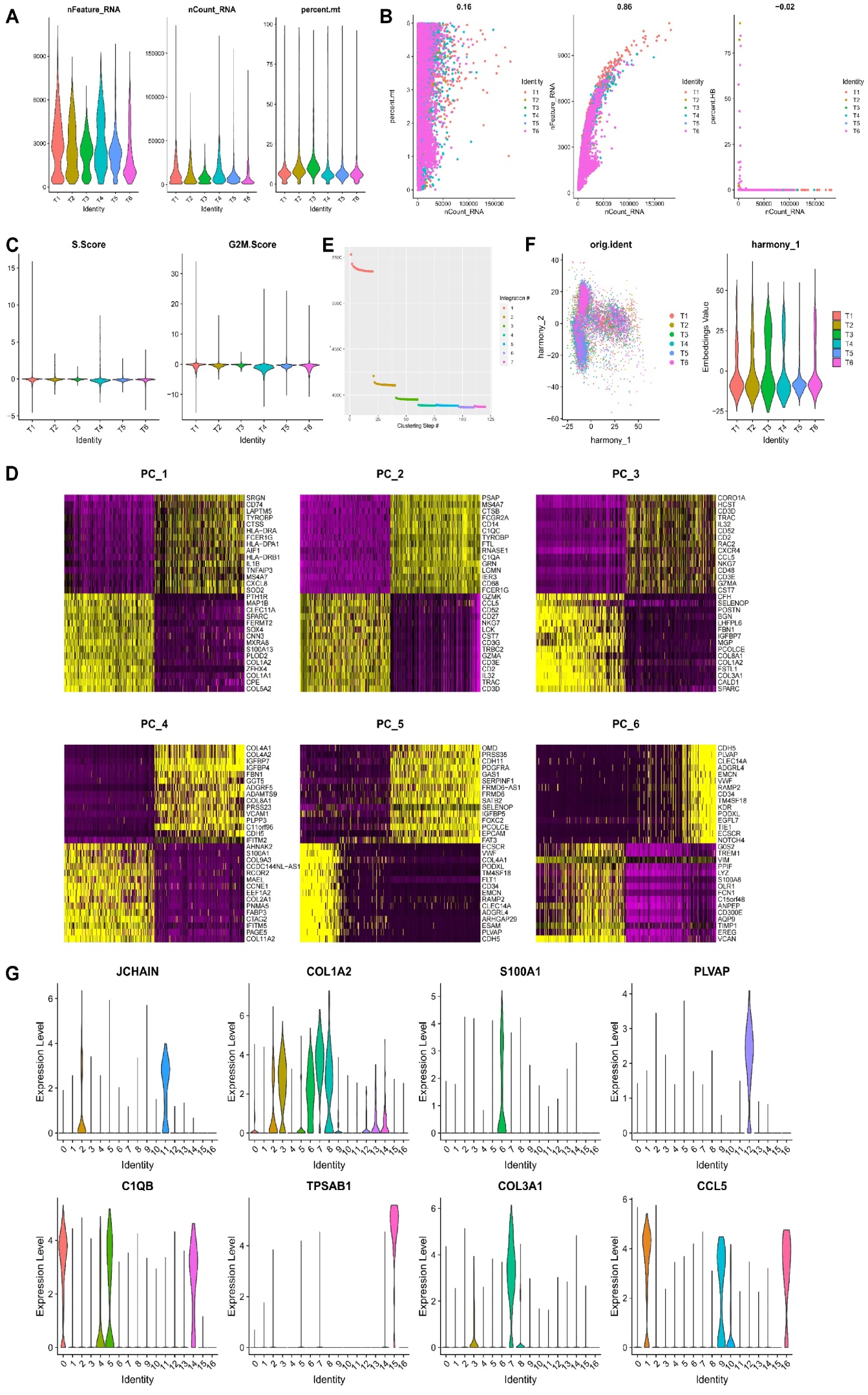
**

**Figure S1. Quality control and PCA dimensionality reduction of scRNA-seq data.**

Note: (A) Violin plots showing the number of genes per cell (nFeature_RNA), the number of mRNA molecules (nCount_RNA), and the percentage of mitochondrial genes (percent.mt) in scRNA-seq data (N=6); (B) Scatter plots depicting the correlation between filtered data nCount_RNA and percent.mt, nCount_RNA and nFeature_RNA, and nCount_RNA and percent.HB (N=6); (C) Cell cycle status of each cell in scRNA-seq data, where S.Score represents the S phase and G2M.Score represents the G2M phase (N=6); (D) Heatmap showing the top 20 significantly correlated gene expressions in PCA's PC_1 - PC_6, with yellow indicating upregulated expression and purple indicating downregulated expression (N=6); (E) Batch correction process graph of Harmony, where the x-axis represents the number of interactive iterations; (F) Distribution of cells after batch correction in PC_1 and PC_2, where each point represents a cell; (G) Expression patterns of known cell lineage-specific marker genes in different clusters of OS samples (N=6).

**
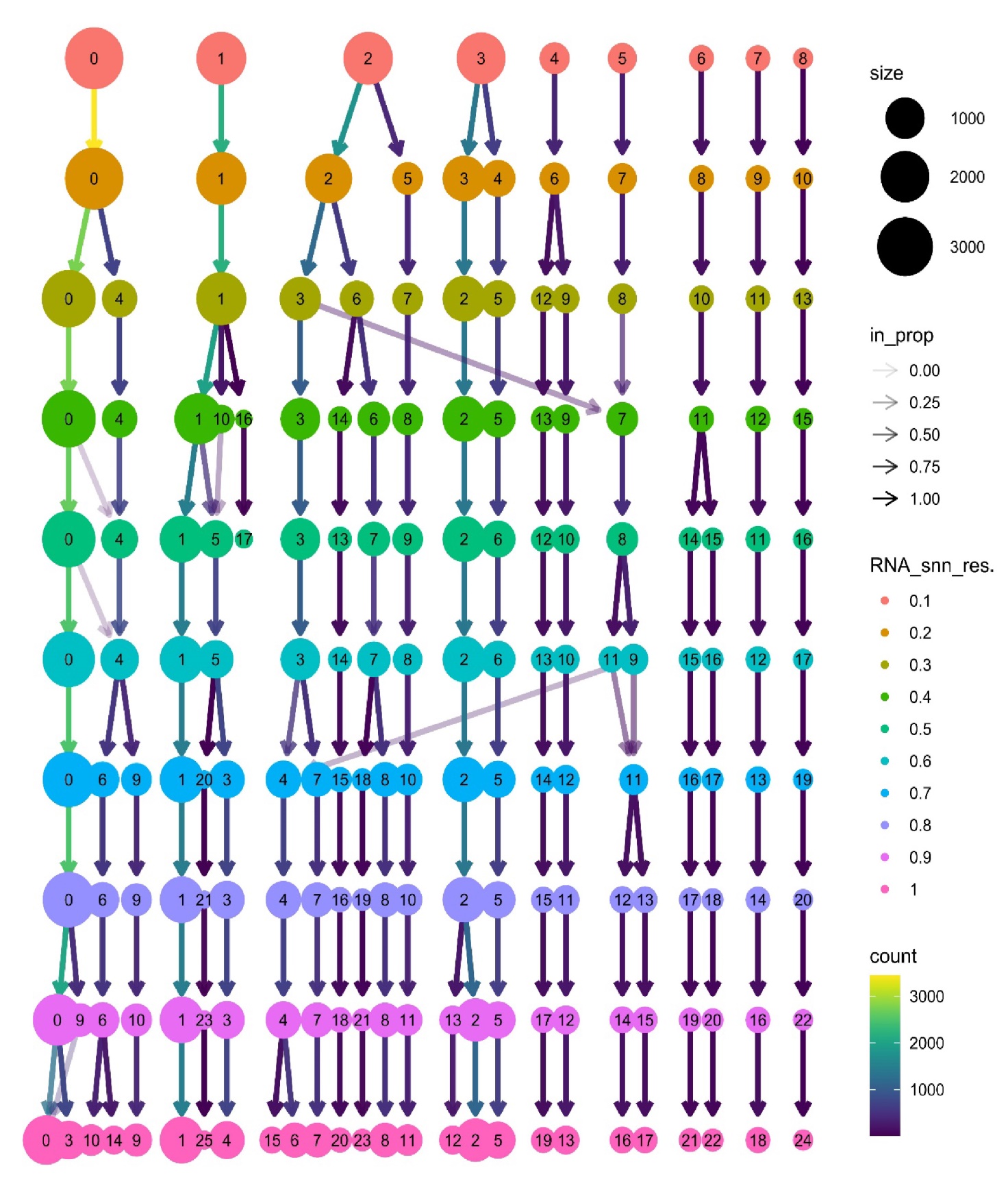
**

**Figure S2. UMAP clustering dendrogram of scRNA-seq data.**

**
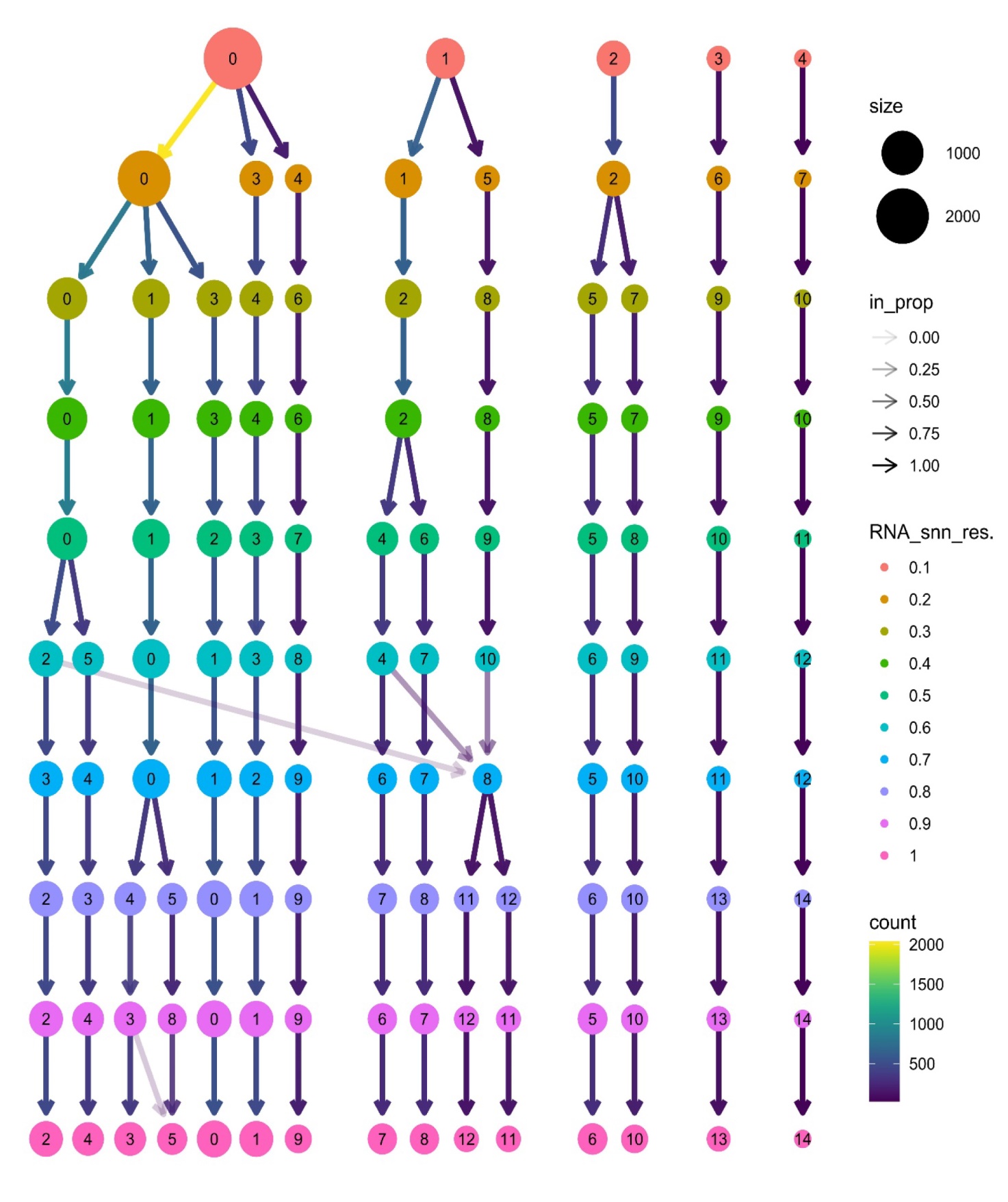
**

**Figure S3. UMAP clustering dendrogram of macrophage subtypes.**

**
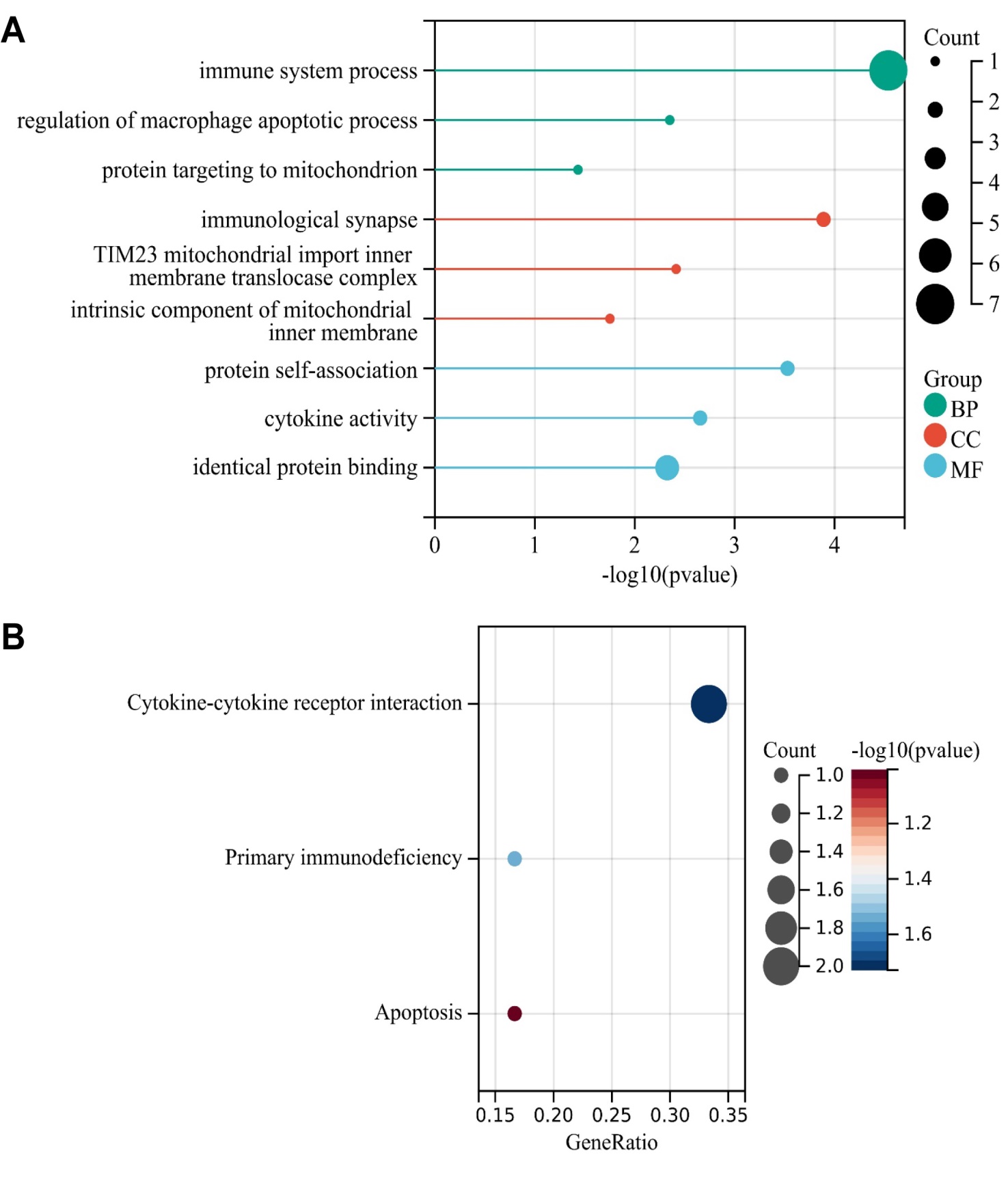
**

**Figure S4. Pathway enrichment analysis results of DEGs in macrophage subtypes.**

Note: (A) Bar plot showing the GO enrichment of DEGs between M1 Macrophages and M2 Macrophages in GSE162454 dataset, with green, red, and blue representing BP, CC, and MF, respectively; (B) KEGG bubble plot showing the enrichment of DEGs between M1 Macrophages and M2 Macrophages in GSE162454 dataset.

**
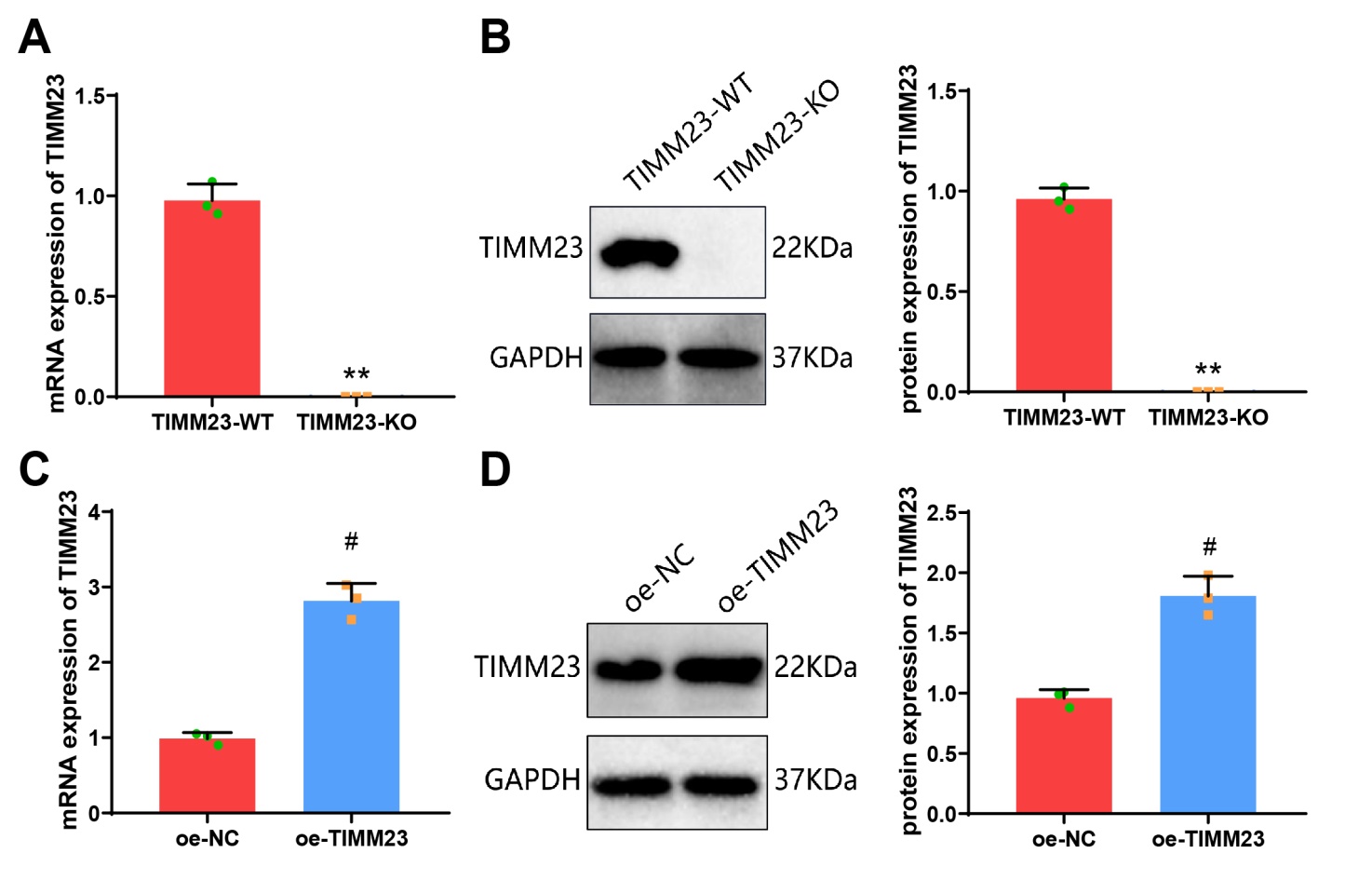
**

**Figure S5. Verification of TIMM23 knockdown and overexpression efficiency.**

Note: (A-B) mRNA and protein expression levels of TIMM23 in TIMM23-KO cells constructed by CRISPR/Cas9 gene editing technology, as detected by RT-qPCR and Western blot; (C-D) mRNA and protein expression levels of TIMM23 in cells overexpressing TIMM23, as detected by RT-qPCR and Western blot. * represents *p* < 0.05 compared to TIMM23-WT group, # represents *p* < 0.05 compared to oe-NC group. Cell experiments were repeated three times.

**
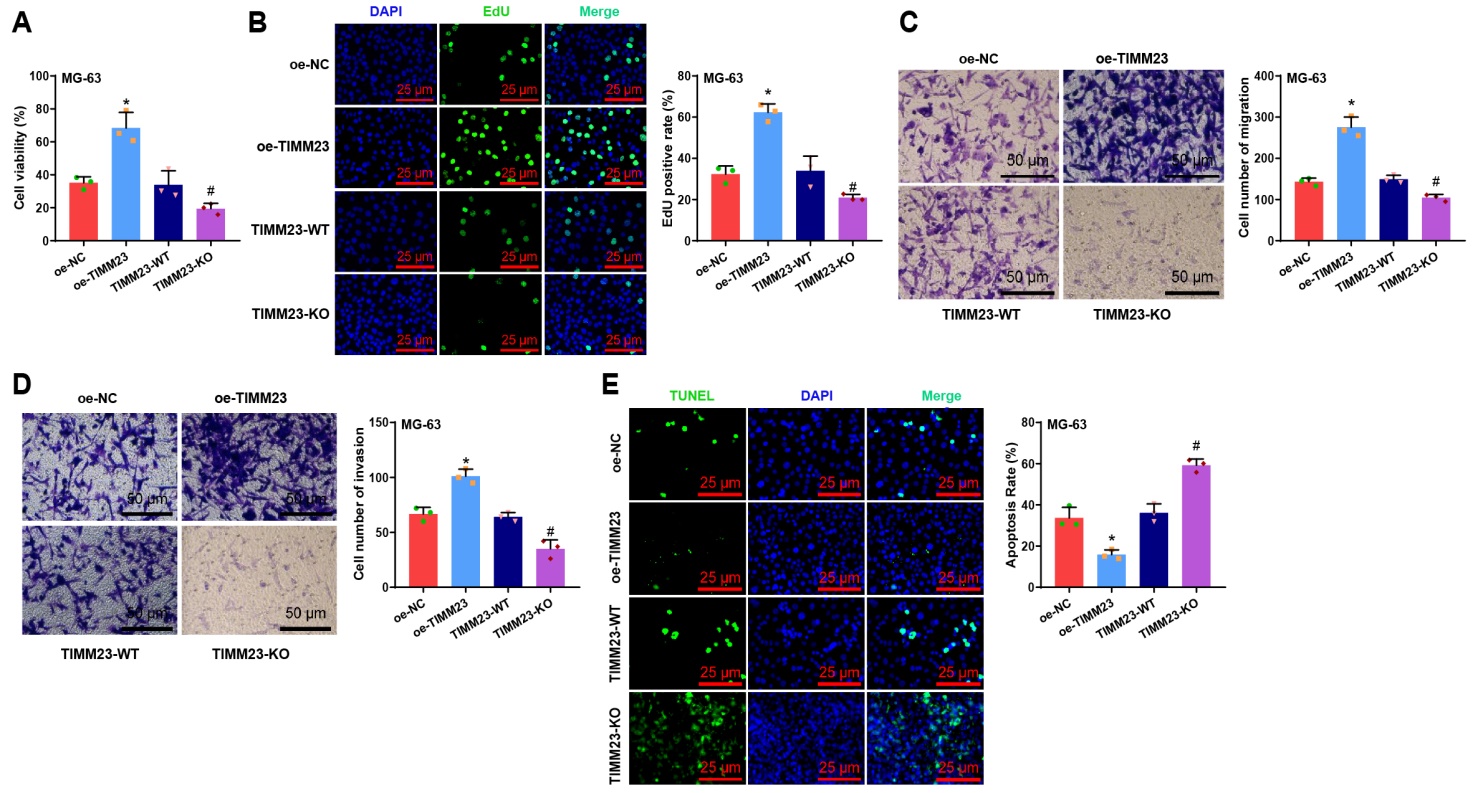
**

**Figure S6. Effects of TIMM23 on the biological functions of MG-63 cells.**

Note: (A) Cell viability of MG-63 cells in each group assessed using the MTT assay; (B) Proliferation ability of MG-63 cells in each group determined by EdU assay (scale bar: 25 μm); (C-D) Migration and invasion abilities of MG-63 cells in each group evaluated by Transwell assay (scale bar: 50 μm); (E) Apoptosis rate of MG-63 cells in each group detected by TUNEL assay (scale bar: 50 μm). * represents *p* < 0.05 compared to oe-NC group, # represents *p* < 0.05 compared to TIMM23-WT group. Cell experiments were repeated three times.

**
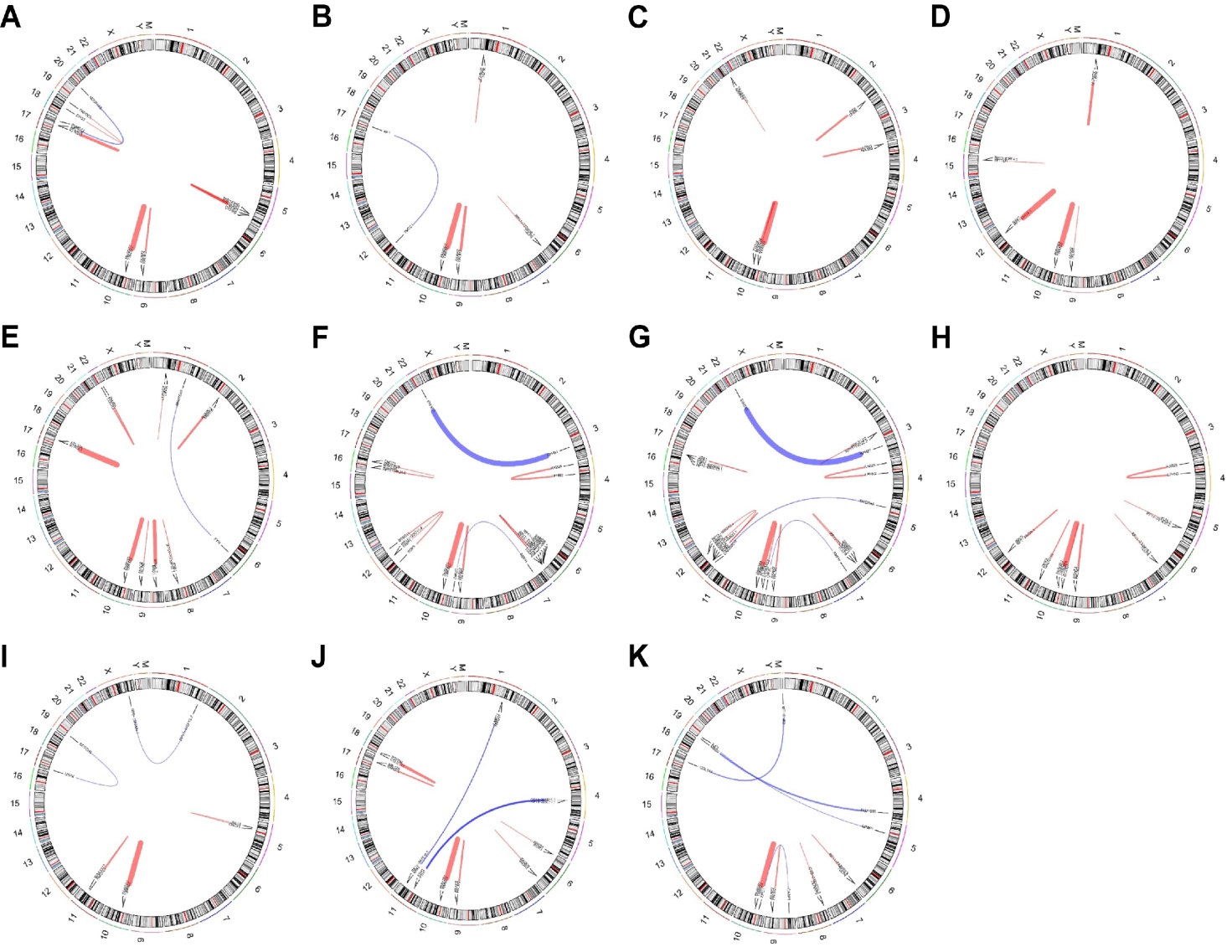
**

**Figure S7. Visualization of fusion gene analysis using chimeraviz.**

Note: Visualization results of fusion gene pairs in the SRR22577482-SRR22577492 dataset, where red bands represent intra-chromosomal fusions and blue bands represent inter-chromosomal fusions.

**
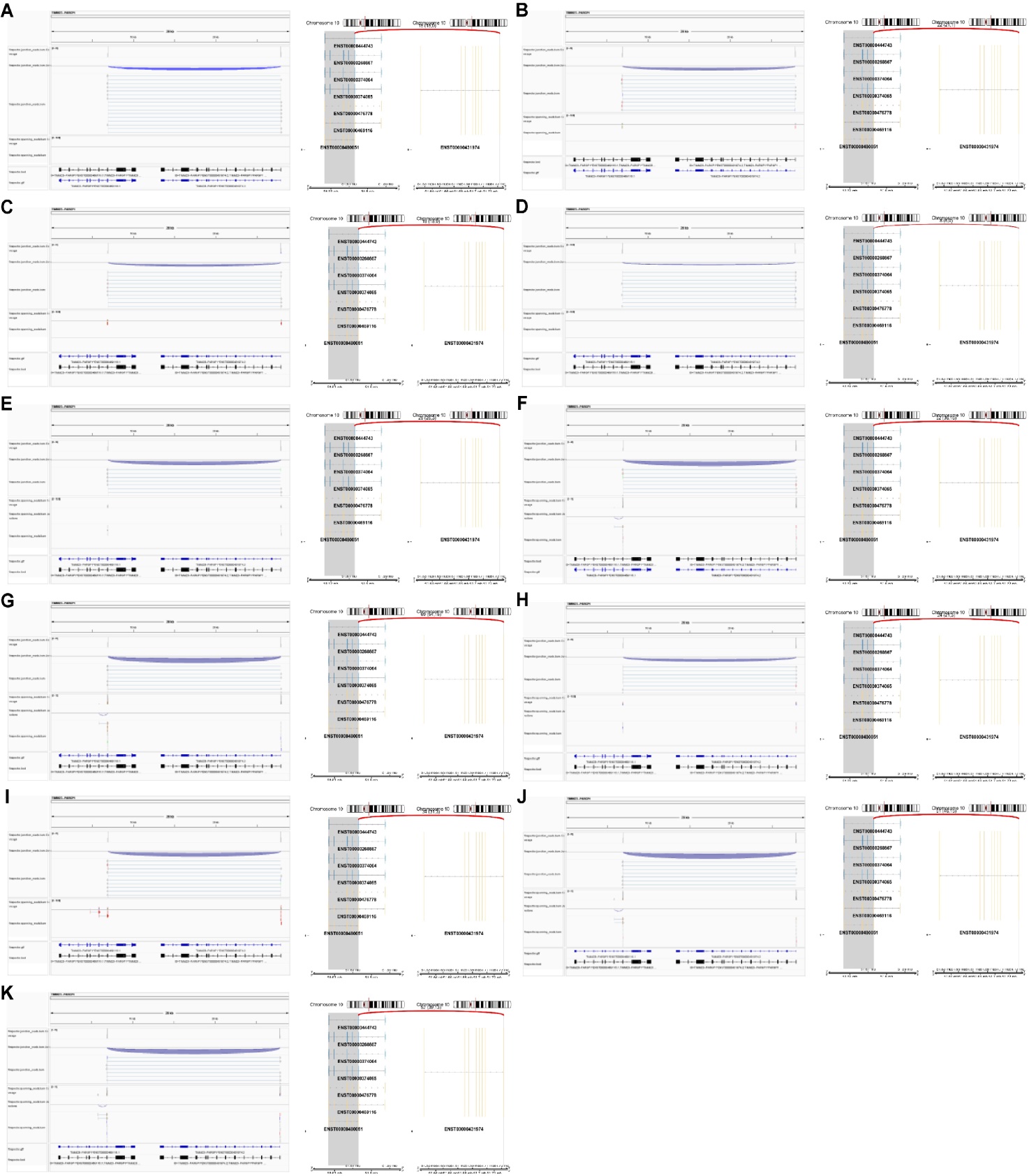
**

**Figure S8. Visualization of TIMM23-PARGP1 fusion gene pair.**

Note: Visualization results of TIMM23-PARGP1 fusion gene pair in the SRR22577482-SRR22577492 dataset, with the left side representing the visualization results using IGV software and the right side representing the visualization results using chimeraviz.

**
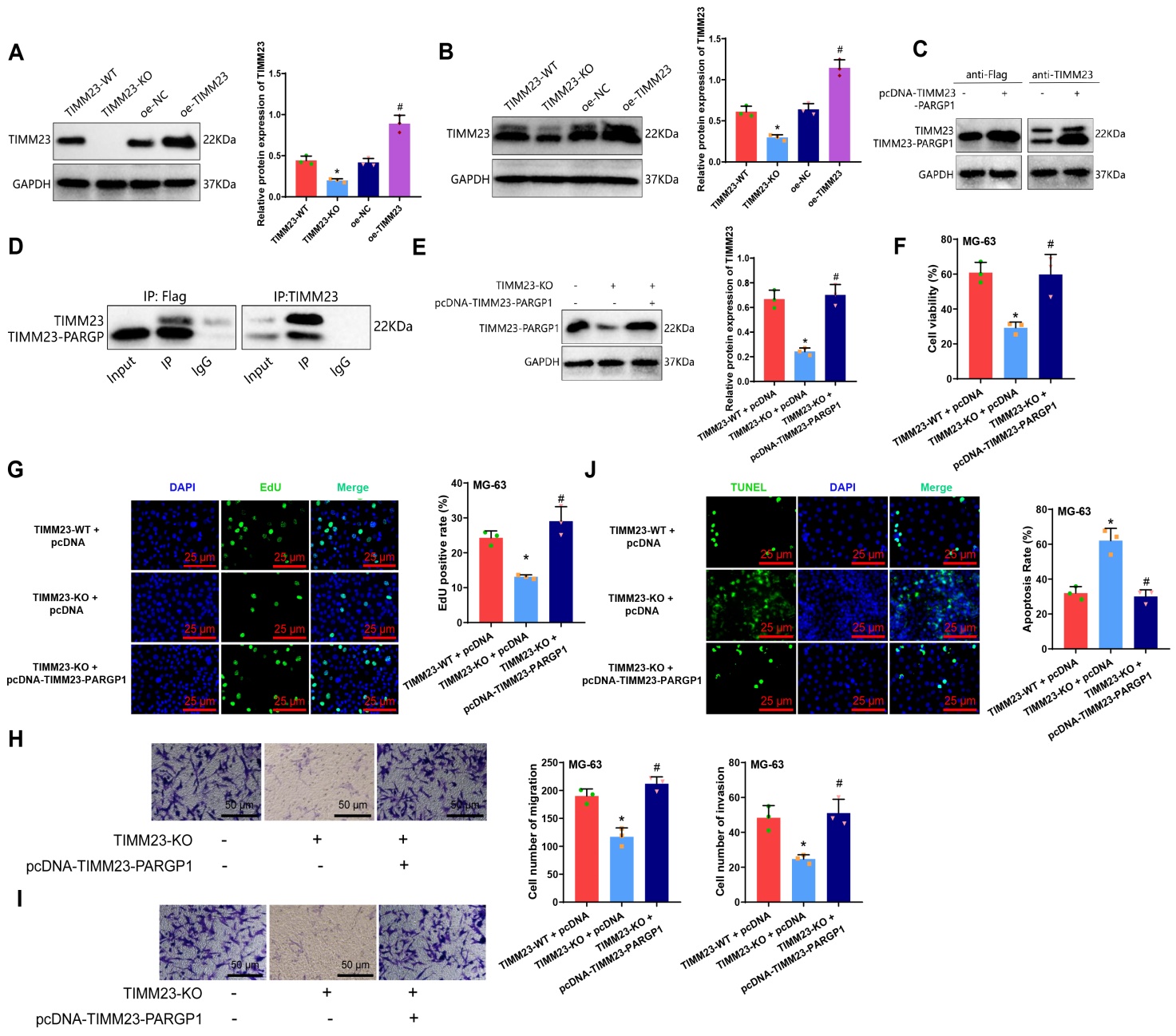
**

**Figure S9. Fusion gene detection and cell biology functional experiment results.**

Note: (A) Western blot analysis of TIMM23 protein expression in the MG-63 cells of co-culture models in each group; (B) Western blot analysis of TIMM23 and TIMM23-PARGP1 protein expression in the MG-63 cells of co-culture models in each group; (C) Western blot analysis of TIMM23 and TIMM23-PARGP1 protein expression in the MG-63 cells of co-culture models in each group; (D) Co-IP experiment to detect the interaction between TIMM23 and TIMM23-PARGP1; (E) Western blot analysis of TIMM23-PARGP1 protein expression in the MG-63 cells of co-culture models in each group; (F) MTT assay to evaluate the cell viability of MG-63 cells in each group; (G) EdU assay to evaluate the cell proliferation ability of MG-63 cells in each group (scale bar: 25 μm); (H-I) Transwell assay to evaluate the cell migration and invasion ability of MG-63 cells in each group (scale bar: 50 μm); (J) TUNEL assay to evaluate the apoptosis rate of MG-63 cells in each group (Scale bar=50 μm). * indicates *p* < 0.05 compared to the TIMM23-WT + pcDNA group, # indicates *p* < 0.05 compared to the TIMM23-KO + pcDNA group, all cell experiments were repeated 3 times.

**
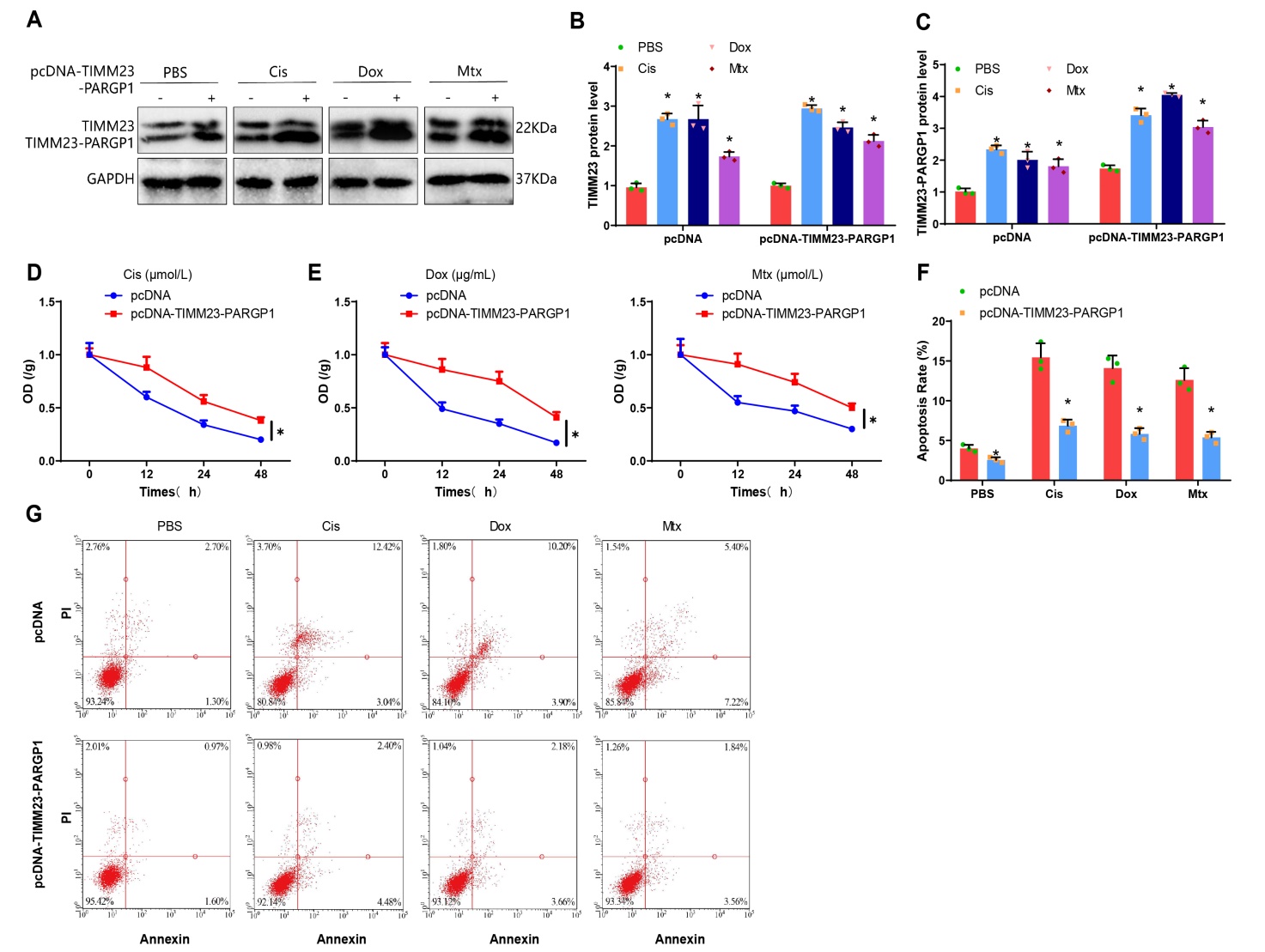
**

**Figure S10. Impact of TIMM23-PARGP1 on the chemoresistance of MG-63 cells.**

Note: (A-B) Western blot analysis of TIMM23 and TIMM23-PARGP1 protein expression in different groups of MG-63 cells; (C-E) CCK-8 assay to assess the cell viability of MG-63 cells after chemotherapy treatment; (F-G) Flow cytometry analysis to detect the apoptosis of MG-63 cells after chemotherapy treatment. * indicates *p* < 0.05 compared to the pcDNA group or PBS group. Cell experiments were repeated 3 times.

**
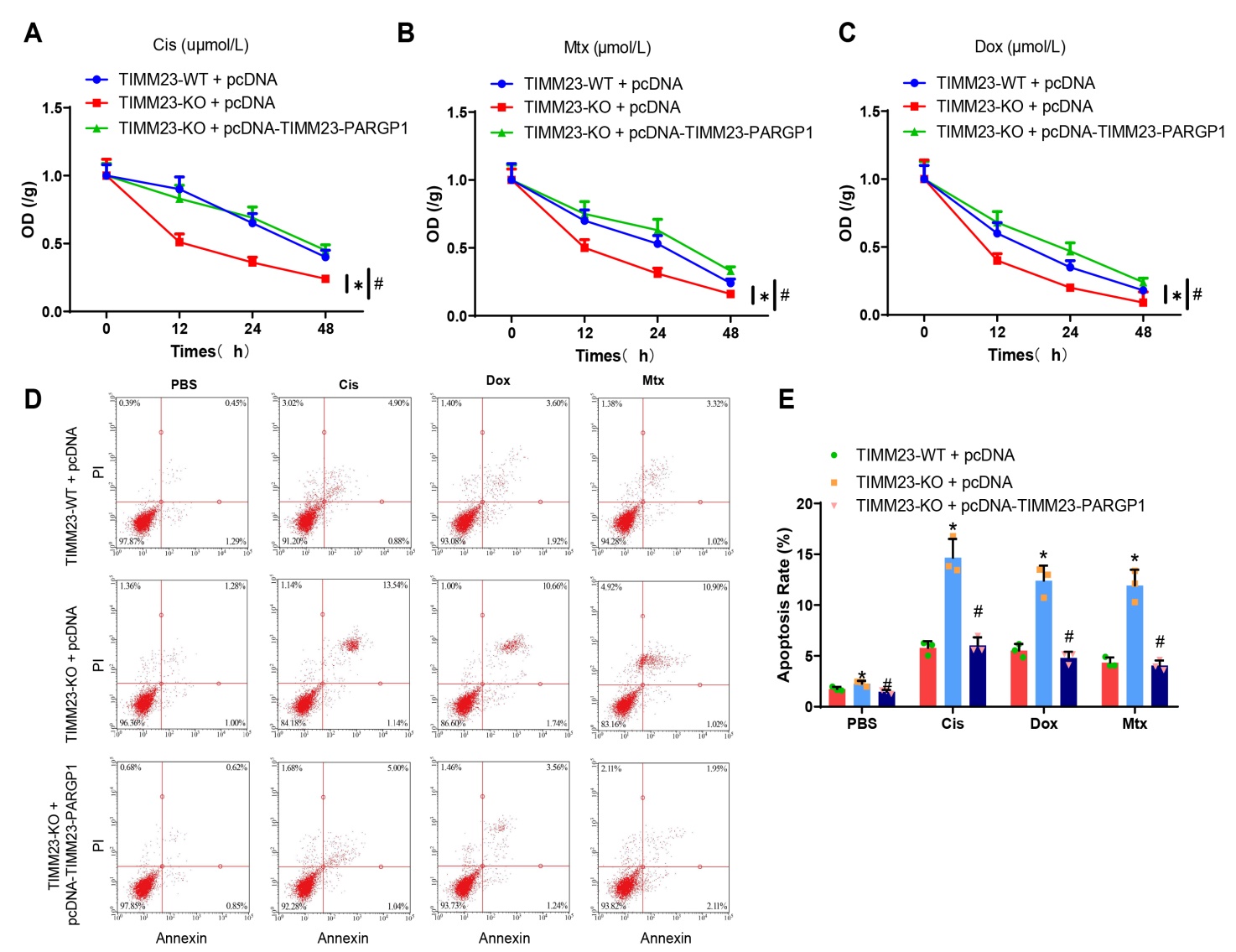
**

**Figure S11. Impact of TIMM23 and TIMM23-PARGP1 on the chemoresistance of MG-63 cells.**

Note: (A-C) CCK-8 assay to evaluate the cell viability of MG-63 cells in each group after chemotherapy treatment; (D-E) Flow cytometry analysis to detect the apoptosis of MG-63 cells in each group after chemotherapy treatment. * indicates *p* < 0.05 compared to the TIMM23-WT + pcDNA group, # indicates *p* < 0.05 compared to the TIMM23-KO + pcDNA group, all cell experiments were repeated 3 times.

**
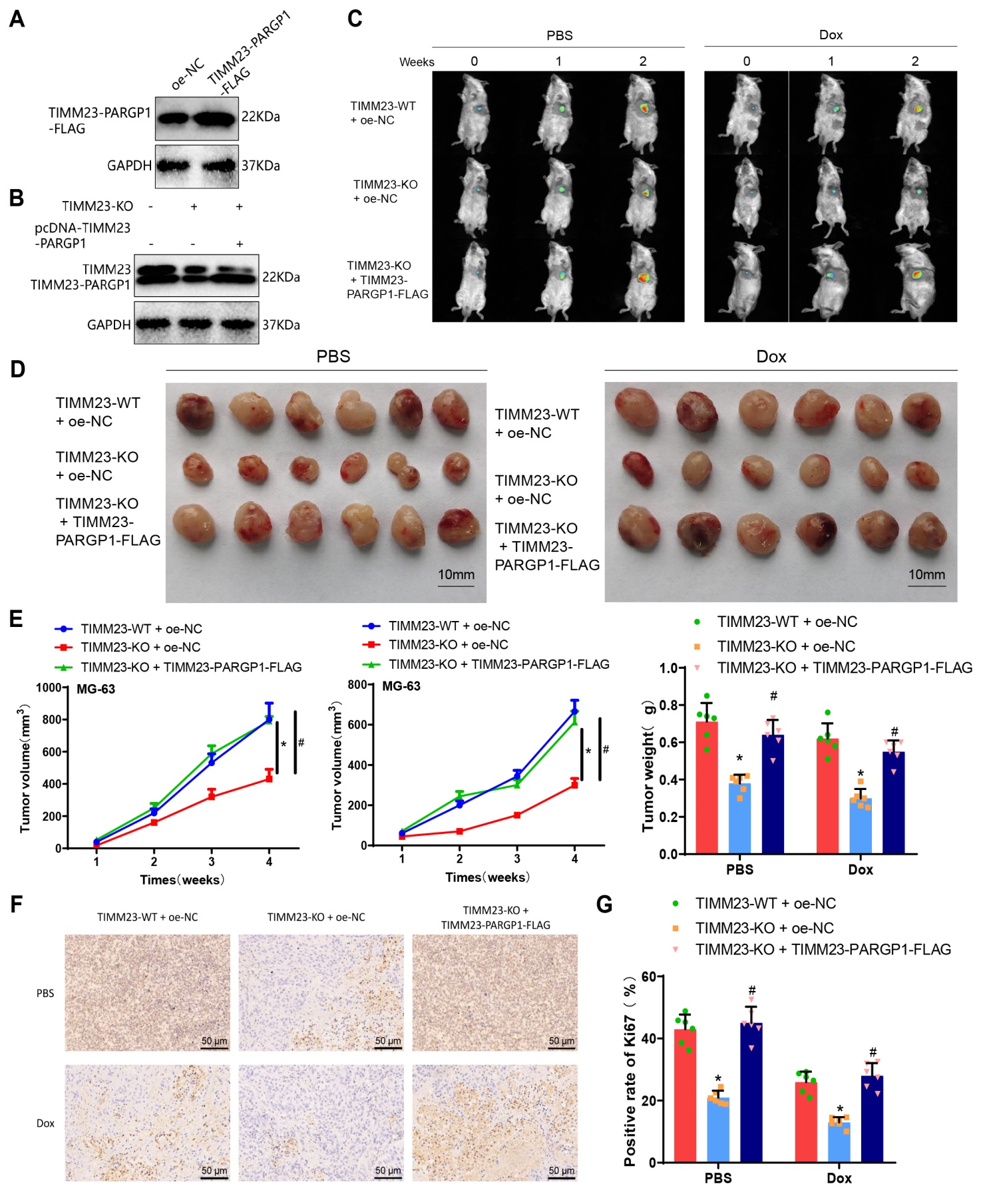
**

**Figure S12. Impact of TIMM23-mediated M2 macrophage polarization on the production of TIMM23-PARGP1 in MG-63 cells and its effect on *in vivo* tumorigenesis.**

Note: (A) Western blot analysis of overexpressed TIMM23-PARGP1-FLAG protein in cells, each experiment was repeated 3 times; (B) Western blot analysis of TIMM23 and TIMM23-PARGP1 protein expression levels in mouse tumor tissues; (C) Monitoring tumor growth at different time points using bioluminescence intensity detection, with one representative example shown for each group; (D) Morphology of mouse tumor tissues in each group; (E) Growth of mouse tumor tissues in each group; (F) Weight of mouse tumor tissues in each group; (G) Immunohistochemistry staining to detect Ki67 protein expression in mouse tumor tissues (scale bar: 50 μm). * indicates *p* < 0.05 compared to the TIMM23-WT + oe-NC group, # indicates *p* < 0.05 compared to the TIMM23-KO + oe-NC group, each group consisted of 6 mice.

**Table S1. Cell types and markergenes**

| **Cell Type** | **Markergenes** |
| --- | --- |
| B Cell | JCHAIN, DERL3, IGHG1, MS4A1 |
| Bone Marrow Mesenchymal Stem Cells | COL1A2, ALPL, IBSP, RUNX2 |
| Cancer Cell | S100A1, NDUFA4L2 |
| Endothelial Cell | PLVAP, RAMP2, DNASE1L3 |
| Macrophage | C1QB, C1QC, APOE, IL1B |
| Mast Cell | TPSAB1, TPSB2, CPA3 |
| Progenitor Cell | COL3A1, MGP, ASPM, MMP9 |
| T Cell | CCL5, IL32, TNFRSF18 |

**Table S2. Cell types and marker genes of macrophages**

| **Cell Type** | **Marker Genes** |
| --- | --- |
| M0 macrophage | CD14, CD68, MERTK |
| M1 macrophage | CCL5, CD86, CD80 |
| M2 macrophage | IL10, CD36, CD163 |

**Table S3. RT-qPCR primer sequences**

| **Gene** | **Primer Sequence** |
| --- | --- |
| GAPDH (human) | F: 5'-CGGATTTGGTCGTATTGGGC-3' |
|  | R: 5'-TTGACGGTGCCATGGAATTTG-3' |
| TIMM23 (human) | F: 5'- ACACGAGGTGCAGAAGATGA -3' |
|  | R: 5'- ATCCCTCGAAGACCACCTGT -3' |

Note: F: Forward; R: Reverse.

**Table S4. Details of the first antibody product**

| **Name** | **Cat.** | **Dilution Ratio** | **Manufacturer** | **Country** | **MW (kDa)** |
| --- | --- | --- | --- | --- | --- |
| GAPDH | ab9485 | 1: 2500 | Abcam | UK | 37 |
| TIMM23 | ab230253 | 1: 1000 | Abcam | UK | 22 |
| PINK1 | ab216144 | 1: 1000 | Abcam | UK | 63 |
| Parkin | ab77924 | 1: 2000 | Abcam | UK | 52 |
| p-Parkin | PA5-114616 | 1: 2000 | Invitrogen | USA | 52 |
| LC3II/I | PA5-22731 | 1: 1000 | Invitrogen | USA | 15/17 |
| Flag (DDDDK) | ab205606 | 1: 500 | Abcam | UK | / |
